# Constitutionally Selective Dynamic Covalent Nanoparticle Assembly

**DOI:** 10.1021/jacs.2c05446

**Published:** 2022-07-28

**Authors:** Nicolas Marro, Rongtian Suo, Aaron B. Naden, Euan R. Kay

**Affiliations:** EaStCHEM School of Chemistry, University of St Andrews, North Haugh, St Andrews, KY16 9ST, U.K.

## Abstract

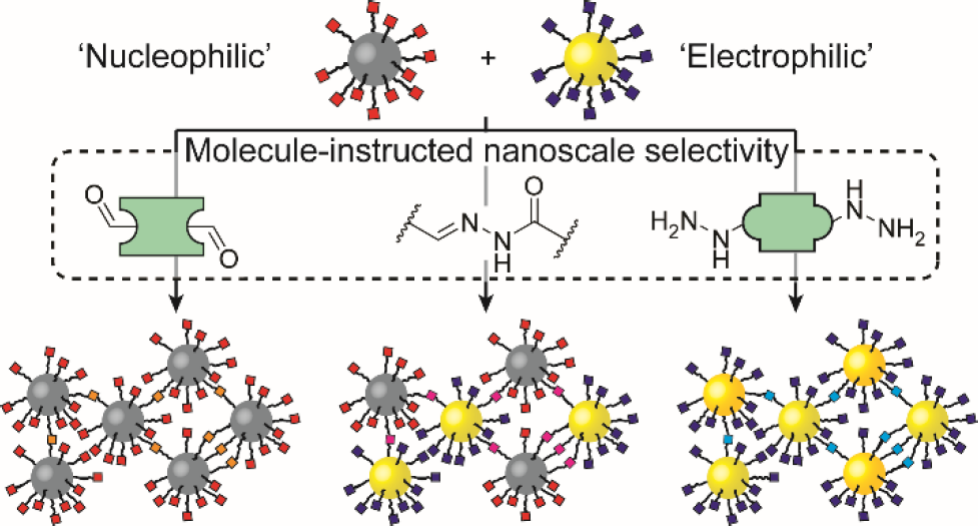

The future of materials chemistry will be defined by our ability to precisely arrange components that have considerably larger dimensions and more complex compositions than conventional molecular or macromolecular building blocks. However, exerting structural and constitutional control in the assembly of nanoscale entities presents a considerable challenge. Dynamic covalent nanoparticles are emerging as an attractive category of reaction-enabled solution-processable nanosized building block through which the rational principles of molecular synthetic chemistry can be extended into the nanoscale. From a mixture of two hydrazone-based dynamic covalent nanoparticles with complementary reactivity, specific molecular instructions trigger selective assembly of intimately mixed heteromaterial (Au–Pd) aggregates or materials highly enriched in either one of the two core materials. In much the same way as complementary reactivity is exploited in synthetic molecular chemistry, chemospecific nanoparticle-bound reactions dictate building block connectivity; meanwhile, kinetic regioselectivity on the nanoscale regulates the detailed composition of the materials produced. Selectivity, and hence aggregate composition, is sensitive to several system parameters. By characterizing the nanoparticle-bound reactions in isolation, kinetic models of the multiscale assembly network can be constructed. Despite ignoring heterogeneous physical processes such as aggregation and precipitation, these simple kinetic models successfully link the underlying molecular events with the nanoscale assembly outcome, guiding rational optimization to maximize selectivity for each of the three assembly pathways. With such predictive construction strategies, we can anticipate that reaction-enabled nanoparticles can become fully incorporated in the lexicon of synthetic chemistry, ultimately establishing a synthetic science that manipulates molecular and nanoscale components with equal proficiency.

## Introduction

Molecular synthesis has moved far beyond the manipulation of individual functional groups, to the extent that increasing structural sophistication is no longer regarded as the foremost challenge.^1−3^ Guided by structure–reactivity understanding and with a vast array of optimized transformations to select from, covalent bonds can be manipulated with exquisite selectivity. Through the logical conceptual framework of retrosynthetic planning,^4,5^ strategies to access myriad complex molecular structures have been devised from a relatively small set of commercially available molecular reagents.^6^ Nevertheless, it remains to be shown whether the same principles—taken for granted on molecular length scales—can be extended to assemble structures on length-scales orders of magnitude greater than the covalent bond.

Rational and selective methods for connecting nanoscale building blocks will open up for exploration of a largely uncharted region of chemical space where new collective and emergent properties can be found; it will enable the bottom-up construction of nanostructured materials and will allow nanomaterials to be interfaced with existing technologies.^7−12^ Yet, any methods must address complexity arising from multiple competing chemical and physical processes; solution-phase and surface-confined reaction environments; multivalency; dispersity in parameters including size, shape, and composition; and allied analytical challenges. Monolayer-stabilized nanoparticles, defined by a nanosized core stabilized by a shell of (macro)molecular ligands,^13−17^ are solution-dispersible hybrid nanomaterials that can typically be manipulated and analyzed using the familiar methods and infrastructure of synthetic chemistry. Consequently, postsynthesis elaboration through selective and stimuli-responsive chemical interactions at the monolayer periphery can provide efficient, divergent strategies for structural and functional diversification, as well as integration with other components and assembly of nanoparticle building blocks.

Several approaches have been explored for linking nanoparticles through covalent or noncovalent interactions between surface-bound molecules, including a number that achieve responsive and reversible control over the assembly state.^18−24^ However, combining more than one type of nanoparticle component presents a further level of challenge, and selective coupling in multicomponent mixtures is rarely seen.^18−24^ The broad structural and compositional diversity now accessible for colloidal nanoparticles suggests vast potential for tuning composite material properties if multiple types of instructable nanoscale building block can be connected with high selectivity.

The exquisite specificity of base-pairing interactions has underpinned the oligonucleotide-programmed assembly of colloidal nanoparticles to produce a huge variety of organized structures,^8,23,25−29^ including combinations of building blocks with different sizes, shapes, or core material types. Whereas biomolecular linkers are restricted to a relatively narrow window of environmental conditions required to maintain their structural and functional integrity,^30−32^ abiotic mediators confer a higher degree of customizability; the full gamut of synthetic molecular architectures can be exploited for tuning structure, function, and properties to match a variety of settings.^12,24^ Although many strategies can be applied to mixtures of building blocks, only a handful of pioneering examples have achieved any degree of compositional selectivity other than through rudimentary variation of the building block feed ratio. Redox-switchable pseudorotaxane links between nanoparticle-bound guests and a multivalent polymeric linker have been exploited for the selective capture and release of either gold or silver nanoparticles from a binary mixture.^33^ Wavelength-selective manipulation of dipole–dipole interactions has been harnessed to reversibly assemble distinct unary aggregates from a binary mixture of gold nanoparticles functionalized with different azobenzene photoswitches.^34^ Tuning the protonation state of surface-bound amines on titania nanoparticles has been used to direct formation of unary aggregates mediated by host–guest interactions with macrocyclic linkers, or heteromaterial assembly with aldehyde-bearing gold nanoparticles.^35^ In a rare all-covalent example, a stepwise process applying a judiciously chosen bifunctional metal-surface-coordinating small-molecule linker to colloidal solutions of gold and iron oxide nanoparticles produced binary heteromaterial assemblies with either intimately mixed or phase-separated morphology but with no compositional selectivity.^36^ Most recently, orthogonally addressable coordination interactions and complementary hydrogen bonding arrays have been used to direct gold nanoparticles of two different core sizes to form either intimately mixed or phase segregated morphologies, which can subsequently be cross-linked by covalent bonds.^37^

**Figure 1:**
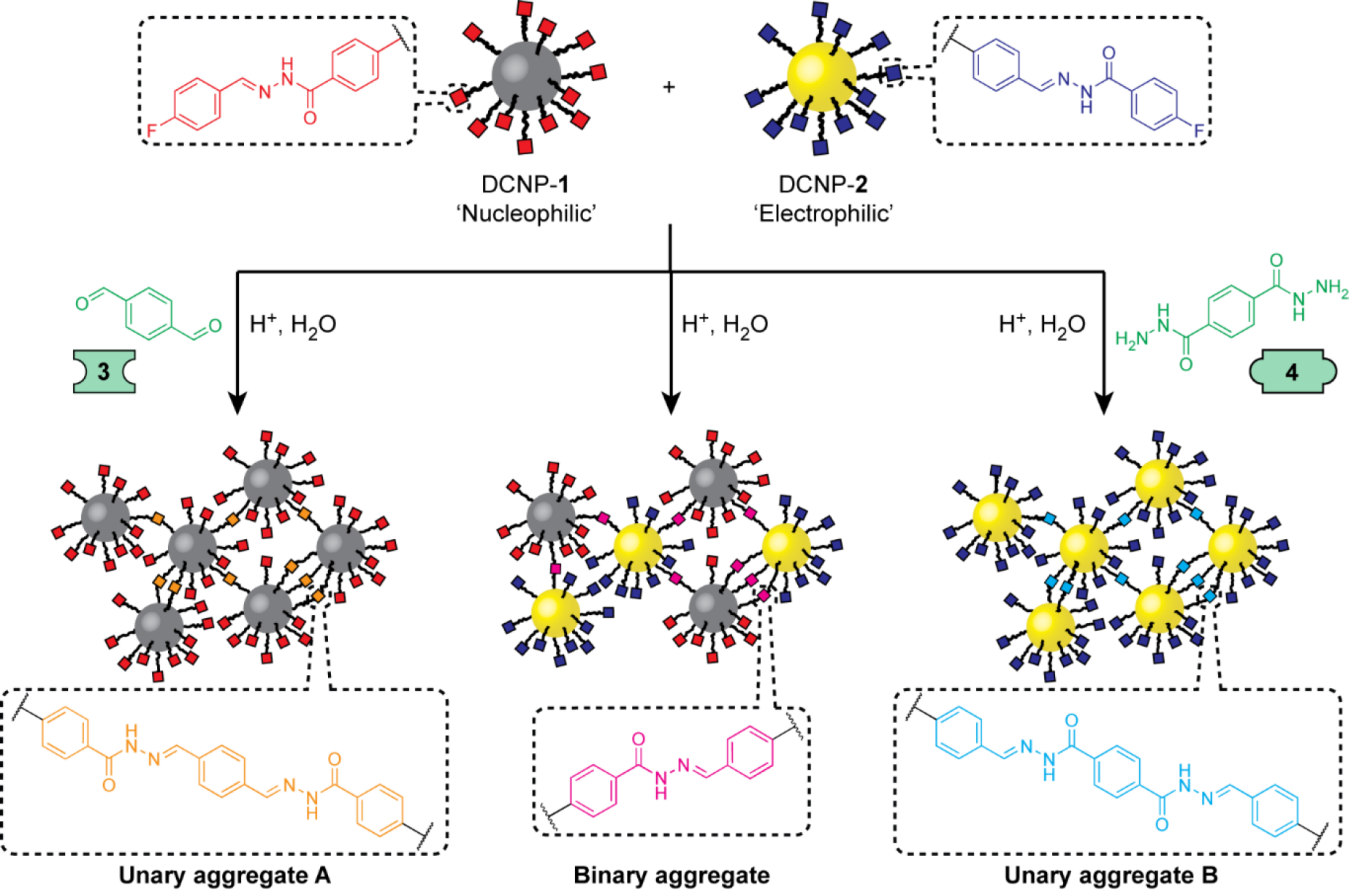
Selective assembly of binary or unary aggregates from two complementary dynamic covalent nanoparticle (DCNP) building blocks. Nucleophilic DCNP-**1** was constructed using either gold cores (AuNP-**1**) to give homomaterial binary aggregates or on palladium cores (PdNP-**1**) to give heteromaterial binary aggregates in combination with electrophilic DCNP-**2**, which were constructed on gold cores (AuNP-**2**).

Dynamic covalent linkages have recently shown promise for producing adaptive but robust all-covalent nanoparticle assemblies,^38,39^ through either reversible reactions between nanoparticle-bound molecules and bifunctional molecular linkers^40,41^ or direct reaction between complementary nanoparticle-bound functionalities.^35,42^ Here, we demonstrate a system that combines both modes of dynamic covalent linking to selectively instruct the assembly of two nanoscale building blocks down any one of the three pathways that are available from a binary mixture starting point (Figure 1).

Invoking the molecular synthetic principles of chemospecificity and regioselectivity, a pair of dynamic covalent nanoparticles (DCNPs),^38,43,44^ stabilized by chemically complementary reactive monolayers^42^ on two different core materials, can be selectively coupled to produce constitutionally controlled aggregates. By appropriate choice of reaction conditions, aggregates highly enriched in either one of the core materials can be selected, or binary heteromaterial compositions can be produced with intimately mixed internal morphology (Figure 1). Although the assembly processes are triggered by molecular events under thermodynamic control, kinetic effects on the nanoscale play a critical role in determining the assembly pathway selectivity. Furthermore, dynamic covalent linkages can be rendered kinetically stable by removing the conditions for exchange, so that any of the covalently linked materials are equally robust. Characterizing the on-particle molecular reactions responsible for driving nanoparticle assembly can guide optimization of the nanoscale construction process through simulations of simple kinetic models. Such rational behavior, which is underpinned by quantitative molecular-level understanding, suggests that DCNPs can be selectively reactive building blocks through which nanoscale synthetic methods might one day approach the high standards set by contemporary molecular synthetic chemistry.

## Results and Discussion

We recently developed a pair of dynamic covalent AuNPs, each stabilized by a single-component monolayer terminated with a hydrazone that is arranged to present either a “nucleophilic” or an “electrophilic” surface-tethered reactive site.^42,45^ This pair of metallic nanoparticle building blocks may each undergo chemospecific monolayer modifications on reaction with either electrophilic or nucleophilic molecular modifiers. Furthermore, their complementary and reversible reactivity opens the door to chemospecific reactions directly between nanoscale building blocks to create nanoparticle assembles endowed with the adaptive characteristics of the underlying dynamic covalent links.^42^ We therefore envisaged that these attributes could be harnessed to direct the covalently linked assembly of either unary homomaterial or binary heteromaterial aggregates, directed by applying specific triggers to a mixture of two distinct reactive nanoparticle building blocks (Figure 1).

## Linker-Driven Assembly of Nucleophilic and Electrophilic DCNPs

**Figure 2:**
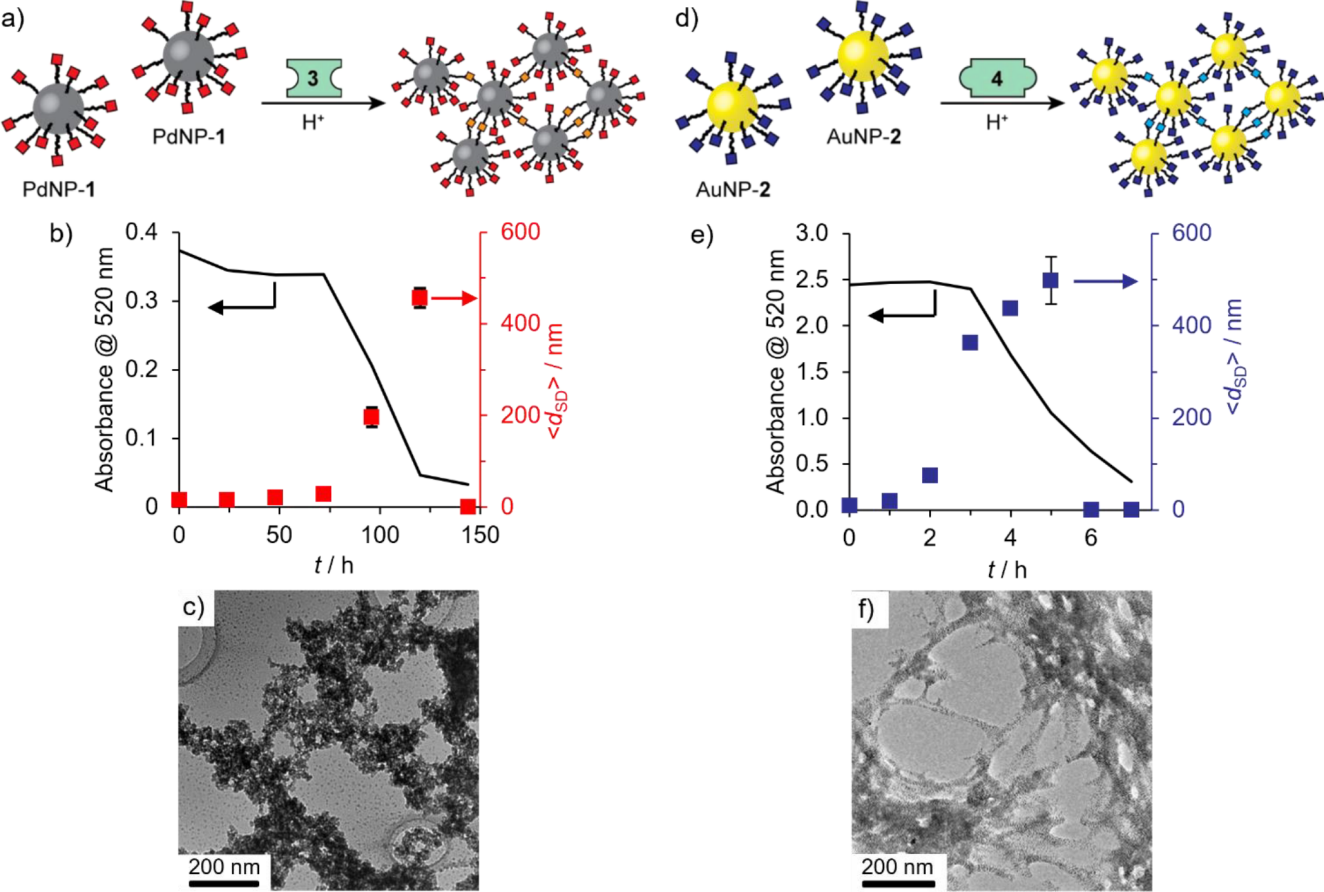
(a–c) Linker-driven assembly of nucleophilic PdNP-**1** using dialdehyde linker **3**. (d–f) Linker-driven assembly of electrophilic AuNP-**2** using dihydrazide linker **4**. (b, e) Solvodynamic diameter (⟨*d*_SD_⟩, red symbols and blue symbols) as measured by DLS and absorbance at 520 nm (solid lines) evolution over time after addition of the acid catalyst required for dynamic covalent hydrazone exchange. (c, f) Representative TEM images of PdNP aggregates (c) and AuNP aggregates (f) isolated after assembly of all nanoparticle material. For supplementary TEM images, see Figures S11 and S14, respectively. Conditions (concentrations in terms of molecular species) (a–c): [PdNP-**1**]_0_ = 0.15 mM, [**3**]_0_ = 0.45 mM, [CF_3_CO_2_H]_0_ = 20 mM, 9:1 v/v DMF/H_2_O, room temperature; (d–f): [AuNP-**2**]_0_ = 0.15 mM, [**4**]_0_ = 0.45 mM, [CF_3_CO_2_H]_0_ = 20 mM, 9:1 v/v DMF/H_2_O, room temperature.

To establish the assembly of DCNP building blocks triggered by chemospecific reaction with complementary molecular linkers, nucleophilic and electrophilic dynamic covalent hydrazones were installed on palladium and gold nanoparticle cores, respectively (PdNP-**1**, AuNP-**2**, Figure 1; AuNP-**1** were also prepared to allow monitoring of assembly by UV–vis absorption spectroscopy, see Supporting Information). The assembly of each nanoparticle on introduction of a chemically complementary homobifunctional molecular linker (Figure 2a,d) was then assessed. On treating a dark green solution of PdNP-**1** (0.15 mM in terms of hydrazone ligand)^46^ with dialdehyde linker **3** (0.45 mM, 3 mol equiv with respect to nanoparticle-bound **1**) in the presence of water and a Brønsted acid catalyst (20 mM CF_3_CO_2_H), extended aggregates appeared as precipitates after 6 days, leaving a colorless supernatant. Monitoring by dynamic light scattering (DLS, Figure 2b, red symbols) revealed intermediate stages in the assembly process. After 3 days, an increase in the average solvodynamic size measured by DLS (⟨*d*_SD_⟩ = 28 nm) indicates the onset of cluster formation. Steady growth in the size of colloidally stable aggregates was then observed, reaching ⟨*d*_SD_⟩ = 457 nm at day 5, following which complete precipitation had occurred by day 6. Although PdNPs of this size do not possess a strong surface plasmon resonance, the assembly process could also be tracked by UV–vis absorption spectroscopy (Figure 2b, solid line), which indicated a sharp decrease in absorption at all wavelengths during days 3–5, coincident with the emergence of aggregates by DLS. Imaging of the precipitates by transmission electron microscopy (TEM, Figures 2c and S11) revealed almost quantitative assembly of nanoparticles into extended aggregates. Gold-core nanoparticle analogue AuNP-**1** assembled with linker **3** in a very similar fashion (see Supporting Information). Steady growth of colloidally stable aggregates between days 4 and 8 was observed by DLS (Figure S12), with complete precipitation at day 9 to give extended aggregates of almost identical morphology to the PdNP analogues (Figure S13). The strong plasmon resonance of gold revealed more detail on simultaneous monitoring of the assembly process by UV–vis absorption spectroscopy (Figure S12). Only a very slight decrease in absorption at the surface plasmon resonance (λ_max_ = 520 nm) was observed up until day 8, indicating that the majority of clusters remain colloidally stable during this stage of the process. By day 8, the average cluster size reached ⟨*d*_SD_⟩ = 253 nm, following which complete precipitation occurred at day 9. For either nanoparticle, no aggregation or precipitation was observed in the absence of either a chemically complementary linker or the acid catalyst required for dynamic covalent exchange (Figures S15–S18), confirming the critical role of chemospecific particle–linker hydrazone exchange.

Electrophilic AuNP-**2** could be assembled in a similar manner using complementary nucleophilic dihydrazide molecular linker **4** (Figure 2d). In this case, assembly and precipitation occurred over a much shorter time scale (compare Figures 2b, e). After only 1 h, DLS readings (Figure 2e, blue symbols) indicated an increase in solvodynamic size to ⟨*d*_SD_⟩ = 19 nm (from the fully dispersed value of ⟨*d*_SD_⟩ = 8.9 nm at *t* = 0 h). Cluster growth continued rapidly, reaching ⟨*d*_SD_⟩ = 498 nm after only 5 h, followed by complete precipitation leaving no colloidally stable particles detectable by DLS after 6 h. UV–vis absorption measurements (Figure 2e, solid line) indicated no significant loss of material from solution over the first 3 h, followed by rapid precipitation to leave virtually no colloidally stable material after 7 h. At this time point, TEM images revealed almost complete assembly of nanoparticles into extended networks (Figures 2f and S14a), which were shown to have three-dimensional nature by SEM imaging (Figure S14b). Control experiments revealed no assembly over the same time period in the absence of either the acid catalyst or linker **4** (Figures S19–S20), again confirming the chemospecifc nature of the molecule-driven assembly process.

The rapid assembly of AuNP-**2** is in stark contrast to much slower kinetics for previously reported dynamic covalent nanoparticle assembly systems,^40,41^ some of which even require preassembly of the reactant nanoparticles via noncovalent interactions prior to covalent cross-linking.^37^ The significantly faster assembly of AuNP-**2** in the presence of linker **4**, compared to PdNP-**1** linked by **3**, directly reflects the underlying molecular reaction kinetics for nanoparticle-bound dynamic covalent exchange, which we have previously studied in detail using monofunctional exchange units.^42^ This provides strong evidence that the same molecular processes are responsible for driving aggregation of the nanoscale building blocks, underlining the value of careful molecular-level characterization for rationalizing nanoscale behavior. The faster aggregation of AuNP-**2** also explains the more open assembly morphology observed by TEM compared to PdNP-**1** or AuNP-**1**.^40,47^

## Pathway-Selective Assembly from Binary Mixtures

**Figure 3:**
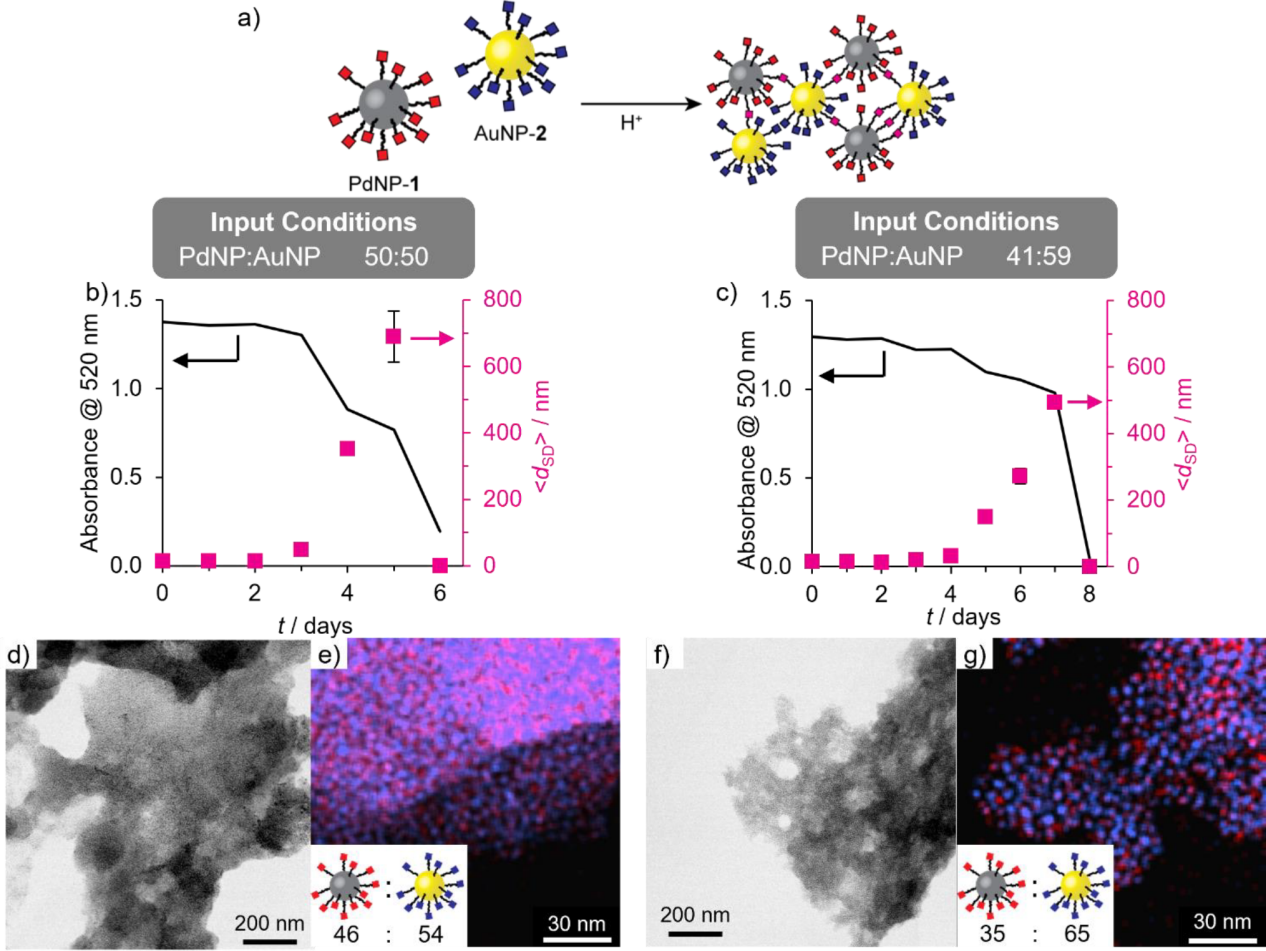
(a) Co-assembly of nucleophilic PdNP-**1** and electrophilic AuNP-**2** from binary colloidal mixtures. (b,c) Variation in solvodynamic diameter (⟨*d*_SD_⟩, magenta symbols) as measured by DLS and absorbance at 520 nm (line) over time after addition of the acid catalyst required for dynamic covalent hydrazone exchange. (d,f) Representative dark field STEM micrographs and (e,g) EDX maps of heteromaterial aggregates (blue = Au; red = Pd). Inset: Heteromaterial aggregate composition expressed in terms of ratio of nanoparticles (PdNP:AuNP) determined by EDX mapping on three distinct sample regions (for full results see Tables S3 and S4, with accompanying HAADF images in Figures S21 and S22). Assembly conditions (concentrations in terms of molecular species) (b, d, e): [PdNP-**1**]_0_ = 0.105 mM, [AuNP-**2**]_0_ = 0.075 mM (i.e., PdNP:AuNP = 50:50); (c, f, g): [PdNP-**1**]_0_ = 0.075 mM, [AuNP-**2**]_0_ = 0.075 mM (i.e., PdNP:AuNP = 41:59). Both experiments: [CF_3_CO_2_H]_0_ = 20 mM, 9:1 v/v DMF/H_2_O, room temperature.

We reasoned that the chemospecificity of hydrazone exchange reactions, combined with kinetic regioselectivity revealed by our molecular-level studies of reactivity for nanoparticle-bound versus bulk-solution species,^42,43,48^ could be exploited to selectively trigger any one of three unary or binary assembly pathways starting from a colloidal mixture of two nanoparticle building blocks (Figure 1). Subjecting a binary colloidal solution of the complementary pair of nucleophilic PdNP-**1** and electrophilic AuNP-**2** to the conditions required for dynamic hydrazone exchange produced aggregates incorporating both nanoparticles, without the need for any additional components or stimuli (Figure 3a). Monitoring by DLS and absorption spectroscopy (Figure 3b, c) revealed a very similar kinetic profile to that for linker-driven assembly of PdNP-**1**; as should be expected, the slowest molecular-level reaction governs the rate of the binary assembly process.^42^ Clusters several hundred nanometers in size are formed during an initial growth phase, but remain in solution, before a rapid precipitation phase during which the nanoparticle concentration in solution drops to virtually zero over a short period of time. Once again, assembly did not occur in the absence of the essential acid catalyst (Figure S37). The homomaterial binary mixture of AuNP-**1** and AuNP-**2** produced very similar results in DLS and absorption spectroscopy measurements when subjected to the same conditions (Figure S23); the loss of all plasmonic material from solution indicating that both of the complementary nanoparticle building blocks were incorporated into the precipitating aggregates. Thus, the molecule-driven assembly pathway is shown to be independent of the nature of the underlying nanomaterial core.

The precipitates were collected and washed to remove any residual colloidally stable materials. Analysis by STEM and energy-dispersive X-ray spectroscopy (EDX) revealed extended aggregates of intimately mixed PdNPs and AuNPs with compositions that closely reflect the makeup of the starting nanoparticle mixture (Figures 3d–g)—a direct consequence of the chemospecific interparticle hydrazone links. A starting nanoparticle mixture that was equimolar in terms of nanoparticles produced an assembly composition of 46:54 PdNP:AuNP (Figure 3e, Table S3). Alternatively, starting from a mixture that was equimolar in terms of nanoparticle-bound hydrazones—corresponding to a particle ratio of 41:59 PdNP:AuNP—aggregates of particle composition 35:65 PdNP:AuNP were produced (Figure 3g, Table S4; see Table S2 for a summary of initial state and aggregate outcome compositions for all experiments).

**Figure 4:**
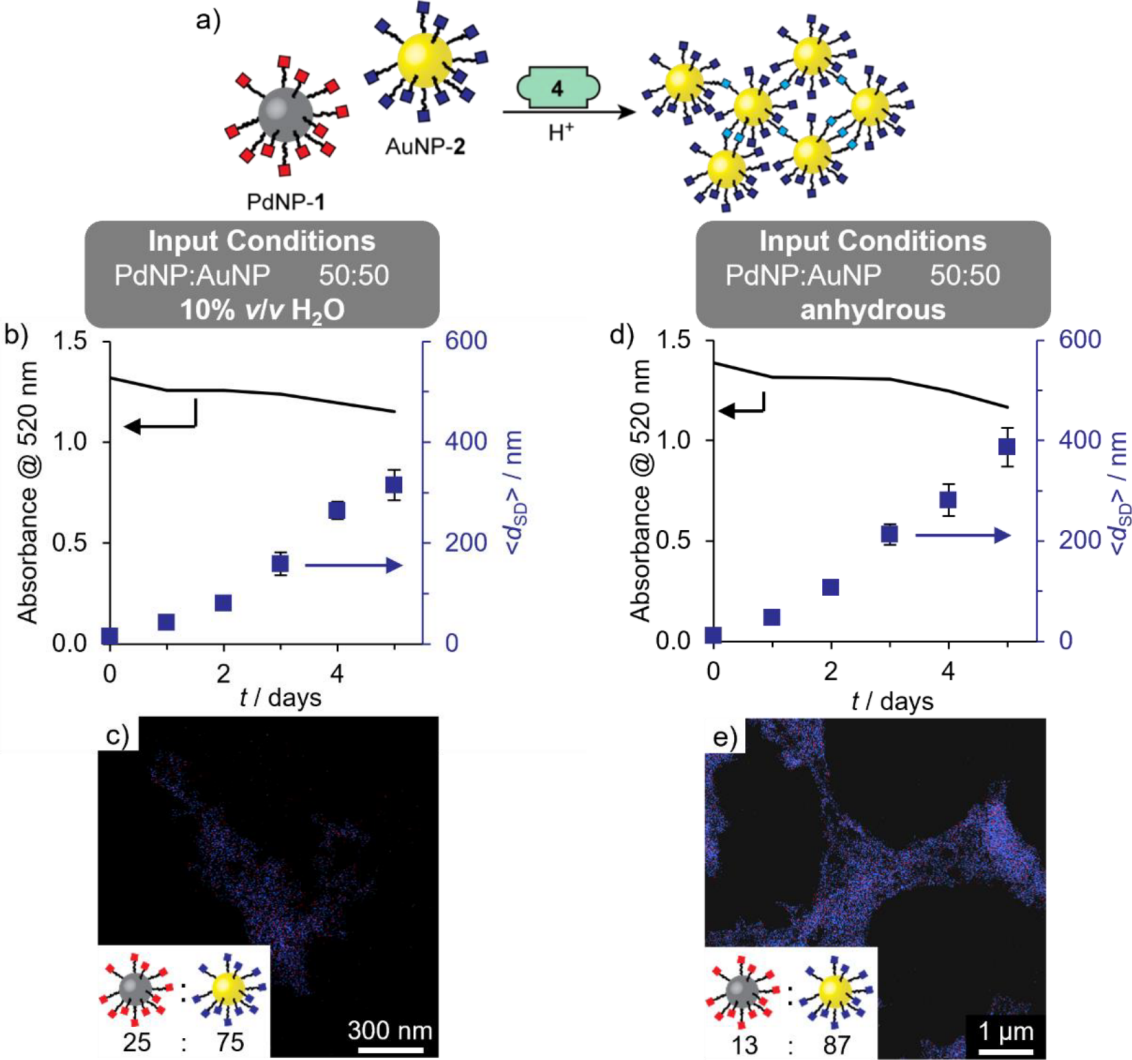
Selective assembly of AuNPs from a binary colloidal mixture of PdNP-**1** and AuNP-**2** under aqueous (b–c) and anhydrous (d–e) conditions. (b,d) Variation in solvodynamic diameter (⟨*d*_SD_⟩, blue symbols) as measured by DLS and absorbance at 520 nm (line) over time after addition of the acid catalyst required for dynamic covalent hydrazone exchange. (c,e) Representative EDX maps of gold-enriched aggregates (blue = Au; red = Pd). Inset: Heteromaterial aggregate composition expressed in terms of ratio of nanoparticles (PdNP:AuNP) determined by EDX mapping on three distinct sample regions (for full results see Tables S5 and S6, with accompanying HAADF images in Figures S24 and S25). Assembly conditions (concentrations in terms of molecular species): [PdNP-**1**]_0_ = 0.105 mM, [AuNP-**2**]_0_ = 0.075 mM (i.e., PdNP:AuNP = 50:50), [**4**]_0_ = 0.45 mM, [CF_3_CO_2_H]_0_ = 20 mM, room temperature, 9:1 *v*/*v* DMF/H_2_O (b–c) or anhydrous DMF (d–e).

Knowing that the nucleophilic linker-driven assembly of electrophilic AuNP-**2** occurs significantly faster than the coassembly process (which is governed by the slow hydrolysis kinetics of PdNP-**1**), we reasoned that adding a dihydrazide linker would favor formation of aggregates enriched in the nanoparticles functionalized with the electrophilic ligand (AuNP-**2**, Figure 4a). Treating the binary mixture of PdNP-**1** and AuNP-**2** (equimolar in terms of nanoparticles) with dihydrazide linker **4** (6 equiv with respect to nanoparticle-bound **2**) and CF_3_CO_2_H (Figure 4) led to assembly behavior quite unlike the coassembly experiment (compare Figures 3 and 4). Almost immediately, DLS measurements indicated formation of colloidally stable aggregates, reaching ⟨*d*_SD_⟩ = 315 nm at day 5. Because small palladium nanoparticles do not exhibit a strong LSPR absorbance, UV–vis absorption can be used to track the concentration of gold particles remaining in solution. At day 5, a small decrease in absorbance (Δ*A* = −0.2) was observed at λ_LSPR_ = 520 nm, indicating some precipitation of AuNP-**2**. Performing the same experiment with complementary gold-core nanoparticles (AuNP-**1** + AuNP-**2** + **4**) produced a very similar profile for the evolution of solvodynamic size distribution, and although both building blocks now contribute to the extinction at λ_LSPR_ = 520 nm, precisely the same absolute drop in absorbance values was observed in this experiment, consistent with the aggregation and precipitation of only electrophilic AuNP-**2** (Figure S31).

The insoluble material formed after 5 days was collected by centrifugation, and the recovered solid was washed to remove all traces of colloidally stable nanoparticles. Imaging by STEM again showed extended aggregates (Figures S24). EDX mapping of the heteromaterial assembly confirmed the selective incorporation of AuNPs (25:75 PdNP:AuNP, Figures 4c and Table S5).

Kinetic analysis of the underlying nanoparticle-bound molecular reactions previously revealed that dynamic covalent exchange of electrophilic AuNP-**2** with hydrazides proceeds predominantly through a direct transimination mechanism.^42^ Thus, we reasoned that water might be having a deleterious effect on selectivity by initiating exchange reactions on both AuNP-**2** and PdNP-**1**. Pleasingly, subjecting the same binary mixture of PdNP-**1** and AuNP-**2** to linker **4** in the absence of water increased the selectivity to produce aggregates even more strongly enriched in the gold particles (composition 13:87 PdNP:AuNP, Figure 4e and Table S6).

**Figure 5:**
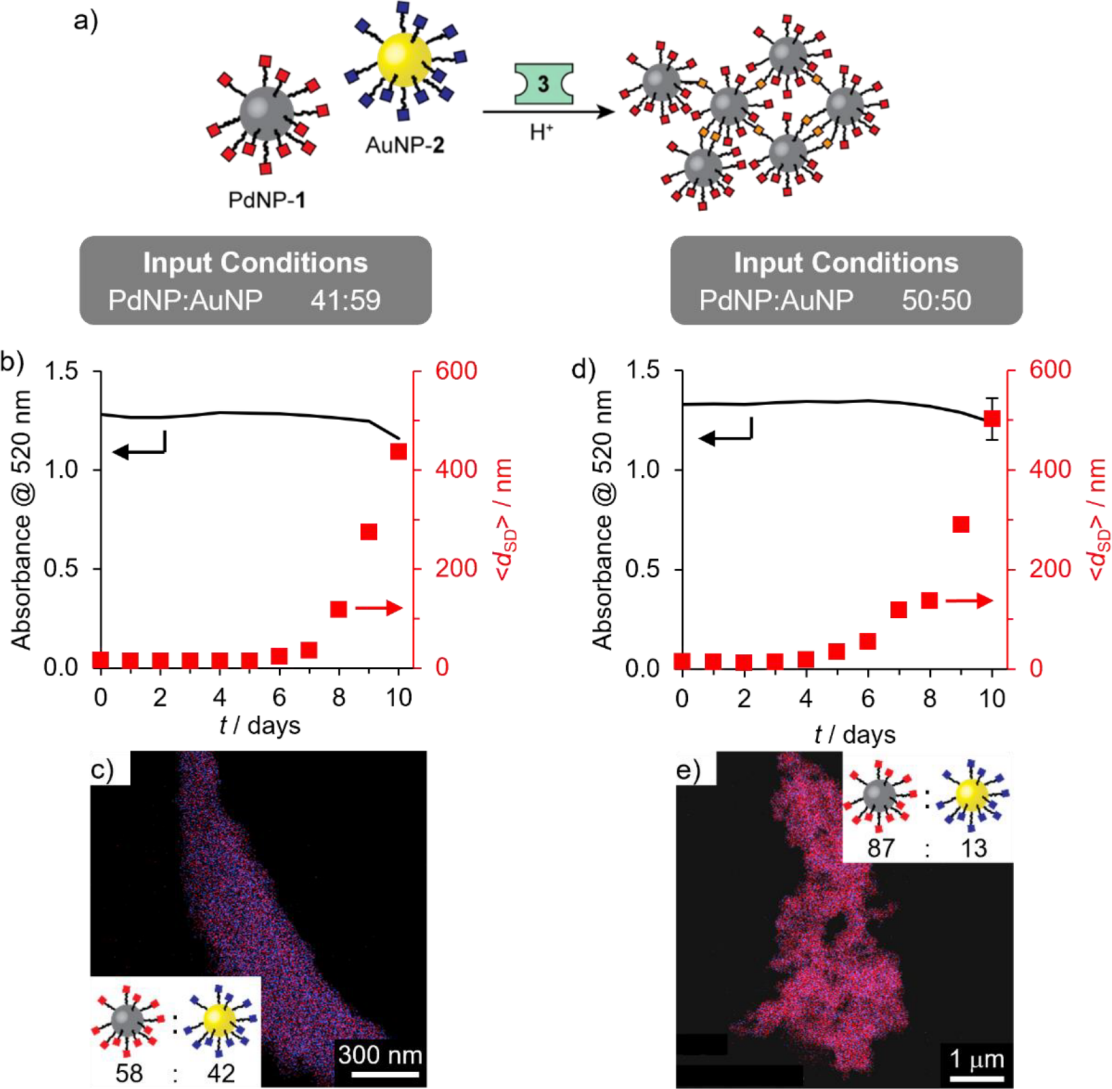
Selective linker-driven assembly of PdNPs from a binary colloidal mixture of PdNP-**1** and AuNP-**2**, equimolar in nanoparticle-bound hydrazones (b–c) or equimolar in nanoparticles (d–e). (b,d) Variation in solvodynamic diameter (⟨*d*_SD_⟩, red symbols) as measured by DLS and absorbance at 520 nm (line) over time after addition of the acid catalyst required for dynamic covalent hydrazone exchange. (c,e) Representative EDX maps of palladium-enriched aggregates (blue = Au; red = Pd). Inset: Heteromaterial aggregate composition expressed in terms of ratio of nanoparticles (PdNP:AuNP) determined by EDX mapping on three distinct sample regions (for full results see Tables S10 and S11, with accompanying HAADF images in Figures S32 and S33). Assembly conditions (concentrations in terms of molecular species) (b–c): [PdNP-**1**]_0_ = 0.075 mM, [AuNP-**2**]_0_ = 0.075 mM (i.e., PdNP:AuNP = 41:59), [**3**]_0_ = 0.45 mM; (d–e): [PdNP-**1**]_0_ = 0.105 mM, [AuNP-**2**]_0_ = 0.075 mM (i.e., PdNP:AuNP = 50:50), [**3**]_0_ = 0.45 mM; both experiments: [CF_3_CO_2_H]_0_ = 20 mM, room temperature, 9:1 v/v DMF/H_2_O.

The slower kinetics for dynamic covalent exchange of nucleophilic hydrazone nanoparticles with electrophilic molecular modifiers^42^ (Figure 2) portends a higher challenge for achieving selective assembly of PdNP-**1** using linker **3** (Figure 5a) over competing coassembly of PdNP-**1** with AuNP-**2**. In contrast to the assembly of electrophilic DCNPs with a nucleophilic linker, the dialdehyde linker cannot initiate reaction of nucleophilic DCNPs. The action of water is first required to produce a nanoparticle-bound nucleophile. We first treated a binary mixture of PdNP-**1** and AuNP-**2** that was equimolar in terms of surface-bound hydrazones (PdNP:AuNP 41:59) with 6 mol equiv of linker **3** and CF_3_CO_2_H. Reflecting the dominance of the slow hydrolysis–condensation pathway, no change was observed by DLS over the first 5 days (Figure 5b). From day 6 onward, colloidally stable aggregates were observed, growing in size to reach an average of ⟨*d*_SD_⟩ = 437 nm at day 10. The solution optical density changed very little over this period, consistent with a low yield of precipitate. Analysis of aggregates isolated after 10 days by EDX (Figure 5c and Table S10) revealed significant enrichment in PdNPs (PdNP:AuNP 58:42). Noting that the starting state in this experiment, which was equimolar in terms of NP-bound hydrazones, was biased in favor of AuNPs, we performed a similar experiment from a binary mixture equimolar in terms of particles (Figure 5d–e). With these input conditions, the same linker concentration corresponds to 4.3 mol equiv relative to the complementary nanoparticle-bound hydrazone PdNP-**1**. Monitoring by DLS and UV–vis revealed a very similar assembly profile (Figure 5d). However, the aggregates isolated after 10 days were even further enriched in Pd to 87:13 PdNP:AuNP (Figure 5e and Table S11)—representing remarkable selectivity given the relative rates of the underlying molecular-level processes that intrinsically favor incorporation of the electrophilic AuNP-bound hydrazones.

## A Predictive Framework for Molecule-Directed Selective Assembly of Nanoscale Building Blocks

**Figure 6:**
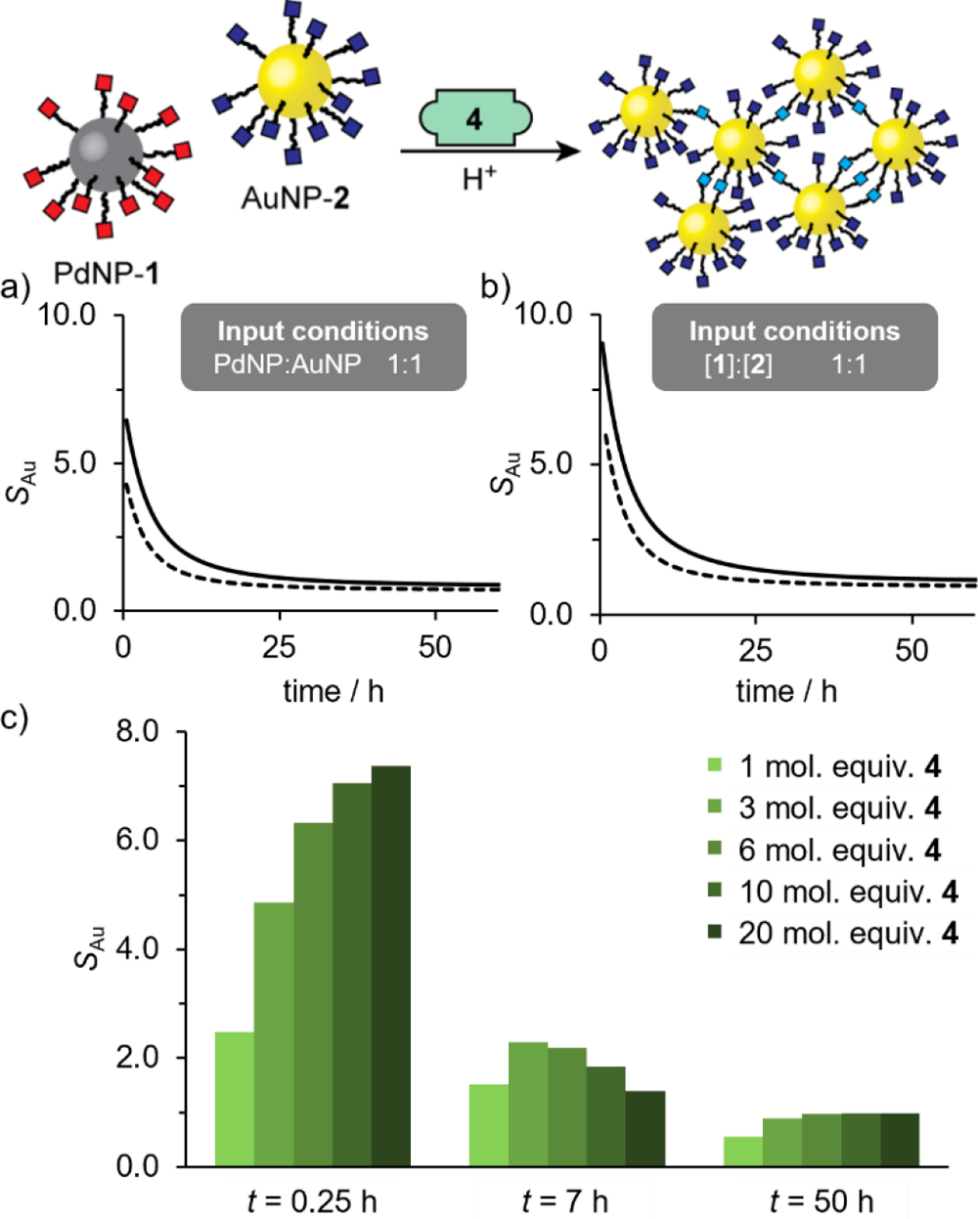
Summary of simulation results for nucleophilic linker-driven assembly from a binary mixture of nucleophilic PdNP-**1** and electrophilic AuNP-**2**. (a,b) Evolution of gold selectivity parameter *S*_Au_ (see Supporting Information for details) over time for input conditions that are equimolar in terms of nanoparticles (a) and equimolar in terms of nanoparticle-bound hydrazones (b) under anhydrous conditions (solid lines) or in the presence of water (dashed lines). For speciation profiles, see Figure S38. (c) Dependence of gold selectivity parameter *S*_Au_ on the concentration of nucleophilic linker **4** at different time points under equimolar nanoparticle-bound hydrazone input conditions in the presence of water (light green to dark green: 1 mol equiv, 3 mol equiv, 6 mol equiv, 10 mol equiv, 20 mol equiv of **4**). For evolution plot of S_Au_ against time, see Figure S39b.

To arrive at a more predictive understanding of the factors that have a significant influence on selectivity, we constructed kinetic models of the assembly processes (Supporting Information Section 6). Although several reaction pathways involving numerous nanoparticle-bound and bulk-solution substrates operate simultaneously during the multicomponent assembly experiments, even relatively simple kinetic models provided useful insight on the overall outcome. For assembly driven by nucleophilic linker **4**, we identified four reactions that should dominate during the initial stages (Scheme S2) and defined a selectivity parameter (*S*_Au_) as the concentration ratio for two key intermediates that are predicted to favor and disfavor selectivity (see Supporting Information for further discussion). The simulations reveal that homomaterial (which we define as “selective” in this context) and heteromaterial assembly pathways compete effectively and that selectivity is maximal at the start of the process (Figures 6 and S38). Consequently, isolating aggregates highly enriched in the AuNP building block entails a compromise between compositional selectivity and yield. In agreement with our experimental results, the simulations also predicted a significantly higher selectivity in favor of AuNP-enriched aggregates under anhydrous conditions (Figure 6a–b, compare solid and dashed lines).

An important consequence of employing selective intermolecular interactions to exert control over multifarious nanoscale building blocks is that the characteristic multivalency of each reactive nanoparticle introduces an intrinsic selectivity bias. A starting mixture that is equimolar in terms of nanoparticles (PdNP:AuNP = 50:50) appears to give a clear picture of overall selectivity, measured in terms of the mole fraction of gold nanoparticles incorporated in the final aggregate (e.g., Figure 4). However, the heteromaterial DCNP building blocks differ in core size and surface monolayer density so that, on a molecular level, the equimolar nanoparticle input conditions of Figure 4 are biased in favor of reaction of the PdNP-bound ligand **1** (molecular ratio PdNP-**1**:AuNP-**2** = 58:42, Table S2 Experiments LN1, LN2). To take account of the intrinsic bias in the input ratio of NPs when assessing selectivity, we defined the enrichment factor (*E*.*F*.) according to eq 1, where *F*_Au(input)_ and *F*_Au(aggregate)_ are the mole fractions of AuNPs in the initial mixture and final aggregates, respectively. This parameter allows comparison of selectivities between experiments with differing input compositions and between nanoscale building blocks of differing reactive site densities (Figure 8 and Table S2).
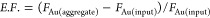
1

Our simulations confirmed the intuitive expectation that an input that is equimolar in terms of molecular reactants could exhibit even higher selectivity in favor of gold–gold homomaterial linkages (compare Figure 6a and 6b). We verified this prediction experimentally: starting from a mixture that is 50:50 PdNP-**1**:AuNP-**2** (i.e., a particle ratio PdNP:AuNP 41:59, Experiments LN3, LN4), aggregates formed after 5 days were even more highly enriched in gold, reaching 4:96 PdNP:AuNP under anhydrous assembly conditions (Figure S26). Comparing the *E*.*F*. for each experiment (Figure 8), it is revealed that the selectivity for AuNP incorporation from the equimolar ligand mixture is higher than that from the equimolar nanoparticle mixture (Figure 8, LN3 vs LN1). However, in the absence of water, the opposite effect is observed: although the equimolar ligand input conditions give the highest proportion of AuNPs in the final aggregate composition (Figure 8 LN4, PdNP:AuNP = 4:96, *E*.*F*. = 0.62), an equimolar particles input mixture produces a higher enrichment factor (Figure 8 LN2, PdNP:AuNP = 13:87, *E*.*F*. = 0.73), despite being biased against reaction of AuNP-bound ligands. The simulation results suggest that this observation is a consequence of the erosion in selectivity as the system evolves (Figure 6a–b). Thus, the enrichment depends on the time point of observation. Because the selectivity declines at different rates under each set of conditions, the enrichment factor observed is a complex function of all parameters. Furthermore, this analysis ignores the influence of kinetically controlled precipitation processes in generating the final aggregates that are analyzed in the real-world experiments.

Irrespective of the input ratio, the simulations also reveal that the optimum linker concentration depends on the time point at which aggregate selectivity is probed. At short time points, increasing linker concentration improves selectivity (Figure 6c, *t* = 0.25 h). The same trend is observed at long time points although sensitivity to the amount of linker used is much reduced (Figure 6c, *t* = 50 h). Yet, linker concentration also increases the rate at which selectivity is eroded (Figure S39b). Consequently, at intermediate time points, intermediate concentrations of linker can be optimal (Figure 6, *t* = 7 h). We observed this phenomenon experimentally, where the highest aggregate enrichment factor measured at day 6 was achieved when using only 3 mol equiv of linker **4** (Experiment LN5, Figures 8, S29–S30 and Table S9).

**Figure 7:**
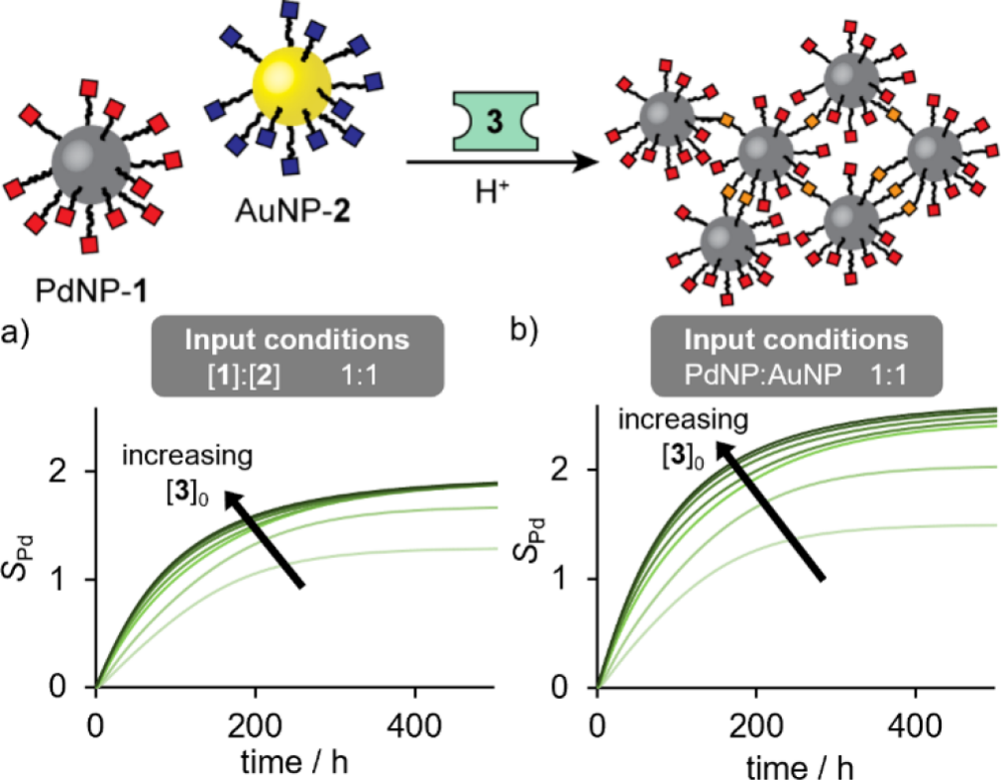
Summary of simulation results for electrophilic linker-driven assembly from a binary mixture of nucleophilic PdNP-**1** and electrophilic AuNP-**2**. Evolution of selectivity parameter *S*_Pd_ (describing ratio of interparticle linkages favoring incorporation of PdNPs over AuNPs in aggregates, see Supporting Information for details) over time for input conditions that are equimolar in terms of nanoparticle-bound hydrazones (a) or equimolar in terms of nanoparticles (b) over a range of concentrations of electrophilic linker **3** (light green to dark green: [**3**]_0_ = 0.075 mM, 0.15 mM, 0.45 mM, 0.75 mM, 1.50 mM, 2.25 mM, 3.00 mM). (a) [**1**]_0_ = [**2**]_0_ = 0.075 mM; (b) [**1**]_0_ = 0.105 mM, [**2**]_0_ = 0.075 mM.

The differences in underlying molecular mechanism for assembly driven by electrophilic linker **3** compared to nucleophilic linker **4** (vide supra) means that the influence of both water and the linker concentration are quite different in each process. Simulations of a simplified kinetic model (see Supporting InformationScheme S3 and accompanying discussion) reveal that selectivity for nucleophilic palladium-core DCNPs in the presence of an electrophilic linker (*S*_Pd_) reaches a maximal steady state at long time periods (Figure 7)—quite unlike the behavior predicted for the assembly driven by a nucleophilic linker (vide supra). Reflecting the kinetics of the critical nanoparticle-bound hydrolysis reactions, the simulations predict a slow process that generates a small percentage of interparticle linkages (Figures S40–S41), consistent with the experimental observation that small quantities of aggregates were produced over relatively long time scales.

**Figure 8:**
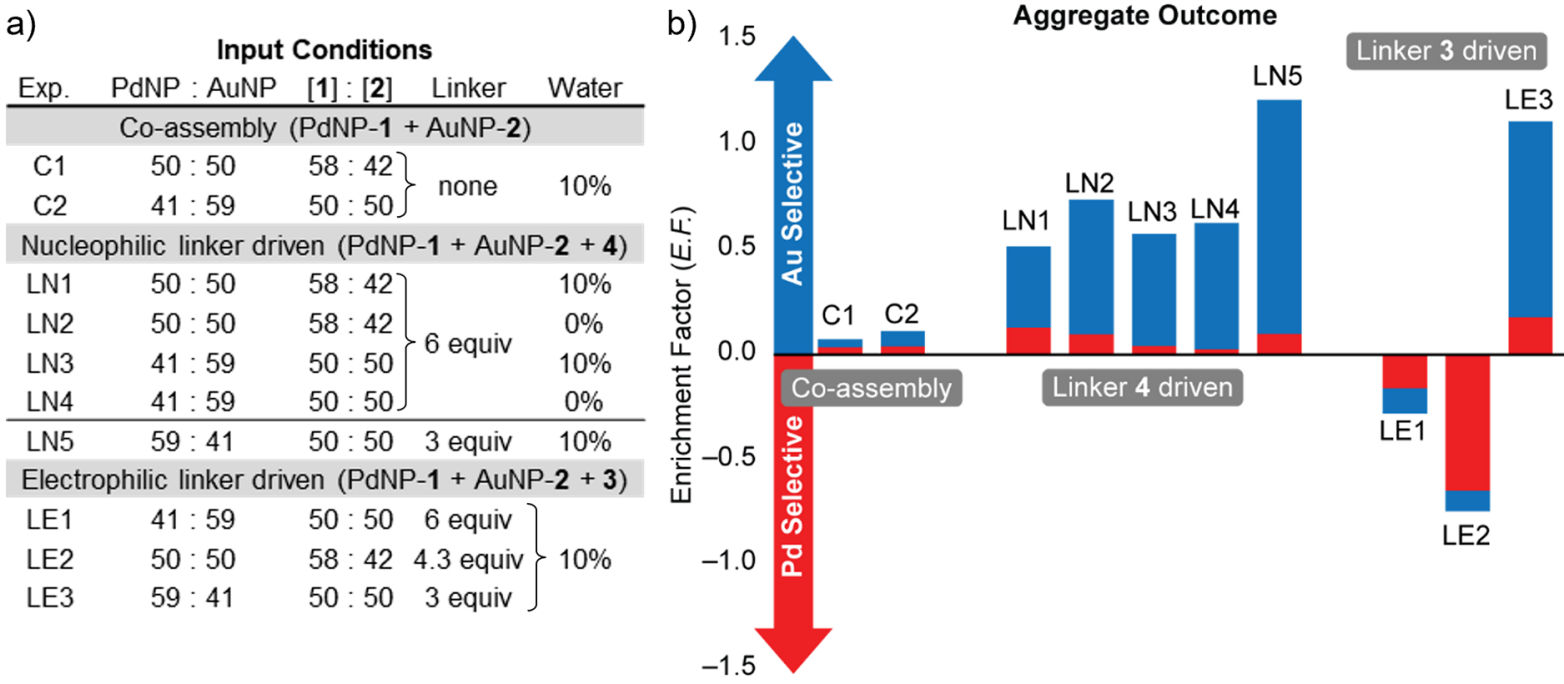
Summary of heteromaterial assembly experiments. (a) Input conditions expressed in terms of both nanoscale (PdNP:AuNP) and molecular scale ([**1**]:[**2**]) stoichiometries. Molar equivalents of linker are given relative to the corresponding complementary nanoparticle-bound hydrazone (i.e., **3** relative to PdNP-**1**; **4** relative to AuNP-**2**). (b) Assembly outcomes expressed in terms of mole fraction of each nanoparticle (relative proportions of bars: blue = *F*_AuNP(aggregate)_; red = *F*_PdNP(aggregate)_) and enrichment factor (total height of bars, *E*.*F*. = [*F*_Au(aggregate)_ – *F*_Au(input)_]/*F*_Au(input)_). For a full summary of numerical results, see Table S2.

In this reaction network, because the products of hydrolysis reactions are involved in both selective and nonselective pathways, the parameter with the strongest potential to positively influence aggregate selectivity is the linker concentration. Higher stoichiometric ratios of linker should increase selectivity (Figure 7), but at the expense of reduced aggregate yield (Figure S41). This suggests that moderate molar excesses of linker are optimal. At low linker concentrations, the simulations predict values of *S*_Pd_ < 1, corresponding to a higher number of heteromaterial than homomaterial interparticle links, suggesting coassembly of both particles. An experiment employing only 3 mol equiv of **3** resulted in a similar assembly profile to the others, but surprisingly generated aggregates where the expected selectivity was entirely reversed, being enriched in Au (Experiment LE3, Figures 8, S34–S35 and Table S12). The origins of this reversal in selectivity can be understood by examining the speciation profiles for assembly in the presence of a low concentration of electrophilic linker (Figure S42a–b). Nucleophilic Pd-bound hydrazides generated on hydrolysis of PdNP-**1** are inefficiently captured by the low concentration of linker **3**. This is exacerbated by the preferential solution-phase condensation of **3** with the hydrazide released from AuNP-**2**, which simultaneously consumes **3** and drives formation of AuNP-bound aldehydes. Thus, any PdNP-bound nucleophiles will be rapidly capped by reaction with the relatively high concentration of reactive AuNP-bound aldehydes (reflected in the rapid rise in interparticle links at low linker concentrations, Figure S41). Each PdNP core can therefore become surrounded by several AuNPs, rapidly leading to Au-rich assemblies that quickly grow to be colloidally unstable, kinetically trapping Au-enriched aggregates by precipitation. This contrasts with the situation when no linker is present at all (Figure S42c–d), where nucleophilic and electrophilic DCNPs react at roughly similar rates to give heteromaterial assemblies that grow gradually, giving compositions that reflect the input stoichiometry irrespective of the point at which precipitation occurs.

In the experiments comparing starting states equimolar in ligands versus equimolar in nanoparticles (Experiments LE1 and LE2, respectively, Figures 5 and 8), the latter corresponded to a higher concentration of Pd-bound reactive sites, such that the same concentration of linker corresponds to a lower stoichiometric excess with respect to the target PdNP-bound hydrazones (4.3 molar equiv versus 6 molar equiv). Under these input conditions, the simulations predicted that the optimal balance between aggregate yield and selectivity can be struck, giving higher values for both *S*_Pd_ (Figure 7) and yield of interparticle linkages (Figure S41). This is directly reflected in the experimental results where the equimolar nanoparticle starting point (Experiment LE2, Figure 5d–e, Figure 8) produced both the highest absolute selectivity and enrichment factor in favor of incorporating the PdNP building block, despite employing only a moderate stoichiometric excess of ligand.

## Conclusions

We have demonstrated that complementary nucleophilic and electrophilic hydrazone-based dynamic covalent nanoparticles (DCNPs) can be directed down different assembly pathways by applying specific molecular-level instructions—in much the same way as complementary reactivity is exploited in synthetic molecular chemistry. Applied to binary mixtures of two DCNP building blocks, coassembly gives intimately mixed heteromaterial aggregates; alternatively, selective reaction with the appropriate complementary molecular linker produces aggregates enriched in either one of the two building blocks. The control over assembly outcome from the same starting mixture of building blocks is rooted in the molecular chemospecificity (aldehydes only react with hydrazides) and kinetic regioselectivity (differential rates for reaction at surface-bound versus solution-phase substrates) of the dynamic covalent exchange reactions. The chemospecific directional hydrazone links ensure that binary aggregates are intimately mixed—building block phase separation is not possible. Meanwhile, kinetic regioselectivity for molecule–nanoparticle over nanoparticle–nanoparticle reactions is the basis for selective reaction between solution-phase linkers and reactive nanoparticles to produce aggregates highly enriched either one of the core materials starting from the same mixture of building blocks.

Selectivity levels are sensitive to the several system parameters. The “pseudomolecular” characteristics of DCNPs have previously allowed us to establish a molecular-level kinetic and mechanistic understanding of the nanoparticle-bound reactions.^42,43,48^ This window on the molecular processes that drive nanoscale assembly informed the construction of kinetic models, through which simulations could guide optimization of reaction conditions to maximize selectivity for each of the three assembly pathways. Despite the system complexity that combines solution phase, colloidal surface-confined and solid-state chemical reactions with heterogeneous physical processes including aggregation and precipitation, our simple kinetic models provide a remarkable level of insight on the link between molecular-level events and nanoscale assembly outcome. This establishes that, given sufficient information regarding the underlying molecular reactions and interactions, it is possible to achieve a predictive understanding of nanoscale construction processes.

These studies highlight important implications of directing nanoscale entities using interactions on molecular length scales. Our understanding of chemical events is expressed in terms of molecular concentrations, whereas the material-level outcomes are characterized in terms of nanoscale components. Consequently, a systems-level analysis must link the molecular stoichiometry and nanoscale stoichiometry. Such relationships are unique to each distinct nanoparticle building block and may even vary from batch to batch for ostensibly the same nanoscale components. While molecular concentrations and stoichiometries control the colloidal assembly pathway at short time scales, aggregation and precipitation are intrinsically nanoscale in nature and become increasingly important as the system evolves. It is therefore remarkable that relatively crude kinetic models are able to reproduce the experimental outcomes so closely. Integrated models that incorporate heterogeneous physical processes alongside homogeneous, surface-confined and solid-state molecular events,^49^ can be expected to be even more powerful and will be required to fully elevate the predictability of hierarchical nanoscale assembly to match that of molecular construction strategies.

In several settings, the combination of multiple orthogonal and tunable thermodynamically controlled reactions is emerging as a powerful strategy for constructing a variety of adaptive chemical structures, networks, and materials of increasingly remarkable complexity.^50−52^ Furthermore, our ability to exploit and understand the effects of confinement on chemical reactions and interactions extends the reach of rational synthetic chemical concepts beyond molecular length scales and bulk solution environments.^53^ Controlled by molecular interactions taking place at the periphery of an abiotic molecular monolayer, the principles developed here are independent of the underlying nanomaterial and therefore generalizable to virtually any monolayer-stabilized nanoparticle system. Consequently, by developing a small suite of selective—potentially orthogonal—and addressable dynamic covalent nanoparticle building blocks,^38,40−43,54,55^ one can envision compiling a versatile toolkit of modular nanoscale synthons that mirrors the long-established reactive building blocks of molecular synthesis. This would set the stage for a new era of multicomponent composites that exhibit responsive collective and emergent properties dependent on building blocks from several length scales.^56^

## Supporting Information Available

General experimental procedures; detailed synthetic methods and characterization for molecular compounds and nanoparticles; experimental procedures for nanoparticle assembly experiments; supplementary electron microscopy images; results for supplementary assembly experiments and controls; description and extended discussion of kinetic models and simulation results. (PDF)
