# Supplementary material for: Constitutionally Selective Dynamic Covalent Nanoparticle Assembly
Source: J Am Chem Soc. 2022 Jul 28;144(31):14310–21. doi: 10.1021/jacs.2c05446 (PMC9376925; doi:10.1021/jacs.2c05446)
Supplement: Supplementary file 1 — ja2c05446_si_001.pdf [file ja2c05446_si_001.pdf]

Supporting Information

Constitutionally selective dynamic covalent nanoparticle assembly

*Nicolas Marro, Rongtian Suo, Aaron B. Naden and Euan R. Kay**

EaStCHEM School of Chemistry, University of St Andrews, North Haugh, St Andrews, KY16 9ST, UK.

* Email: ek28@st-andrews.ac.uk

Twitter: @euanrkay

1. General experimental procedures S2

2. Synthesis of molecular pro-ligands S3

3. Synthesis of nanoparticle building blocks S4

'Nucleophilic' nanoparticles AuNP-**1** S4
'Nucleophilic' nanoparticles PdNP-**1** S4
'Electrophilic' nanoparticles AuNP-**2** S7

4. Linker-driven assembly of individual DCNP building blocks S9

Nucleophilic PdNP-**1** with dialdehyde linker **3** S10
Nucleophilic AuNP-**1** with dialdehyde linker **3** S10
Electrophilic AuNP-**2** with dihydrazide linker **4** S11
Nucleophilic PdNP-**1** – control experiments 1 and 2 S12
Nucleophilic AuNP-**1** – control experiments 3 and 4 S13
Electrophilic AuNP-**2** – control experiments 5 and 6 S14

5. Complementary and selective heteromaterial assembly from binary DCNP mixtures S15

Experiments C1, C2: Co-assembly S18
Complementary co-assembly from a homomaterial mixture S19
Experiments LN1–LN5: Nucleophilic linker selected assembly S19
Experiments LE1–LE3: Electrophilic linker selected assembly S25
Control experiments S28

6. Kinetic models of selective assembly processes S29

Selective assembly of electrophilic DCNPs using a nucleophilic linker S29
Selective assembly of nucleophilic DCNPs using an electrophilic linker S32
Parameter estimation for kinetic models S37

7. References and notes S41

1. General experimental procedures

Unless stated otherwise, all reagents were purchased from commercial sources and used without further purification. Dry *N*,*N*-dimethylformamide was purchased from Acros Organics. Prior to use, traces of dimethylamine were removed by evaporation under vacuum with gentle heating, followed by cooling to room temperature under vacuum then storage under a nitrogen atmosphere. All other dry solvents were obtained by means of a MBRAUN MB SPS-800TM solvent purification system, where solvents were passed through filter columns and dispensed under an argon atmosphere. Flash column chromatography was performed using Geduran® Si60 (40–63 µm, Merck, Germany) as stationary phase. Thin-layer chromatography (TLC) was performed on pre-coated silica gel plates (0.25 mm thick, 60F254, Merck, Germany) and observed under UV light (λ_{max} 254 nm) or visualized by staining with acidic ceric ammonium molybdate solution, followed by heating.

Scanning transmission electron microscopy (STEM) was performed on an FEI Titan Themis operated at 200 kV and equipped with a CEOS DCOR probe corrector and a SuperX energy dispersive X-ray spectrometer (EDX). High angle annular dark field (HAADF) images were acquired with a probe convergence angle of 21.2 mrad and inner/outer collection angles of 56.3 and 200 mrad, respectively. Transmission electron microscopy (TEM) was also performed using a JEM 2010 microscope. High-resolution scanning electron microscopy (SEM) was performed using a Scios dualbeam microscope. Samples for electron microscopy were prepared by deposition of one drop of nanoparticle suspension on holey carbon films supported on a 300 mesh Cu grid (Agar Scientific®). Nanoparticle diameters were measured automatically using the software ImageJ. The images were first converted to black and white images using the “Threshold” function. The area of each nanoparticle was measured using the “Analyze particles” function. Particles on edges were excluded. UV-vis absorption spectroscopy was performed using a quartz cuvette (10 mm path length) on a Thermo Scientific Evolution 220 UV-Visible Spectrophotometer or an Agilent Technologies Cary 100 Series UV-vis Bio Spectrophotometer. Dynamic light scattering (DLS) measurements were performed using a glass cuvette (10 mm path length) on a Malvern Zetasizer µV or a Zetasizer Nano-ZS instrument. Each data point is the average of three independent measurements made in series. For each sample, three independent measurements were taken in series, and the results averaged. In turn, each measurement is the average of 10–17 sequential scans. The solvodynamic sizes are reported as the mean size for distributions expressed as % volume of particulate material. Size distributions were calculated by the instrument from the recorded intensity data. Equations reported in the literature were used to estimate appropriate values for viscosity, refractive index, and dielectric constant of 9:1 *v*/*v* DMF/D_2O from the reported values for the neat solvents at 25 °C.[1-2 3] ^{1}H, ^{13}C, ^{19}F NMR spectra were recorded on Bruker Avance 300, Avance II 400 and Avance III 400, 500 and 700 MHz instruments, at a constant temperature of 25 °C. ^{1}H Chemical shifts are reported in parts per million (ppm) from high to low field and referenced to the literature values for chemical shifts of residual non-deuterated solvent, with respect to tetramethylsilane.[4] ^{19}F Chemical shifts are referenced to $CFCl_3$ (0.00 ppm) as external standard. Standard abbreviations indicating multiplicity are used as follows: bs (broad singlet), d (doublet), m (multiplet), q (quartet), s (singlet), t (triplet), J (coupling constant). All spectra were analyzed using MestReNova (Version 10.0.2). All melting points were determined using a Stuart SMP30 Melting Point Apparatus and are reported uncorrected. Freeze drying was achieved using a Christ Alpha 1–2 LD Freeze dryer (Martin Christ GmbH, Osterode am Harz, Germany) at −54 °C, 0.15 mbar vacuum for ca. 15–20 h until complete dryness. Thermal gravimetric analysis (TGA) and differential thermal analysis (DTA) were performed on a Staton Redcroft STA-780 simultaneous TG-DTA instrument connected to a Rheometric Scientific STA SID System Interface. Pure and dried nanoparticle samples (ca. 3-5 mg) were weighed into a blanked alumina crucible. The sample was then heated under a stream of air at a ramp rate of 10 ºC min^{-1} from room temperature to 900 ºC.

2. Synthesis of molecular pro-ligands

Disulfide pro-ligands **1**$_2$ and **2**$_2$ were prepared as previously described.[5]

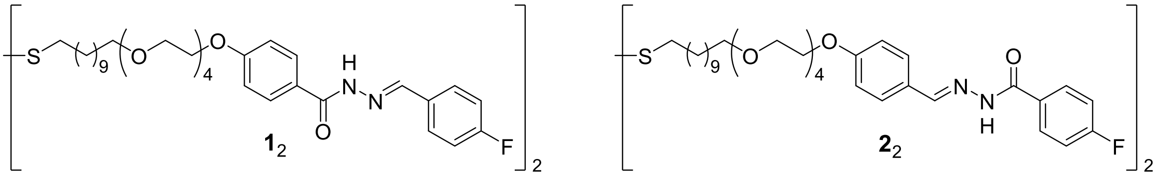

Scheme S1. Molecular structures of nucleophilic (**1**$_2$) and electrophilic (**2**$_2$) disulfide pro-ligands used for the synthesis of AuNP-**1**, PdNP-**1**, and AuNP-**2**.

*(E)-N'-(4-Fluorobenzylidene)-4-((23-mercapto-3,6,9,12-tetraoxatricosyl)oxy) benzohydrazide (**1**H)*

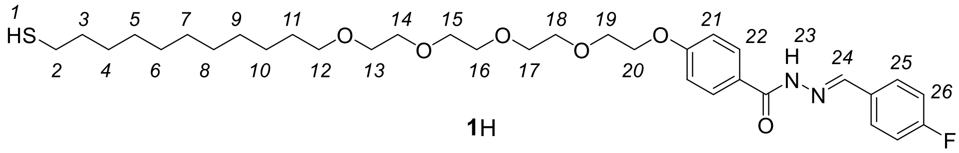

Disulfide **1**$_2$ (0.51 g, 0.41 mmol), 1,4-dithiothreitol (0.38 g, 2.46 mmol) and CH_3CO_2Na (0.10 g, 1.2 mmol) were dissolved in THF/MeOH/H_2O (3:1:0.1 *v*/*v*/*v*, 18 mL). The reaction mixture was stirred at room temperature for 15 h, then poured into CH_2Cl_2 (60 mL). The organic phase was washed with saturated aqueous $NaHCO_3$ (5 × 50 mL), saturated brine (2 × 50 mL), then dried over $MgSO_4$, filtered and evaporated under reduced pressure to afford **1**H as a white wax. Obtained 0.42 g, yield 81%.

^{1}H NMR (500 MHz, $CDCl_3$): δ 1.23–1.45 (m, 15H, H_1 and H_4–H_{10}), 1.55–1.67 (m, 4H, H_3 and H_{11}), 2.68 (dt, *J* = 7.2 Hz, 2H, H_2), 3.44 (t, *J* = 6.8 Hz, 2H, H_{12}), 3.58–3.77 (m, 12H, H_{13}–H_{18}), 3.88–3.93 (m, 2H, H_{19}), 4.17–4.21 (m, 2H, H_{20}), 6.95 (d, *J* = 7.2 Hz, 2H, H_{21}), 7.11 (t, *J* = 8.0 Hz, 2H, H_{26}), 7.54–7.73 (m, 2H, H_{25}), 7.77–7.94 (m, 2H, H_{22}), 8.18 (s, 1H, H_{24}), 9.29 (bs, 1H, H_{23}) ppm.

^{13}C{^{1}H} NMR (126 MHz, $CDCl_3$): δ 26.3, 28.7, 29.5, 29.6, 29.8, 29.9, 29.9, 30.0, 32.4, 39.5, 67.8, 69.5, 70.1, 70.5, 70.7, 70.8, 70.9, 71.2, 71.8, 114.5, 115.9 (d, *J* = 21 Hz), 125.7, 129.6 (d, *J* = 8.0 Hz), 130.1, 130.9 (d, *J* = 1.8 Hz), 147.4, 162.1, 164.1 (d, *J* = 248 Hz), 164.2 ppm.

^{19}F{^{1}H} NMR (376.4 MHz, $CDCl_3$): δ –110.31 (s) ppm.

HRMS (ES$^+$) calculated *m/z* for $C_{33}H_{50}FN_2O_6S$ [M+H]$^+$ 621.3368, found 621.3364

3. Synthesis of nanoparticle building blocks

'Nucleophilic' nanoparticles AuNP-1

Gold core AuNP-**1** were prepared, isolated and purified as previously described.[5] Structural and compositional details (Table S1) were assessed by electron microscopy (Figure S1) and thermal gravimetric analysis (Figure S2).

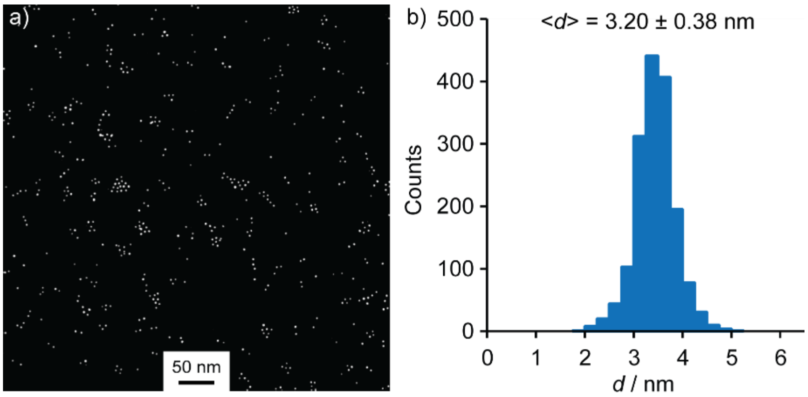

Figure S1. Representative STEM micrograph (scale bar 50 nm) for AuNP-**1**. Size distribution (determined for > 1600 particle measurements): <*d*> = 3.20 ± 0.38 nm (12% dispersity).

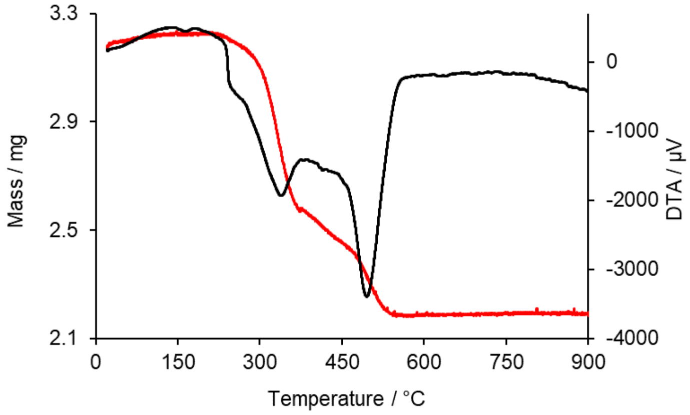

Figure S2. TGA (red curve) and DTA (black curve) plots for AuNP-**1** (3.17 mg). The sample was heated under air at a ramp rate of 10 °C min^{-1} over the range 20–900 °C.

'Nucleophilic' nanoparticles PdNP-1

Two batches of palladium core PdNP-**1** were prepared using slightly different procedures, giving nanoparticles with high (PdNP-**1**(1)) and low (PdNP-**1**(2)) monolayer densities

*PdNP-**1**(1)*

Using a modified version of the synthetic procedure originally developed by Stucky and co-workers,[6] palladium(II) acetylacetonate (0.25 g, 0.50 mmol) was weighed into a 100 mL round bottom flask and dissolved in $CHCl_3$ (40 mL). Hexanethiol (0.151 mL, 0.125 g, 1.06 mmol) was added and the reaction mixture heated to 55 °C. *tert*-Butylamine borane complex (0.44 g, 5.05 mmol) was added and the reaction was held at 55 °C for 1 h before being allowed to stir at room temperature for 5 h. The solution was transferred to a 250 mL flask to which MeOH/MeCN (4:1 *v*/*v*, 140 mL) was added before standing in the freezer overnight. The supernatant was removed, the residue dissolved in the minimal volume of CH_2Cl_2 and transferred to a vial, then the solvent was removed under a stream of compressed air. The residue was dispersed in MeOH with sonication, then subjected to centrifugation (1312 ×g rcf, 20 min). The supernatant was removed, and the pellet redispersed in fresh solvent. The nanoparticles were washed repeatedly in this manner with MeOH (×3), then MeCN (×2), after which no further impurities were observed in the supernatant by TLC. The black residue was then dried under vacuum overnight. The hexanethiyl-stabilized nanoparticles (65 mg) were then

suspended in CH_2Cl_2/DMF (4:1 *v*/*v*, 8 mL) and the mixture sonicated to ensure good dispersion of the nanoparticles. Thiol **1**H (0.750 g, 1.38 mmol) was added and the mixture was stirred at room temperature in the dark. After 2 days, despite being still darkly coloured, the solution appeared cloudy with some solid precipitate at the bottom of the flask. Complete precipitation was achieved by addition of EtOH (20 mL), followed by centrifugation (1312 ×g rcf, 20 min). The supernatant was removed and discarded. In the same manner, the nanoparticles were subjected to a further two cycles of washing in Et_2O/EtOH (1:1 *v*/*v*, 15 mL). After the final wash, traces of volatiles were removed under gentle air flow. Analysis by ^{1}H NMR spectroscopy following oxidative ligand desorption of a small sample indicated residual surface-bound hexanethiyl ligands. The nanoparticle solid was therefore suspended in DMF (8 mL) and the mixture sonicated. A further portion of thiol **1**H (1.00 g, 1.84 mmol) was added and the solution was stirred at room temperature in the dark for a further 3 days. Nanoparticles were then precipitated upon addition of 6:1 *v*/*v* Et_2O/EtOH (25 ml), followed by centrifugation (1312 ×g rcf, 20 min). The colourless supernatant was carefully discharged, before the black solid obtained was washed using the following procedure: nanoparticles were dispersed in Et_2O/EtOH/CH_2Cl_2 (3:1:0.1 *v*/*v*, 15 mL), sonicated for 15 min, then centrifuged (1312 ×g rcf, 20 min). This operation was repeated a further 3 times. At this stage, no unbound molecular species were detected in the supernatant by TLC or NMR analysis. Traces of volatile solvents were removed from the purified residue under a stream of compressed air, to provide PdNP-**1**(1) (58 mg).

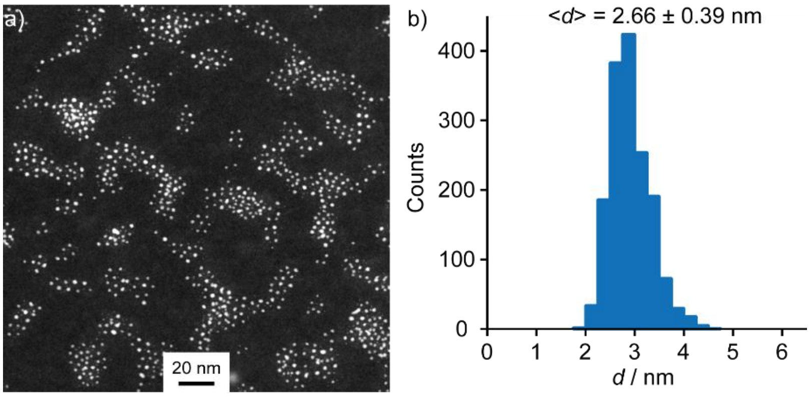

Figure S3. Representative STEM micrograph (scale bar 20 nm) for PdNP-**1**(1). Size distribution (determined for > 1600 particle measurements): <*d*> = 2.66 ± 0.39 nm (15% dispersity).

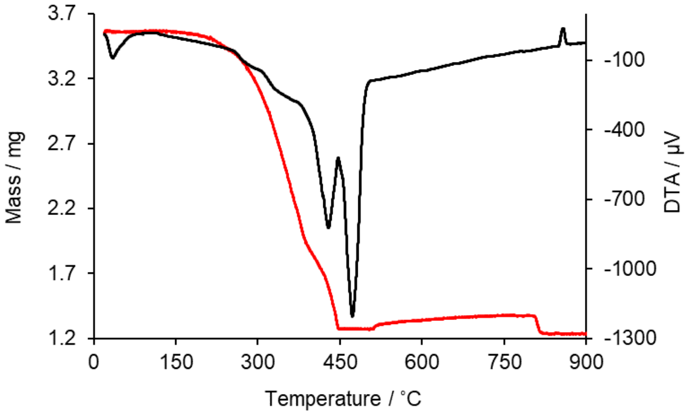

Figure S4. TGA (red curve) and DTA (black curve) plots for AuNP-**1** (3.55 mg). The sample was heated under air at a ramp rate of 10 °C min^{-1} over the range 20–900 °C.

*PdNP-**1**(2)*

Using a modified version of a two-step synthetic procedure,[7] a solution of $PdCl_2$ (0.250 g, 1.41 mmol) in H_2O (25 ml) and HCl (5 ml) was extracted with a solution of tetraoctylammonium bromide (1.54 g, 2.82 mmol) in N_2 purged toluene (150 mL). To the resulting orange organic solution, dioctylamine (8.52 mL, 28.2 mmol) was added. The mixture was vigorously stirred under N_2 for 1 h. After cooling the mixture to 0 °C, a solution of $NaBH_4$ (1.54 g, 2.48 mmol) in H_2O (5 mL) was added rapidly. After 1.5 h of stirring at 0 °C, the aqueous layer was removed. To the obtained nanoparticle solution, thiol

1H (1.53 g, 2.82 mmol) in DMF (5 mL) was added rapidly. The reaction mixture was stirred for 15 min at room temperature before complete nanoparticle precipitation occurred. The colourless supernatant was carefully discharged, then the black solid obtained was washed using the following procedure: nanoparticles were dispersed in EtOH/toluene (1:1 *v*/*v*, 12 mL), sonicated for 10 min, then centrifuged (1312 ×g rcf, 10 min, 4 °C). This operation was repeated a further two times. Traces of volatile solvents were removed from the purified residue under a stream of compressed air. The solid obtained was dissolved in DMF (10 mL) and thiol **1**H (0.780 g, 1.41 mmol) was added. The solution was stirred at room temperature for 3 h, before nanoparticles were precipitated upon addition of Et_2O/EtOH (6:1 *v*/*v*, 50 ml). The colourless supernatant was carefully discharged, while the black solid obtained was washed using the following procedure: nanoparticles were dispersed in Et_2O/EtOH/CH_2Cl_2 (3:1:0.1 *v*/*v*, 10 mL), sonicated for 15 min, then centrifuged (1312 ×g rcf, 10 min, 4 °C). The operation was repeated a further three times. At this stage, no unbound molecular species were detected in the supernatant by TLC or NMR analysis. Traces of volatile solvents were removed from the purified residue under a stream of compressed air, to provide PdNP-**1**(2) (45 mg).

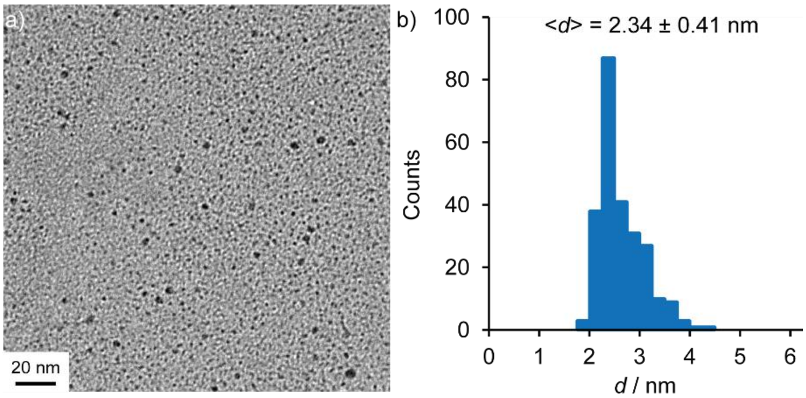

Figure S5. Representative TEM micrograph (scale bar 20 nm) for PdNP-**1**(2). Size distribution (determined for > 300 particle measurements): <*d*> = 2.34 ± 0.41 nm (17% dispersity).

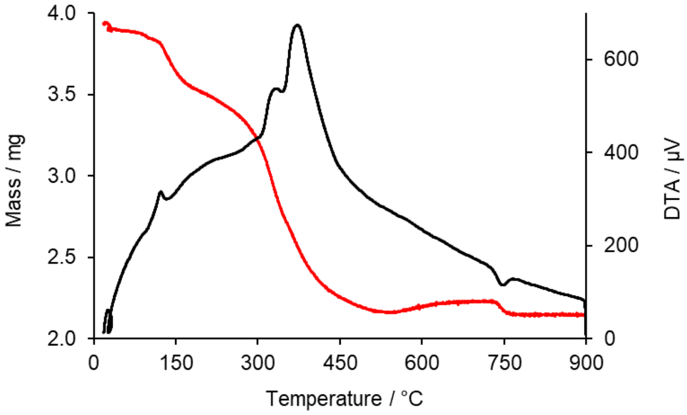

Figure S6. TGA (red curve) and DTA (black curve) plots for PdNP-**2**(2) (3.93 mg). The sample was heated under air at a ramp rate of 10 °C min^{-1} over the range 20–900 °C.

‘Electrophilic’ nanoparticles AuNP-2

Two batches of gold core AuNP-**2** were prepared, isolated and purified as previously described.[5] Structural and compositional details (Table S1) were assessed by electron microscopy (Figures S7, S9) and thermal gravimetric analysis (Figure S8, S10). Although differing in mean core size, each batch had a similar monolayer density.

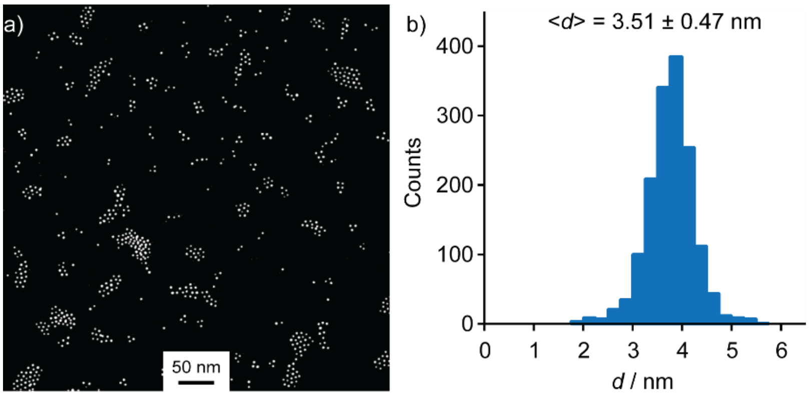

Figure S7. Representative STEM micrograph (scale bar 50 nm) for AuNP-**2**(1). Size distribution (determined for > 1550 particle measurements): <*d*> = 3.51 ± 0.47 nm (13% dispersity).

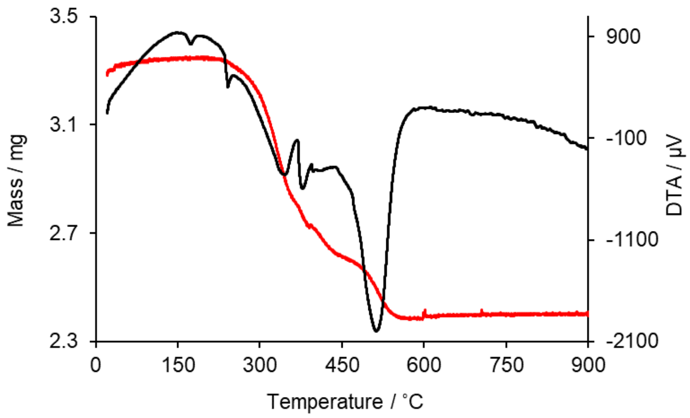

Figure S8. TGA (red curve) and DTA (black curve) plots for AuNP-**2**(1) (3.29 mg). The sample was heated under air at a ramp rate of 10 °C min^{-1} over the range 20–900 °C.

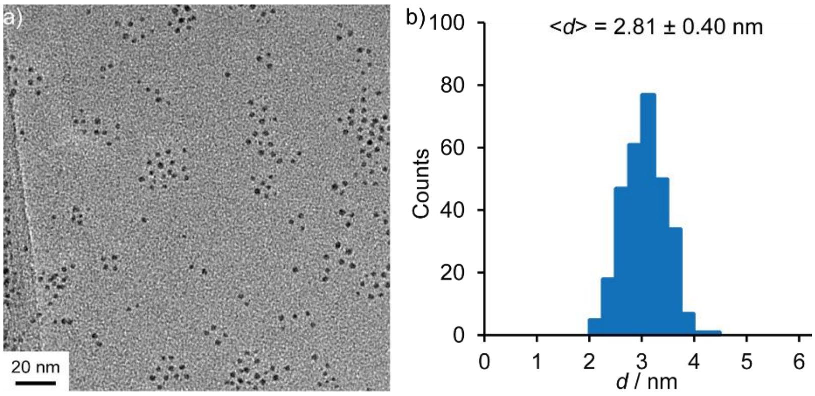

Figure S9. Representative TEM micrograph (scale bar 20 nm) for AuNP-**2**(2). Size distribution (determined from > 300 particle measurements): <*d*> = 2.81 ± 0.40 nm (14% dispersity).

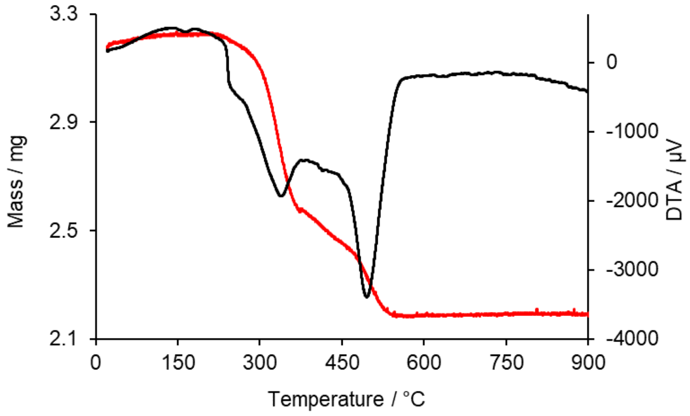

Figure S10. TGA (red curve) and DTA (black curve) plots for AuNP-**2**(2) (4.74 mg). The sample was heated under air at a ramp rate of 10 °C min^{-1} over the range 20–900 °C.

Table S1. Summary constitutional characterization for all nanoparticle batches.

Sample	<d_{core}> / nm [a]	NP S.A. / nm^2 [b]	M % [c]	Org. % [c]	M atoms per NP	Ligands per NP	Monolayer density (ligands per nm^2)
AuNP-**1**	3.20 ± 0.38	32.2	69.1	30.9	1012	138	4.3
PdNP-**1**(1)	2.66 ± 0.39	22.2	34.8	65.2	664	216	9.7
PdNP-**1**(2)	2.34 ± 0.41	17.2	54.6	45.4	450	65	3.8
AuNP-**2**(1)	3.51 ± 0.47	38.7	73.0	27.0	1336	149	3.9
AuNP-**2**(2)	2.81 ± 0.40	24.7	70.1	29.9	682	92	3.7

[a] Mean and standard deviation of size distribution determined from (S)TEM images (Figures S1, S3, S5, S7, S9).
[b] Estimate of nanoparticle surface area calculated assuming a perfect sphere with diameter equal to the sample mean from (S)TEM analysis.
[c] Metal (M) and organic (Org.) content determined by TGA (Figures S2, S4, S6, S8, S10).

4. Linker-driven assembly of individual DCNP building blocks

Solution **A** was prepared volumetrically to give an accurately known concentration of CF_3CO_2H:

A. CF_3CO_2H in 9:1 *v*/*v* DMF/H_2O (4.0 M).

Stock solutions of PdNP-**1**, AuNP-**1** and AuNP-**2** were separately prepared by dissolving a portion (ca. 2 mg) of dried nanoparticles in DMF/D_2O (9:1 *v*/*v*, 2 mL). Concentrations of surface-bound hydrazones were assessed by $^{19}F\{^1H\}$ NMR spectroscopy relative to 4-fluorotoluene as internal standard, which was added at a known concentration (ca. 5 mM). Solutions **B**, **C** and **D** were then prepared volumetrically to give accurately known concentrations of surface-bound **1** and **2**, respectively:

B. PdNP-**1** in 9:1 *v*/*v* DMF/H_2O (0.15 mM surface-bound **1**).

C. AuNP-**1** in 9:1 *v*/*v* DMF/H_2O (0.15 mM surface-bound **1**).

D. AuNP-**2** in 9:1 *v*/*v* DMF/H_2O (0.15 mM surface-bound **2**).

Each solution was than sonicated for 10 minutes and filtered (Whatman Puradisc 13, polypropylene, 100 nm).

The following mixtures were prepared to contain a final overall concentration of 0.15 mM in terms of surface-bound hydrazones.

Nucleophilic PdNP-1 with dialdehyde linker 3: 0.15 mM PdNP-**1** (**B**, 4.0 mL) + 0.45 mM **3** (0.25 mg) + 20 mM CF_3CO_2H (**A**, 15.0 μL).

Nucleophilic AuNP-1 with dialdehyde linker 3: 0.15 mM AuNP-**1** (**C**, 4.0 mL) + 0.45 mM **3** (0.25 mg) + 20 mM CF_3CO_2H (**A**, 15.0 μL).

Electrophilic AuNP-2 with dihydrazide linker 4: 0.15 mM AuNP-**2** (**D**, 4.0 mL) + 0.45 mM **4** (0.35 mg) + 20 mM CF_3CO_2H (**A**, 15.0 μL).

Control 1 (no acid): 0.15 mM PdNP-**1** (**B**, 4.00 mL) + 0.45 mM **3** (0.25 mg).

Control 2 (no linker): 0.15 mM PdNP-**1** (**B**, 4.00 mL) + 20 mM CF_3CO_2H (**A**, 15.0 μL).

Control 3 (no acid): 0.15 mM AuNP-**1** (**C**, 4.00 mL) + 0.45 mM **3** (0.25 mg).

Control 4 (no linker): 0.15 mM AuNP-**1** (**C**, 4.00 mL) + 20 mM CF_3CO_2H (**A**, 15.0 μL).

Control 5 (no acid): 0.15 mM AuNP-**2** (**D**, 4.00 mL) + 0.45 mM **4** (0.35 mg).

Control 6 (no linker): 0.15 mM AuNP-**2** (**D**, 4.00 mL) + 20 mM CF_3CO_2H (**A**, 15.0 μL).

Samples were kept at room temperature throughout the experiment time course.

The concentration of colloidally stable material was monitored by UV-vis absorbance spectroscopy without any further dilution. The first absorption spectrum (t = 0) was recorded prior to addition of acid (where present), and subsequent spectra recorded at 24 h intervals.

Solvodynamic size distribution was monitored by dynamic light scattering (DLS). Samples were prepared by collecting 150 μL of solution previously subjected to UV-vis absorbance spectroscopy, then diluting to a final volume of 2.00 mL, using fresh DMF/H_2O (1.85 mL, 9:1 *v*/*v*).

Samples of colloidally stable nanoparticles were prepared for electron microscopy by depositing one drop of nanoparticle suspension (solution previously subjected to UV-vis absorbance spectroscopy) onto a TEM grid sitting on a lint-free tissue. To image insoluble material, the sample was first sonicated for 5 min before withdrawing a drop of the resulting suspension, which was deposited on the TEM grid. All grids were left to dry at ambient pressure and temperature before imaging.

Nucleophilic PdNP-1 with dialdehyde linker 3

Initial concentrations: [PdNP-**1**(1)]$_0$ = 0.15 mM, [**3**]$_0$ = 0.45 mM, [CF_3CO_2H]$_0$ = 20 mM in 9:1 *v*/*v* DMF/H_2O.

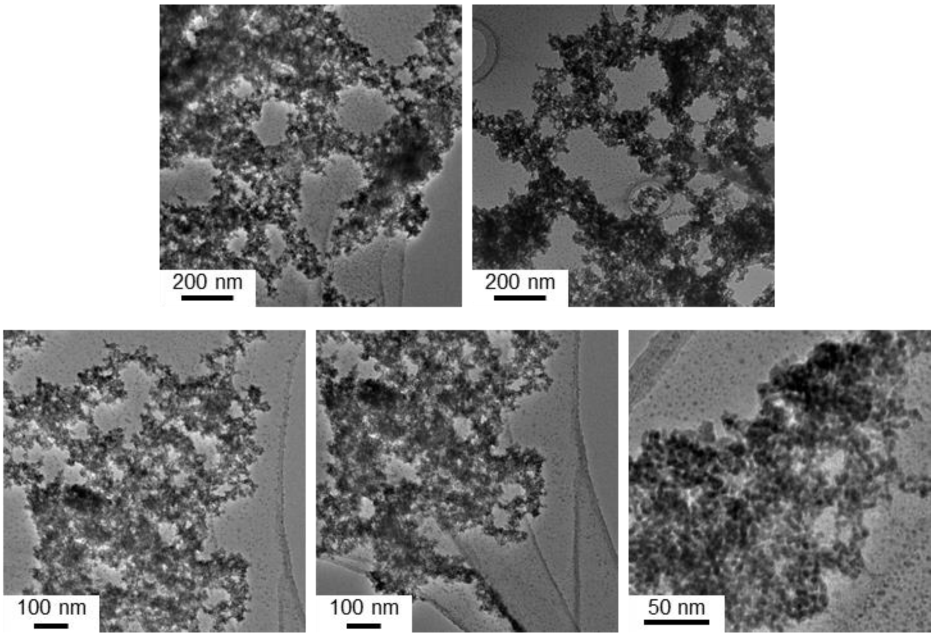

Figure S11. Supplementary TEM images for assemblies produced at day 6 from the combination of PdNP-**1**(1) with bisaldehyde linker **3**.

Nucleophilic AuNP-1 with dialdehyde linker 3

Initial concentrations: [AuNP-**1**]$_0$ = 0.15 mM, [**3**]$_0$ = 0.45 mM, [CF_3CO_2H]$_0$ = 20 mM in 9:1 *v*/*v* DMF/H_2O.

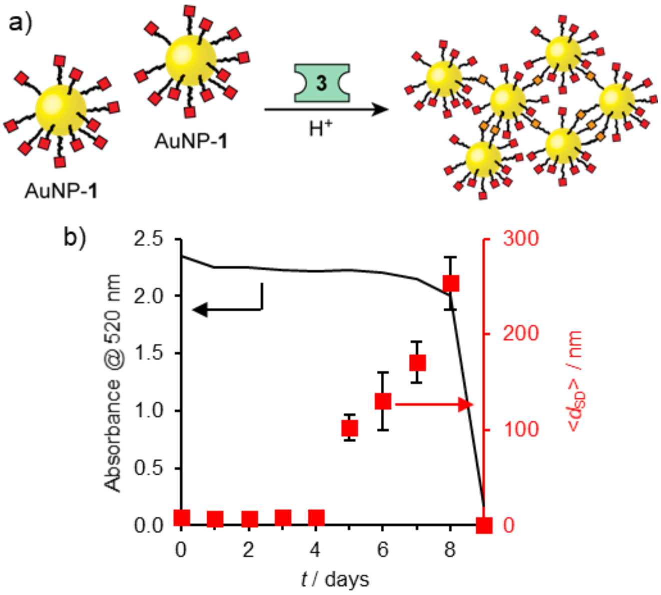

Figure S12. (a) Linker-driven assembly of nucleophilic AuNP-**1** using dialdehyde linker **3**. (b) Solvodynamic diameter (<d_{SD}>, symbols) as measured by DLS and absorbance at 520 nm evolution over time after addition of the acid catalyst required for dynamic covalent hydrazone exchange. Conditions: [AuNP-**1**]$_0$ = 0.15 mM, [**3**]$_0$ = 0.45 mM, [CF_3CO_2H]$_0$ = 20 mM, 9:1 *v*/*v* DMF/H_2O.

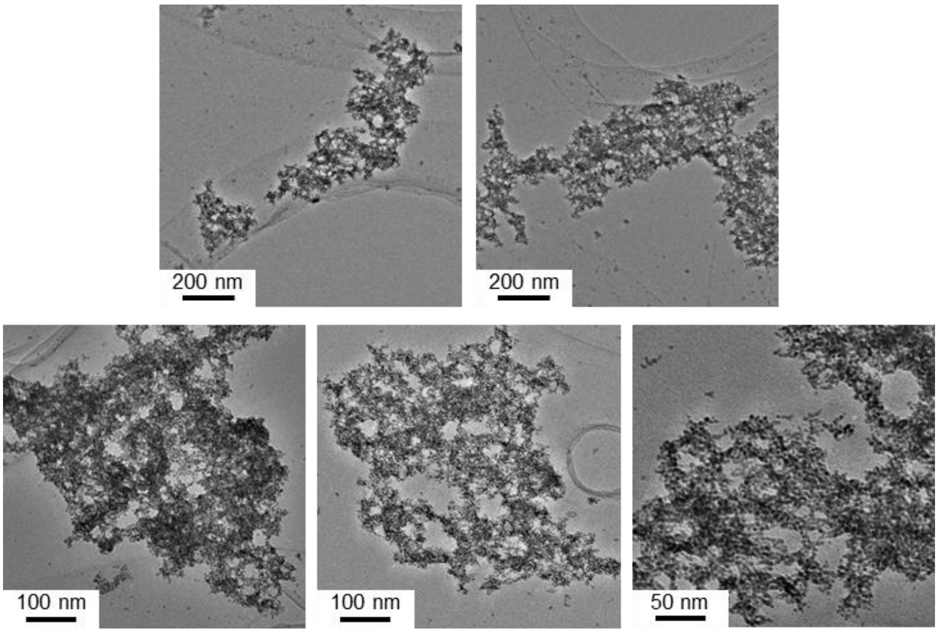

Figure S13. TEM images for assemblies produced at day 9 from the combination of AuNP-**1** with bisaldehyde linker **3**.

Electrophilic AuNP-2 with dihydrazide linker 4

Initial concentrations: [AuNP-**2**]$_0$ = 0.15 mM, [**4**]$_0$ = 0.45 mM, [CF_3CO_2H]$_0$ = 20 mM in 9:1 *v*/*v* DMF/H_2O

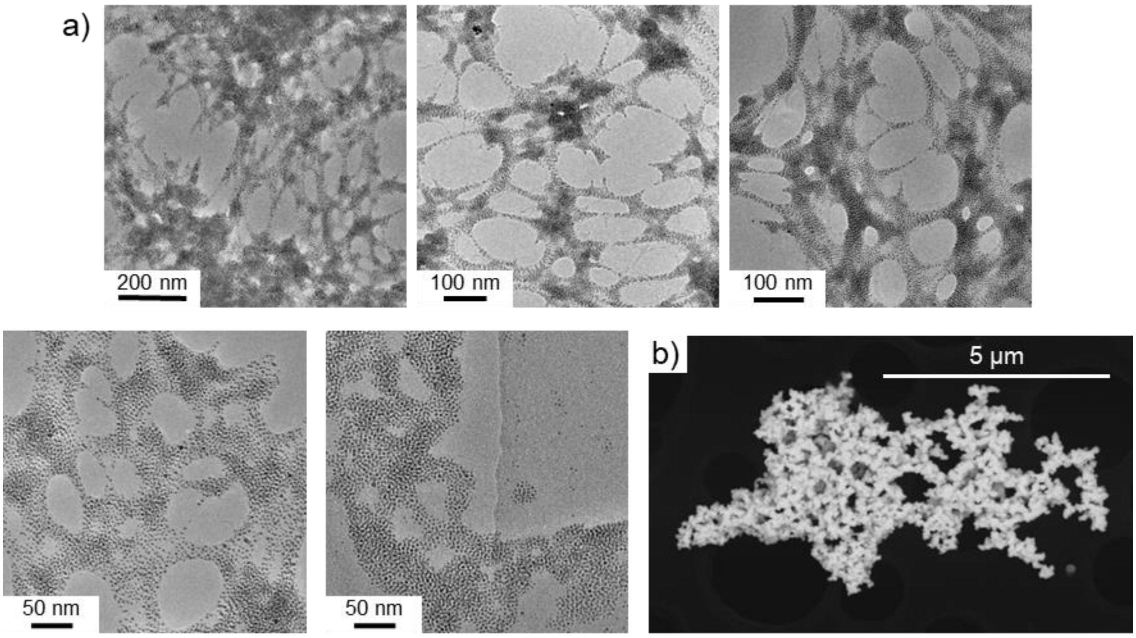

Figure S14. Supplementary (a) TEM and (b) SEM images for assemblies produced after 7 h from the combination of AuNP-**2** with dihydrazide linker **4**.

Nucleophilic PdNP-1 – control experiments 1 and 2

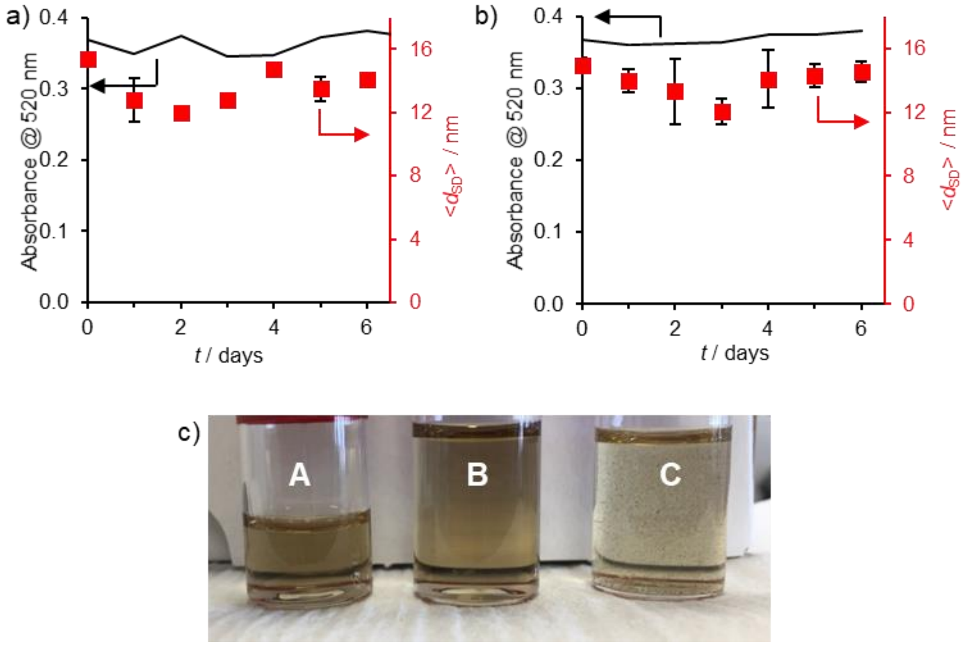

Figure S15. Control experiments for linker-driven assembly of nucleophilic PdNP-**1**. (a, b) Solvodynamic diameter ($<d_{SD}>$, symbols) as measured by DLS and absorbance at 520 nm evolution over time after addition of the acid catalyst required for dynamic covalent hydrazone exchange. Conditions. (a): [PdNP-**1**]$_0$ = 0.15 mM, [**3**]$_0$ = 0 mM, [CF_3CO_2H]$_0$ = 20 mM, 9:1 *v*/*v* DMF/H_2O. (b): [PdNP-**1**]$_0$ = 0.15 mM, [**3**]$_0$ = 0.45 mM, [CF_3CO_2H]$_0$ = 0 mM, 9:1 *v*/*v* DMF/H_2O. (c) Photographs of PdNP-**1** in 9:1 *v*/*v* DMF/H_2O after 6 days incubation at room temperature, in the presence of: (A) CF_3COOH only; (B) ditopic terephthalaldehyde linker **3** only; (3) both CF_3COOH and terephthalaldehyde linker **3**, showing precipitation of colloidally unstable aggregates only in the latter experiment.

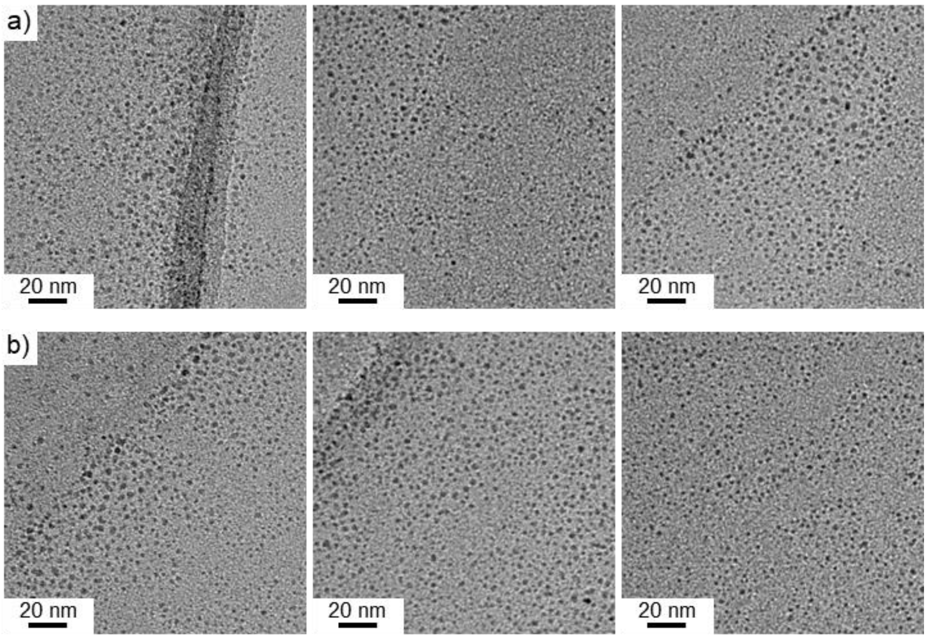

Figure S16. Representative TEM images of PdNP-**1** after 6 days incubation at room temperature, in the presence of: (a) CF_3COOH only; (b) ditopic terephthalaldehyde linker **3** only, showing well-dispersed particles in both cases.

Nucleophilic AuNP-1 – control experiments 3 and 4

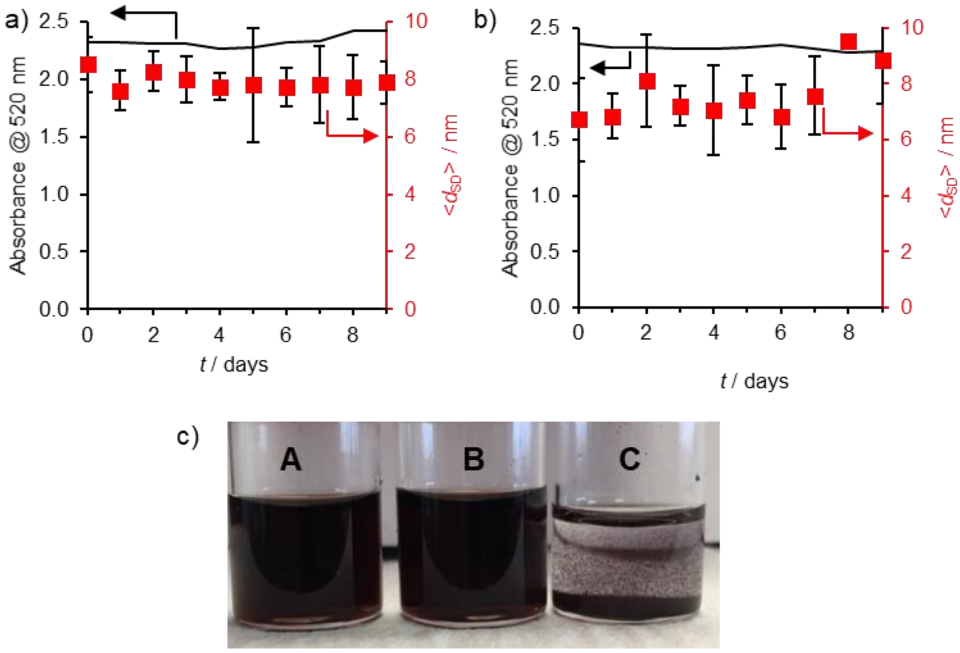

Figure S17. Control experiments for linker-driven assembly of nucleophilic AuNP-**1**. (a, b) Solvodynamic diameter (<d_{SD}>, symbols) as measured by DLS and absorbance at 520 nm evolution over time after addition of the acid catalyst required for dynamic covalent hydrazone exchange. Conditions. (a): [AuNP-**1**]$_0$ = 0.15 mM, [**3**]$_0$ = 0 mM, [CF_3CO_2H]$_0$ = 20 mM, 9:1 *v*/*v* DMF/H_2O. (b): [AuNP-**1**]$_0$ = 0.15 mM, [**3**]$_0$ = 0.45 mM, [CF_3CO_2H]$_0$ = 0 mM, 9:1 *v*/*v* DMF/H_2O. (c) Photographs of AuNP-**1** in 9:1 *v*/*v* DMF/H_2O after 9 days incubation at room temperature, in the presence of: (A) CF_3COOH only; (B) ditopic terephthalaldehyde linker **3** only; (3) both CF_3COOH and terephthalaldehyde linker **3**, showing precipitation of colloidally unstable aggregates only in the latter experiment.

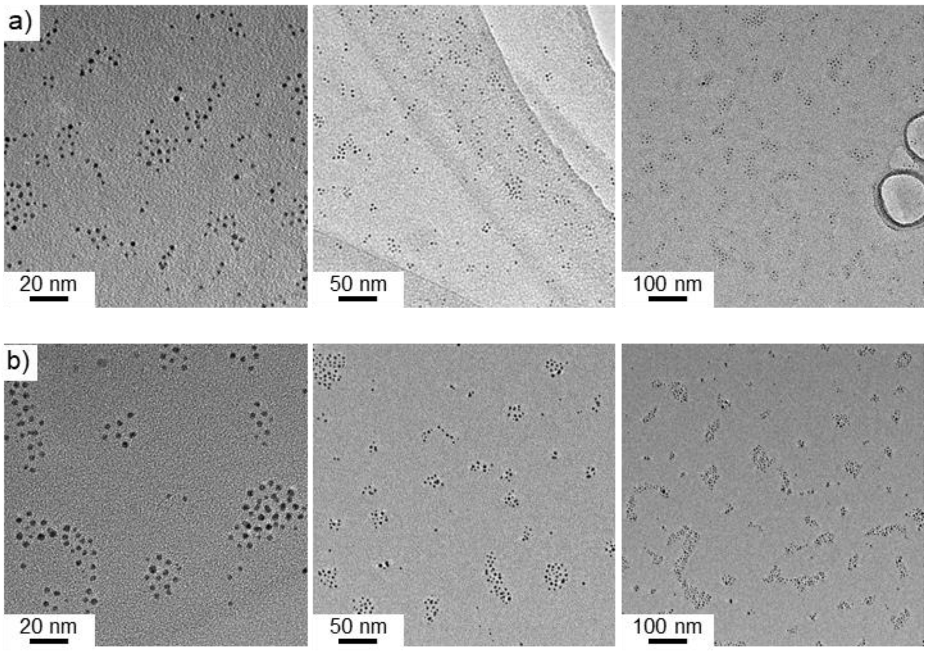

Figure S18. Representative TEM images of AuNP-**1** after 9 days incubation at room temperature, in the presence of: (a) CF_3COOH only; (b) ditopic terephthalaldehyde linker **3** only, showing well-dispersed particles in both cases.

Electrophilic AuNP-2 – control experiments 5 and 6

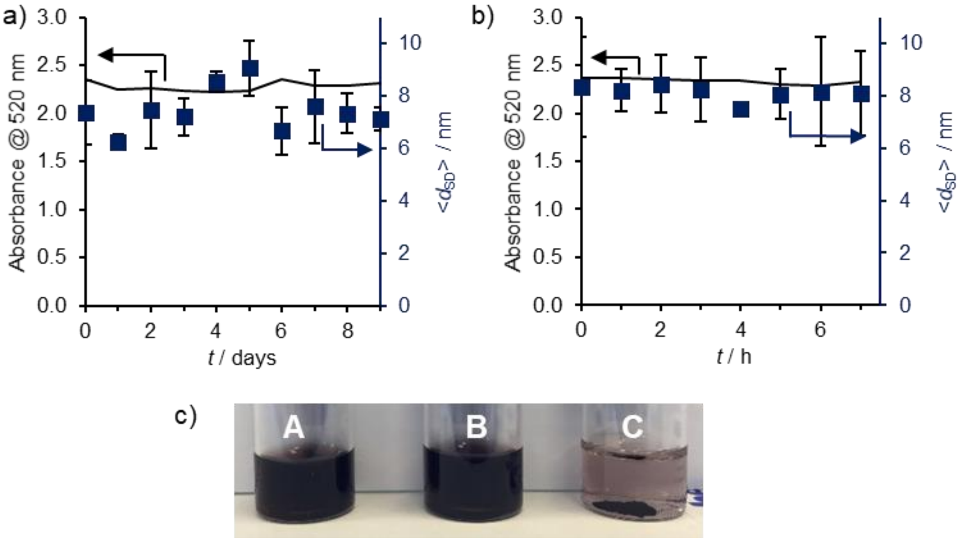

Figure S19. Control experiments for linker-driven assembly of nucleophilic AuNP-**2**. (a, b) Solvodynamic diameter (<d_{SD}>, symbols) as measured by DLS and absorbance at 520 nm evolution over time after addition of the acid catalyst required for dynamic covalent hydrazone exchange. Conditions. (a): [AuNP-**2**]$_0$ = 0.15 mM, [**4**]$_0$ = 0 mM, [CF_3CO_2H]$_0$ = 20 mM, 9:1 *v*/*v* DMF/H_2O. (b): [AuNP-**2**]$_0$ = 0.15 mM, [**4**]$_0$ = 0.45 mM, [CF_3CO_2H]$_0$ = 0 mM, 9:1 *v*/*v* DMF/H_2O. (c) Photographs of AuNP-**2** in 9:1 *v*/*v* DMF/H_2O after 7 h incubation at room temperature, in the presence of: (A) CF_3COOH only; (B) ditopic dihydrazide linker **4** only; (3) both CF_3COOH and dihydrazide linker **4**, showing precipitation of colloidally unstable aggregates only in the latter experiment.

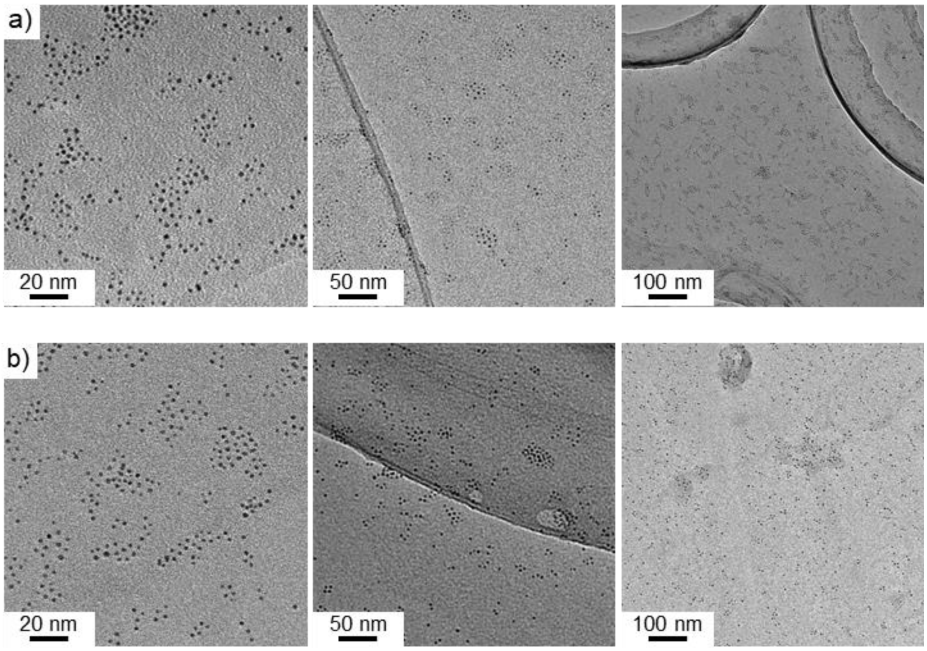

Figure S20. Representative TEM images of AuNP-**2** after incubation at room temperature, in the presence of: (a) CF_3COOH only for 17 days; (b) ditopic bishydrazide linker **4** only for 3 days, showing well-dispersed particles in both cases.

5. Complementary and selective heteromaterial assembly from binary DCNP mixtures

Table S2. Initial states and aggregate outcomes for heteromaterial assembly experiments under co-assembly (experiments C1, C2), nucleophilic linker-driven (experiments LN1–LN5) and electrophilic linker-driven (LE1–LE3) conditions from mixtures of AuNP-**1** and PdNP-**2**.

**Exp.**	**NP Input**	**Molecular Input**		**Water**	**Aggregate Outcome** [b]	
	PdNP : AuNP	[**1**] : [**2**]	Linker [a]		PdNP : AuNP [c]	*E.F.* [d]
Co-assembly (PdNP-**1** + AuNP-**2**)						
C1	50 : 50	58 : 42	none	10%	46 : 54	0.07
C2	41 : 59	50 : 50	none	10%	35 : 65	0.11
Nucleophilic linker-driven (PdNP-**1** + AuNP-**2** + linker **4**)						
LN1	50 : 50	58 : 42	6 eq	10%	25 : 75	0.51
LN2	50 : 50	58 : 42	6 eq	0%	13 : 87	0.73
LN3	41 : 59	50 : 50	6 eq	10%	7 : 93	0.57
LN4	41 : 59	50 : 50	6 eq	0%	4 : 96	0.62
LN5[e]	59 : 41	50 : 50	3 eq	10%	8 : 92	1.2
Electrophilic linker driven (PdNP-**1** + AuNP-**2** + linker **3**)						
LE1	41 : 59	50 : 50	6 eq	10%	58 : 42	–0.28
LE2	50 : 50	58 : 42	4.3 eq	10%	87 : 13	–0.74
LE3[e]	59 : 41	50 : 50	3 eq	10%	16 : 84	1.1

[a] Molar equivalents with respect to complementary NP-bound hydrazone (i.e., **3** relative to PdNP-**1**; **4** relative to AuNP-**2**).
[b] Aggregates isolated at 5 days (experiments C1, LN1–LN4); 6 days (LN5); 8 days (C2); 10 days (experiments LE1–LE3).
[c] Expressed as NP molar ratio.
[d] Enrichment factor in nanoparticle ratio adjusted for input state: $[F_{Au(aggregate)} - F_{Au(input)}] / F_{Au(input)}$.
[e] Experiment performed with PdNP-**1**(2) and AuNP-**2**(2).

Solutions **A** and **B** were prepared volumetrically to give accurately known concentrations of CF_3CO_2H:

A. CF_3CO_2H in 9:1 *v*/*v* DMF/H_2O (4 M).

B. CF_3CO_2H in anhydrous DMF (4 M).

Stock solutions of PdNP-**1**, AuNP-**1** and AuNP-**2** were separately prepared by dissolving a portion (ca. 2 mg) of dried nanoparticles in DMF/D_2O (9:1 *v*/*v*, 2 mL) or anhydrous DMF (2 mL). Concentrations of surface-bound hydrazones were assessed by $^{19}F\{^1H\}$ NMR spectroscopy relative to 4-fluorotoluene as internal standard, which was added at a known concentration (ca. 5 mM). Solutions **C**, **D**, **E, F, G, H, I** and **L** were then prepared volumetrically to give accurately known concentrations of NP-bound **1** and **2** respectively:

C. PdNP-**1** in 9:1 *v*/*v* DMF/H_2O (0.15 mM NP-bound **1**).

D. AuNP-**1** in 9:1 *v*/*v* DMF/H_2O (0.15 mM NP-bound **1**).

E. AuNP-**2** in 9:1 *v*/*v* DMF/H_2O (0.15 mM NP-bound **2**).

F. PdNP-**1** in 9:1 *v*/*v* DMF/H_2O (0.21 mM NP-bound **1**).

G. PdNP-**1** in anhydrous DMF (0.15 mM NP-bound **1**).

H. AuNP-**1** in anhydrous DMF (0.15 mM NP-bound **1**).

I. AuNP-**2** in anhydrous DMF (0.15 mM NP-bound **2**).

L. PdNP-**1** in anhydrous DMF (0.21 mM NP-bound **1**).

Each solution was than sonicated for 10 minutes and filtered (Whatman Puradisc 13, polypropylene, 100 nm).

The following mixtures were prepared to contain **equimolar concentration of the two NP-bound hydrazones** (**1** and **2**) at a final overall concentration of 0.15 mM.

Experiment C2 (heteromaterial co-assembly): 0.075 mM PdNP-**1** (**C**, 2.0 mL) + 0.075 mM AuNP-**2** (**E**, 2.0 mL) + 20 mM CF_3CO_2H (**A**, 15.0 µL). Results: Figures 3c,f–g, S22 and Table S4.

Experiment HC2 (co-assembly from homomaterial mixture): 0.075 mM AuNP-**1** (**D**, 2.0 mL) + 0.075 mM AuNP-**2** (**E**, 2.0 mL) + 20 mM CF_3CO_2H (**A**, 15.0 µL). Results: Figure S23.

Experiment LN3 (selective assembly of AuNP-2): 0.075 mM PdNP-**1** (**C**, 2.0 mL) + 0.075 mM AuNP-**2** (**E**, 2.0 mL) + 0.45 mM **4** (0.35 mg) + 20 mM CF_3CO_2H (**A**, 15.0 µL). Results: Figures S26b–c, S27 and Table S7.

Experiment LN4 (selective assembly of AuNP-2, anhydrous): 0.075 mM PdNP-**1** (**G**, 2.0 mL) + 0.075 mM AuNP-**2** (**I**, 2.0 mL) + 0.45 mM **4** (0.35 mg) + 20 mM CF_3CO_2H (**B**, 15.0 µL). Results: Figures S26d–e, S28 and Table S8.

Experiment LN5 (selective assembly of AuNP-2, low linker concentration): 0.075 mM PdNP-**1** (**C**, 2.0 mL) + 0.075 mM AuNP-**2** (**E**, 2.0 mL) + 0.225 mM **4** (0.18 mg) + 20 mM CF_3CO_2H (**A**, 15.0 µL). Experiment performed with NP batches PdNP-**1**(2) and AuNP-**2**(2). Results: Figures S34, S35.

Experiment HLN3 (selective assembly of AuNP-2 from homomaterial mixture): 0.075 mM AuNP-**1** (**D**, 2.0 mL) + 0.075 mM AuNP-**2** (**E**, 2.0 mL) + 0.45 mM **4** (0.35 mg) + 20 mM CF_3CO_2H (**A**, 15.0 µL). Results: Figure S31b.

Experiment HLN4 (selective assembly AuNP-2 from homomaterial mixture, anhydrous): 0.075 mM AuNP-**1** (**H**, 2.0 mL) + 0.075 mM AuNP-**2** (**I**, 2.0 mL) + 0.45 mM **4** (0.35 mg) + 20 mM CF_3CO_2H (**B**, 15.0 µL). Results: Figure S31c.

Experiment LE1 (selective assembly of PdNP-1): 0.075 mM PdNP-**1** (**C**, 2.0 mL) + 0.075 mM AuNP-**2** (**E**, 2.0 mL) + 0.45 mM **4** (0.25 mg) + 20 mM CF_3CO_2H (**A**, 15.0 µL). Results: Figures 5b–c, S32 and Table S10.

Experiment LE3 ('selective' assembly of PdNP-1, low linker concentration): 0.075 mM PdNP-**1** (**C**, 2.0 mL) + 0.075 mM AuNP-**2** (**E**, 2.0 mL) + 0.225 mM **4** (0.13 mg) + 20 mM CF_3CO_2H (**A**, 15.0 µL). Experiment performed with NP batches PdNP-**1**(2) and AuNP-**2**(2). Results: Figures S34, S35, Table S12.

Experiment HLE1 (selective assembly of AuNP-1 from homomaterial mixture): 0.075 mM AuNP-**1** (**D**, 2.0 mL) + 0.075 mM AuNP-**2** (**E**, 2.0 mL) + 0.45 mM **3** (0.25 mg) + 20 mM CF_3CO_2H (**A**, 15.0 µL). Results: Figures S36.

Control 7 (C2, no acid): 0.075 mM PdNP-**1** (**C**, 2.0 mL) + 0.075 mM AuNP-**2** (**E**, 2.0 mL). Results: Figures S37b–c.

The following mixtures were prepared to contain an **equimolar concentration of nucleophilic and electrophilic nanoparticles**. Concentrations of surface surface-bound **1** and **2** are consequently not equimolar in these experiments.

Experiment C1 (heteromaterial co-assembly): 0.105 mM PdNP-**1** (**F**, 2.0 mL) + 0.075 mM AuNP-**2** (**E**, 2.0 mL) + 20 mM CF_3CO_2H (**A**, 15.0 µL). Results: Figures 3b,d–e, S21, Table S3.

Experiment LN1 (selective assembly of AuNP-2): 0.105 mM PdNP-**1** (**F**, 2.0 mL) + 0.075 mM AuNP-**2** (**E**, 2.0 mL) + 0.45 mM **4** (0.35 mg) + 20 mM CF_3CO_2H (**A**, 15.0 µL). Results: Figures 4b–c, S24, Table S5.

Experiment LN2 (selective assembly of AuNP-2, anhydrous): 0.105 mM PdNP-**1** (**L**, 2.0 mL) + 0.075 mM AuNP-**2** (**I**, 2.0 mL) + 0.45 mM **4** (0.35 mg) + 20 mM CF_3CO_2H (**B**, 15.0 µL). Results: Figures 4d–e, S25, Table S6.

Experiment LE2 (selective assembly of PdNP-1): 0.105 mM PdNP-**1** (**F**, 2.0 mL) + 0.075 mM AuNP-**2** (**E**, 2.0 mL) + 0.45 mM **3** (0.25 mg) + 20 mM CF_3CO_2H (**A**, 15.0 µL). Figures 5d–e, S33, Table S11.

Control 8 (C1, no acid): 0.105 mM PdNP-**1** (**F**, 2.0 mL) + 0.075 mM AuNP-**2** (**E**, 2.0 mL). Results: Figures S37a, c.

Samples were kept at room temperature throughout the experiment time course.

Changes in the concentration of colloidally stable material were monitored by UV-vis absorbance spectroscopy without any further dilution. The first absorption spectrum (t = 0) was recorded prior addition of acid catalyst (where present), and subsequent spectra recorded at 24 h intervals.

Solvodynamic size distribution was monitored by dynamic light scattering. Samples were prepared by collecting 150 µL of solution previously subjected to UV-vis absorbance spectroscopy, then diluting to a final volume of 2.00 mL, using fresh 9:1 *v*/*v* DMF/H_2O (1.85 mL) or fresh anhydrous DMF (1.85 mL, for experiments in absence of water).

For analysis by electron microscopy, insoluble extended aggregates formed as a result of complementary (complete precipitation of NP material) and selective (significant quantity of suspended solid) assembly were first isolated and purified from any unbound NPs. The precipitates were collected by centrifugation. The recovered black solid was then washed using the following procedure: solid was dispersed in DMF (4 mL), sonicated (10 min, 20 °C), then recollected by centrifugation (1935 ×g rcf, 15 min, rt). The same operation was repeated a further twice. Finally, each sample was suspended in fresh DMF (2 mL), sonicated for 5 min before withdrawing a drop of the resulting suspension and spotting onto a TEM grid. All grids were left to dry at ambient pressure and temperature before imaging.

Aggregate material composition was assessed by EDX spectroscopy at a minimum of three distinct areas of the grid. Spectra were acquired over 120 s. Atomic percentages were determined by fitting the emission lines for Pd(L) and Au(L). The relative atomic percentages were then converted to a ratio of nanoparticles using the average number of metal atoms in each particle as determined from (S)TEM size analysis (Table S1).

Experiments C1, C2: Co-assembly

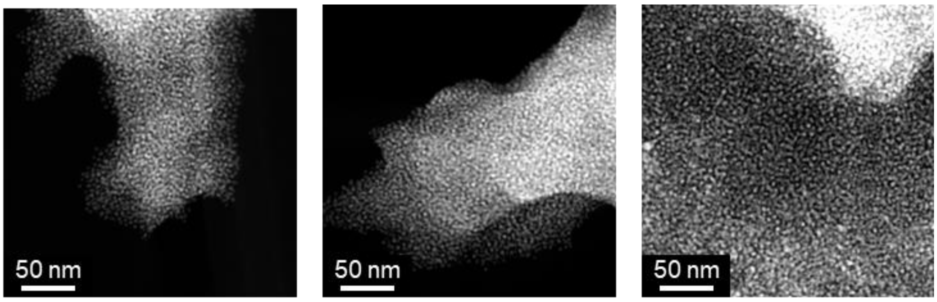

Figure S21. Experiment C1 representative HAADF images for assemblies produced at day 6 from the complementary assembly of PdNP-**1** with AuNP-**2** (from initial state [**1**]:[**2**] = 58:42; PdNP:AuNP = 50:50). EDX spectra were acquired from a region in the centre of each of these areas to give the results reported in Table S3.

Table S3. Experiment C1 heteromaterial aggregate composition and enrichment factor (*E.F.*) determined by EDX mapping on three distinct sample regions of aggregates produced at day 6 from the complementary assembly of PdNP-**1** with AuNP-**2** (from initial state [**1**]:[**2**] = 58:42; PdNP:AuNP = 50:50).

C1	Pd atom%	Au atom%	F_{PdNP}	F_{AuNP}
**Input**	33	67	0.50	0.50
Area 1	28.38	71.61	0.444	0.556
Area 2	32.77	67.22	0.495	0.505
Area 3	28.93	71.06	0.450	0.550
**Average**	**30.0**	**70.0**	**0.46**	**0.54**
s.d.	2.4	2.4	0.03	0.03
			E.F.	**0.07**

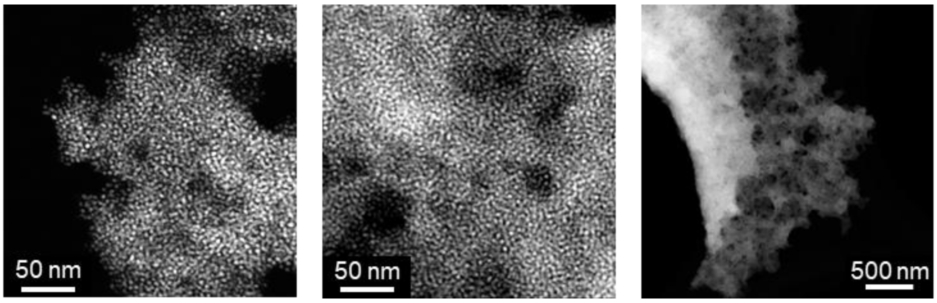

Figure S22. Experiment C2 representative HAADF images for assemblies produced at day 8 from the complementary assembly of PdNP-**1** with AuNP-**2** from initial state [**1**]:[**2**] = 50:50; PdNP:AuNP = 41:59). EDX spectra were acquired from a region in the centre of each of these areas to give the results reported in Table S4.

Table S4. Experiment C2 heteromaterial aggregate composition and enrichment factor (*E.F.*) determined by EDX mapping on three distinct sample regions of aggregates produced at day 8 from the complementary assembly of PdNP-**1** with AuNP-**2** (from initial state [**1**]:[**2**] = 50:50; PdNP:AuNP = 41:59).

C2	Pd atom%	Au atom%	F_{PdNP}	F_{AuNP}
**Input**	26	74	0.41	0.59
Area 1	21.19	78.80	0.351	0.649
Area 2	20.15	79.84	0.337	0.663
Area 3	21.43	78.56	0.354	0.646
**Average**	**20.9**	**79.1**	**0.35**	**0.65**
s.d.	0.7	0.7	0.01	0.01
			E.F.	**0.11**

Complementary co-assembly from a homomaterial mixture

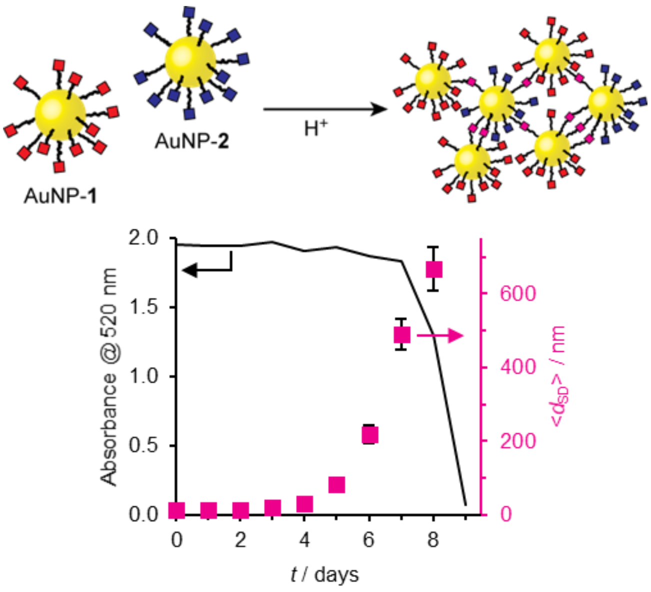

Figure S23. (a) Experiment HC2. Co-assembly from a homomaterial colloidal mixture, equimolar in terms of NP-bound ligands. (b) Variation in solvodynamic diameter (<d_{SD}>, magenta symbols) as measured by DLS and absorbance at 520 nm (line) over time after addition of the acid catalyst required for dynamic covalent hydrazone exchange. Assembly conditions (concentrations in terms of molecular species): [AuNP-**1**]$_0$ = 0.075 mM, [AuNP-**2**]$_0$ = 0.075 mM, [CF_3CO_2H]$_0$ = 20 mM, 9:1 *v*/*v* DMF/H_2O.

Experiments LN1–LN5: Nucleophilic linker selected assembly

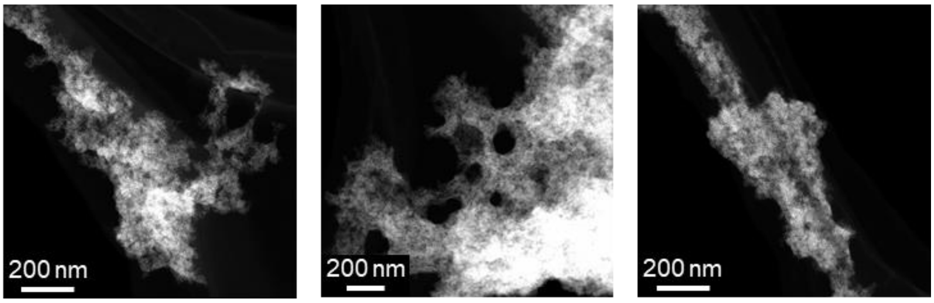

Figure S24. Experiment LN1 representative HAADF images for assemblies produced at day 5 from the nucleophilic linker-driven assembly of from a mixture of PdNP-**1**, AuNP-**2** and **4** (initial state [**1**]:[**2**]:[**4**] = 1.4:1:6; PdNP:AuNP = 50:50). EDX spectra were acquired from a region in the centre of each of these areas to give the results reported in Table S5.

Table S5. Experiment LN1 heteromaterial aggregate composition and enrichment factor (*E.F.*) determined by EDX mapping on three distinct sample regions of aggregates produced at day 5 from the nucleophilic linker-driven assembly of from a mixture of PdNP-**1**, AuNP-**2** and **4** (initial state [**1**]:[**2**]:[**4**] = 1.4:1:6; PdNP:AuNP = 50:50).

LN1	Pd atom%	Au atom%	F_{PdNP}	F_{AuNP}
**Input**	33	67	0.50	0.50
Area 1	12.77	87.22	0.228	0.772
Area 2	14.43	85.56	0.253	0.747
Area 3	14.67	85.32	0.257	0.743
**Average**	**14.0**	**86.0**	**0.25**	**0.75**
s.d.	1.0	1.0	0.02	0.02
			E.F.	**0.51**

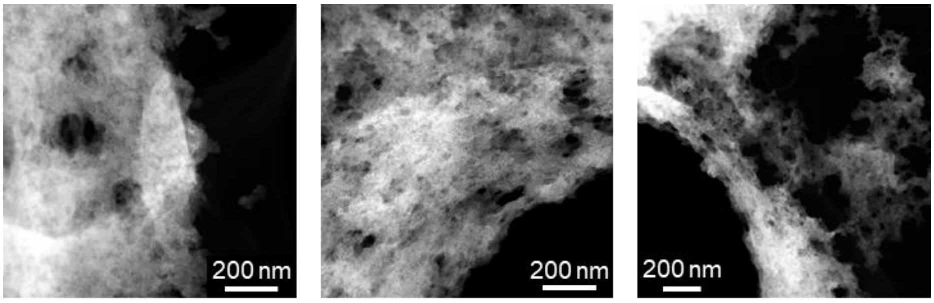

Figure S25. Experiment LN2 representative HAADF images for assemblies produced at day 5 from the nucleophilic linker-driven assembly of from a mixture of PdNP-**1**, AuNP-**2** and **4** (initial state [**1**]:[**2**]:[**4**] = 1.4:1:6; PdNP:AuNP = 50:50) in the absence of water. EDX spectra were acquired from a region in the centre of each of these areas to give the results reported in Table S6.

Table S6. Experiment LN2 heteromaterial aggregate composition and enrichment factor (*E.F.*) determined by EDX mapping on three distinct sample regions of aggregates produced at day 5 from the nucleophilic linker-driven assembly of from a mixture of PdNP-**1**, AuNP-**2** and **4** (initial state [**1**]:[**2**]:[**4**] = 1.4:1:6; PdNP:AuNP = 50:50) in the absence of water.

**LN2**	**Pd atom%**	**Au atom%**	F_{PdNP}	F_{AuNP}
**Input**	33	67	0.50	0.50
Area 1	6.64	93.35	0.125	0.875
Area 2	5.95	94.04	0.113	0.887
Area 3	9.00	90.99	0.166	0.834
**Average**	**7.2**	**92.8**	**0.13**	**0.87**
s.d.	1.6	1.6	0.03	0.03
		E.F.	**0.73**	

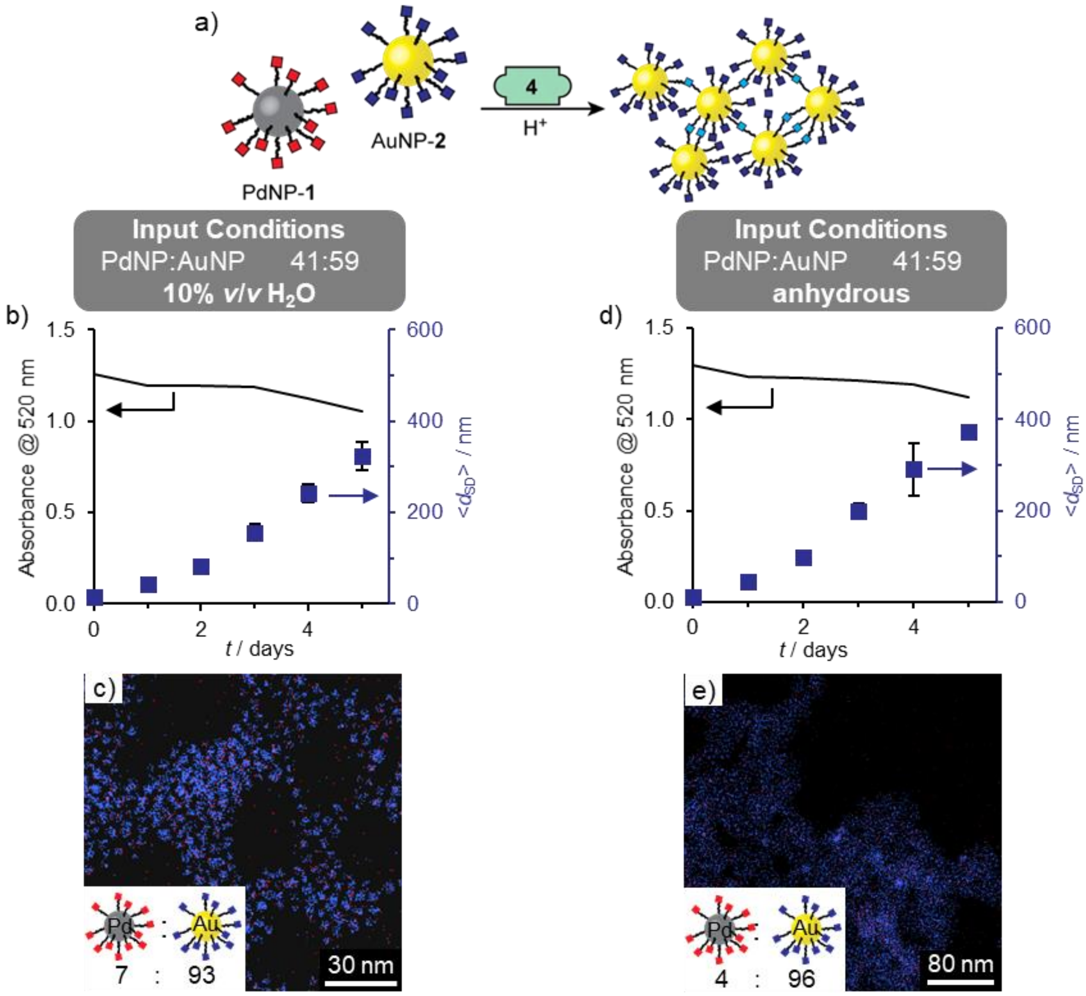

Figure S26. (a) Experiments LN3 and LN4. Selective assembly of AuNPs from a binary colloidal mixture of PdNP-**1** and AuNP-**2** equimolar in terms of NP-bound ligands, under aqueous (b–c) and anhydrous (d–e) conditions. (b,d) Variation in solvodynamic diameter (<d_{SD}>, blue symbols) as measured by DLS and absorbance at 520 nm (line) over time after addition of the acid catalyst required for dynamic covalent hydrazone exchange. (c,e) Representative EDX maps of gold-enriched aggregates (blue = Au; red = Pd). Inset: Heteromaterial aggregate composition expressed in terms of ratio of nanoparticles determined by EDX mapping on a minimum of three distinct sample regions (full results in Tables S7 and S8, with accompanying HAADF images in Figures S27 and S28). Assembly conditions (concentrations in terms of molecular species): $[PdNP\text{-}\mathbf{1}]_0$ = 0.075 mM, $[AuNP\text{-}\mathbf{2}]_0$ = 0.075 mM (i.e., PdNP : AuNP = 41:59), $[\mathbf{4}]_0$ = 0.45 mM, $[CF_3CO_2H]_0$ = 20 mM, room temperature, 9:1 *v*/*v* DMF/H_2O (b–c) or anhydrous DMF (d–e).

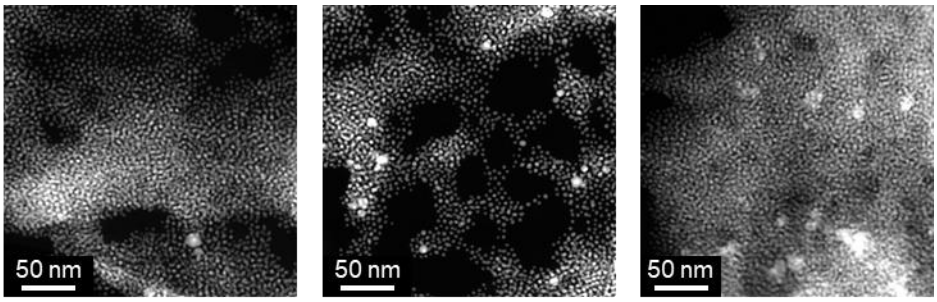

Figure S27. Experiment LN3 representative HAADF images for assemblies produced at day 5 from the nucleophilic linker-driven assembly of from a mixture of PdNP-**1**, AuNP-**2** and **4** (initial state [**1**]:[**2**]:[**4**] = 1:1:6; PdNP:AuNP = 41:59). EDX spectra were acquired from a region in the centre of each of these areas to give the results reported in Table S7.

Table S7. Experiment LN3 heteromaterial aggregate composition and enrichment factor (*E.F.*) determined by EDX mapping on three distinct sample regions of aggregates produced at day 5 from the nucleophilic linker-driven assembly of from a mixture of PdNP-**1**, AuNP-**2** and **4** (initial state [**1**]:[**2**]:[**4**] = 1:1:6; PdNP:AuNP = 41:59).

**LN3**	**Pd atom%**	**Au atom%**	F_{PdNP}	F_{AuNP}
**Input**	26	74	0.41	0.59
Area 1	4.28	95.71	0.083	0.917
Area 2	2.75	97.25	0.054	0.946
Area 3	3.93	96.06	0.076	0.924
**Average**	**3.7**	**96.3**	**0.07**	**0.93**
s.d.	0.8	0.8	0.02	0.02
			E.F.	**0.57**

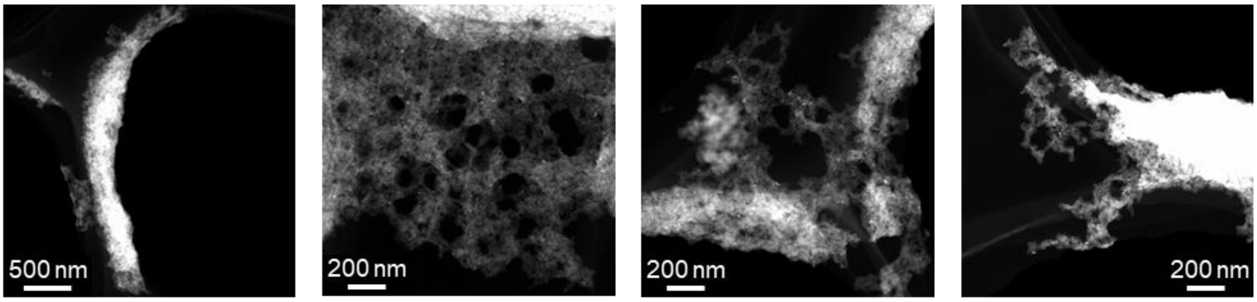

Figure S28. Experiment LN4 representative HAADF images for assemblies produced at day 5 from the nucleophilic linker-driven assembly of from a mixture of PdNP-**1**, AuNP-**2** and **4** (initial state [**1**]:[**2**]:[**4**] = 1:1:6; PdNP:AuNP = 41:59) in the absence of water. EDX spectra were acquired from a region in the centre of each of these areas to give the results reported in Table S8.

Table S8. Experiment LN4 heteromaterial aggregate composition and enrichment factor (*E.F.*) determined by EDX mapping on four distinct sample regions of aggregates produced at day 5 from the nucleophilic linker-driven assembly of from a mixture of PdNP-**1**, AuNP-**2** and **4** (initial state [**1**]:[**2**]:[**4**] = 1:1:6; PdNP:AuNP = 41:59) in the absence of water.

**LN4**	**Pd atom%**	**Au atom%**	F_{PdNP}	F_{AuNP}
**Input**	26	74	0.41	0.59
Area 1	1.19	98.80	0.024	0.976
Area 2	2.01	97.98	0.040	0.960
Area 3	2.40	97.59	0.047	0.953
Area 4	3.25	96.74	0.063	0.937
**Average**	**2.2**	**97.8**	**0.04**	**0.96**
s.d.	0.9	0.9	0.02	0.02
			E.F.	**0.62**

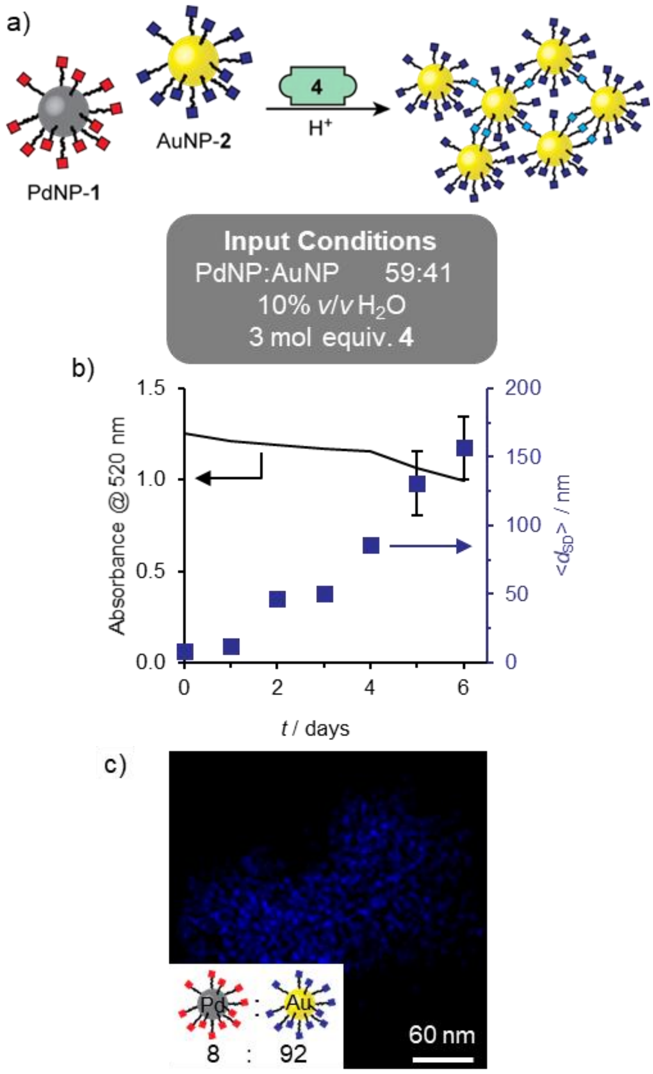

Figure S29. (a) Experiment LN5. Selective assembly of electrophilic AuNPs from a binary colloidal mixture of AuNP-**1** and AuNP-**2**, equimolar in terms of NP-bound ligands using reduced concentration of linker **4**. (b) Variation in solvodynamic diameter (<d_{SD}>, blue symbols) as measured by DLS and absorbance at 520 nm (line) over time after addition of the acid catalyst required for dynamic covalent hydrazone exchange. (c) Representative EDX map of gold-enriched aggregates (blue = Au; red = Pd). Inset: Heteromaterial aggregate composition expressed in terms of ratio of nanoparticles determined by EDX mapping on three distinct sample regions (full results in Table S9, with accompanying HAADF images in Figure S30). Assembly conditions (concentrations in terms of molecular species): [AuNP-**1**(2)]$_0$ = 0.075 mM, [AuNP-**2**(2)]$_0$ = 0.075 mM, (i.e. PdNP : AuNP = 59:41), [**4**]$_0$ = 0.225 mM, [CF_3CO_2H]$_0$ = 20 mM, 9:1 *v*/*v* DMF/H_2O.

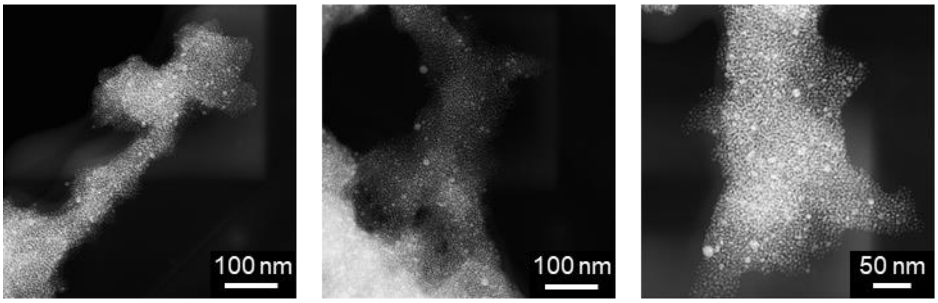

Figure S30. Experiment LN5 representative HAADF images for assemblies produced at day 6 from the nucleophilic linker-driven assembly of from a mixture PdNP-**1**(2), AuNP-**2**(2) and **4** (initial state [**1**]:[**2**]:[**4**] = 1:1:3; PdNP:AuNP = 59:41). EDX spectra were acquired from a region in the centre of each of these areas to give the results reported in Table S9.

Table S9. Experiment LN5 heteromaterial aggregate composition and enrichment factor (*E.F.*) determined by EDX mapping on three distinct sample regions of aggregates produced at day 6 from the nucleophilic linker-driven assembly of from a mixture of PdNP-**1**(2), AuNP-**2**(2) and **4** (initial state [**1**]:[**2**]:[**4**] = 1:1:3; PdNP:AuNP = 59:41).

**LN5**	**Pd atom%**	**Au atom%**	F_{PdNP}	F_{AuNP}
**Input**	49	51	0.59	0.41
Area 1	5.05	94.94	0.075	0.925
Area 2	6.06	93.93	0.089	0.911
Area 3	6.11	93.88	0.090	0.910
**Average**	**5.7**	**94.3**	**0.08**	**0.92**
s.d.	0.6	0.6	0.01	0.01
			E.F.	**1.2**

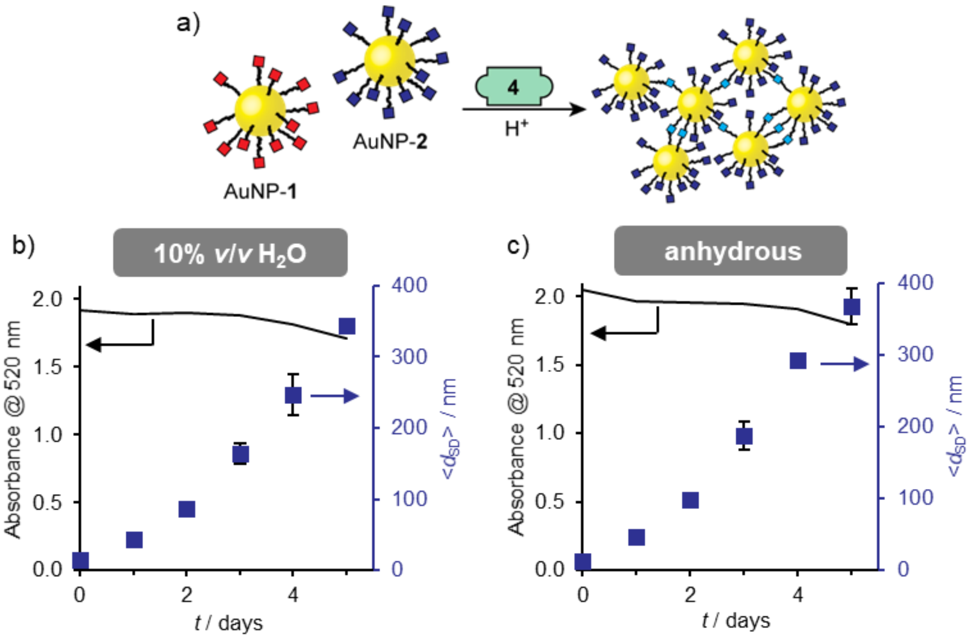

Figure S31. Experiments HLN3 and HLN4. Selective assembly of electrophilic AuNPs from a binary colloidal mixture of AuNP-**1** and AuNP-**2**, equimolar in terms of NP-bound ligands. Variation in solvodynamic diameter (<d_{SD}>, blue symbols) as measured by DLS and absorbance at 520 nm (line) over time after addition of the acid catalyst required for dynamic covalent hydrazone exchange. Assembly conditions (concentrations in terms of molecular species): [AuNP-**1**]$_0$ = 0.075 mM, [AuNP-**2**]$_0$ = 0.075 mM, [**4**]$_0$ = 0.45 mM, [CF_3CO_2H]$_0$ = 20 mM, 9:1 *v*/*v* DMF/H_2O (b) or anhydrous DMF (c).

Experiments LE1–LE3: Electrophilic linker selected assembly

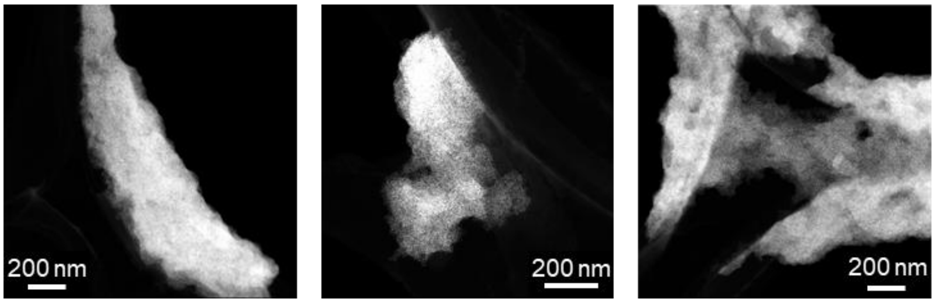

Figure S32. Experiment LE1 representative HAADF images for assemblies produced at day 10 from the electrophilic linker-driven assembly of from a mixture PdNP-**1**, AuNP-**2** and **3** (initial state [**1**]:[**2**]:[**4**] = 1:1:6; PdNP:AuNP = 41:59). EDX spectra were acquired from a region in the centre of each of these areas to give the results reported in Table S10.

Table S10. Experiment LE1 heteromaterial aggregate composition and enrichment factor (*E.F.*) determined by EDX mapping on three distinct sample regions of aggregates produced at day 10 from the electrophilic linker-driven assembly of from a mixture of PdNP-**1**, AuNP-**2** and **3** (initial state [**1**]:[**2**]:[**4**] = 1:1:6; PdNP:AuNP = 41:59).

LE1	Pd atom%	Au atom%	F_{PdNP}	F_{AuNP}
**Input**	26	74	0.41	0.59
Area 1	40.89	59.10	0.582	0.418
Area 2	40.59	59.40	0.579	0.421
Area 3	39.29	60.70	0.566	0.434
**Average**	**40.3**	**59.7**	**0.58**	**0.42**
s.d.	0.9	0.9	0.01	0.01
			E.F.	**–0.28**

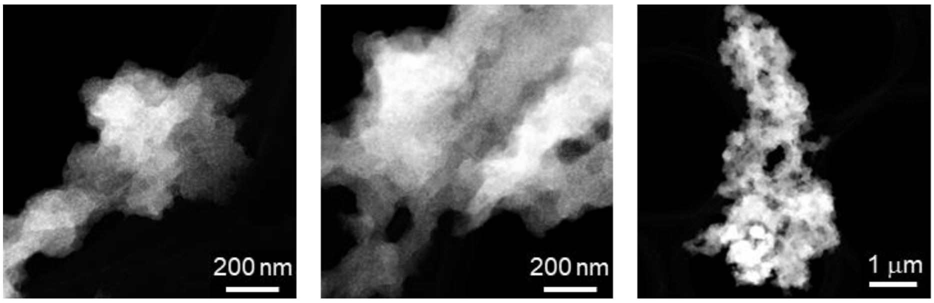

Figure S33. Experiment LE2 representative HAADF images for assemblies produced at day 10 from the during the electrophilic linker-driven assembly of from a mixture PdNP-**1**, AuNP-**2** and **3** (initial state [**1**]:[**2**]:[**4**] = 1:0.7:4.3; PdNP:AuNP = 50:50). EDX spectra were acquired from a region in the centre of each of these areas to give the results reported in Table S11.

Table S11. Experiment LE2 heteromaterial aggregate composition and enrichment factor (*E.F.*) determined by EDX mapping on three distinct sample regions of aggregates produced at day 10 from the electrophilic linker-driven assembly of from a mixture of PdNP-**1**, AuNP-**2** and **3** (initial state [**1**]:[**2**]:[**4**] = 1:0.7:4.3; PdNP:AuNP = 50:50).

LE2	Pd atom%	Au atom%	F_{PdNP}	F_{AuNP}
**Input**	33	67	0.50	0.50
Area 1	77.97	22.02	0.877	0.123
Area 2	78.25	21.74	0.879	0.121
Area 3	74.95	25.04	0.858	0.142
**Average**	**77.1**	**22.9**	**0.87**	**0.13**
s.d.	1.8	1.8	0.01	0.01
			E.F.	**–0.74**

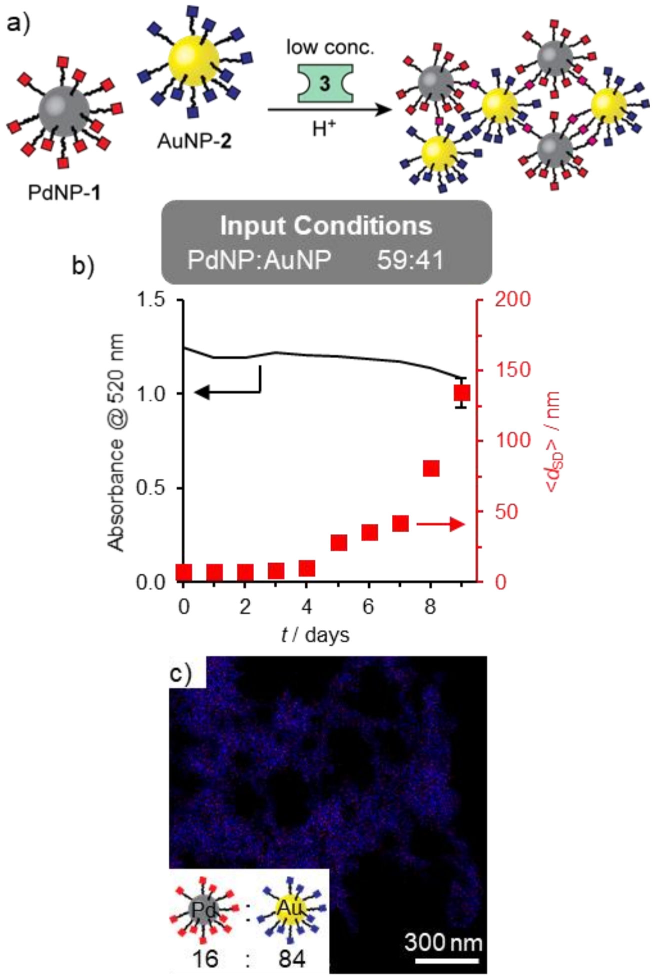

Figure S34. (a) Experiment LE3. Reversed selectivity assembly from a binary colloidal mixture of AuNP-**1** and AuNP-**2**, equimolar in terms of NP-bound ligands using low concentration of linker **3**. (b) Variation in solvodynamic diameter (<d_{SD}>, red symbols) as measured by DLS and absorbance at 520 nm (line) over time after addition of the acid catalyst required for dynamic covalent hydrazone exchange. (c) Representative EDX map of gold-enriched aggregates (blue = Au; red = Pd). Inset: Heteromaterial aggregate composition expressed in terms of ratio of nanoparticles determined by EDX mapping on three distinct sample regions (full results in Table S12, with accompanying HAADF images in Figure S35). Assembly conditions (concentrations in terms of molecular species): [AuNP-**1**(2)]$_0$ = 0.075 mM, [AuNP-**2**(2)]$_0$ = 0.075 mM, (i.e. PdNP : AuNP = 59:41), [**3**]$_0$ = 0.225 mM, [CF_3CO_2H]$_0$ = 20 mM, 9:1 *v*/*v* DMF/H_2O.

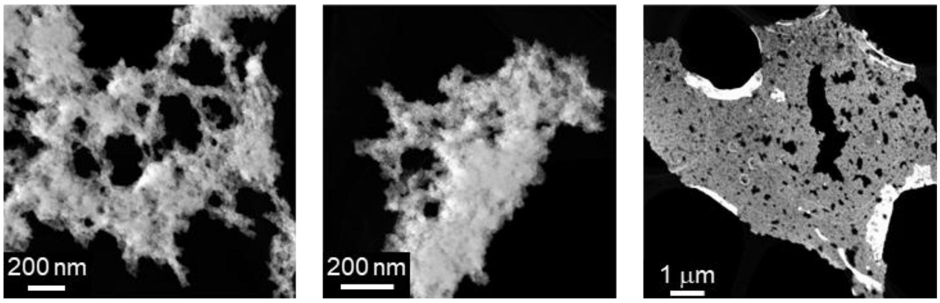

Figure S35. Experiment LE3 representative HAADF images for assemblies produced at day 10 from the during the electrophilic linker-driven assembly of from a mixture PdNP-**1**, AuNP-**2** and **3** (initial state [**1**]:[**2**]:[**4**] = 1:1:3; PdNP:AuNP = 59:41). EDX spectra were acquired from a region in the centre of each of these areas to give the results reported in Table S12.

Table S12. Experiment LE3 heteromaterial aggregate composition and enrichment factor (*E.F.*) determined by EDX mapping on three distinct sample regions of aggregates produced at day 10 from the electrophilic linker-driven assembly of from a mixture of PdNP-**1**, AuNP-**2** and **3** (initial state [**1**]:[**2**]:[**4**] = 1:1:3; PdNP:AuNP = 59:41).

LE3	Pd atom%	Au atom%	F_{PdNP}	F_{AuNP}
**Input**	49	51	0.59	0.41
Area 1	11.96	88.03	0.171	0.829
Area 2	10.78	89.21	0.155	0.845
Area 3	10.85	89.14	0.156	0.844
**Average**	**11.2**	**88.8**	**0.16**	**0.84**
s.d.	0.7	0.7	0.01	0.01
			E.F.	**1.1**

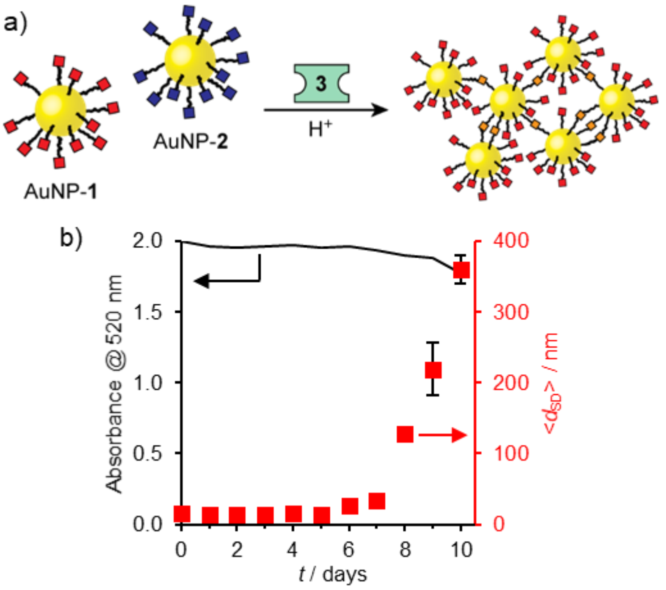

Figure S36. (a) Experiment HLE1. Selective assembly of nucleophilic AuNPs from a binary colloidal mixture of AuNP-**1** and AuNP-**2**, equimolar in terms of NP-bound ligands. (b) Variation in solvodynamic diameter (<d_{SD}>, red symbols) as measured by DLS and absorbance at 520 nm (line) over time after addition of the acid catalyst required for dynamic covalent hydrazone exchange. Assembly conditions (concentrations in terms of molecular species): [AuNP-**1**]$_0$ = 0.075 mM, [AuNP-**2**]$_0$ = 0.075 mM, [**3**]$_0$ = 0.45 mM, [CF_3CO_2H]$_0$ = 20 mM, 9:1 *v*/*v* DMF/H_2O.

Control experiments

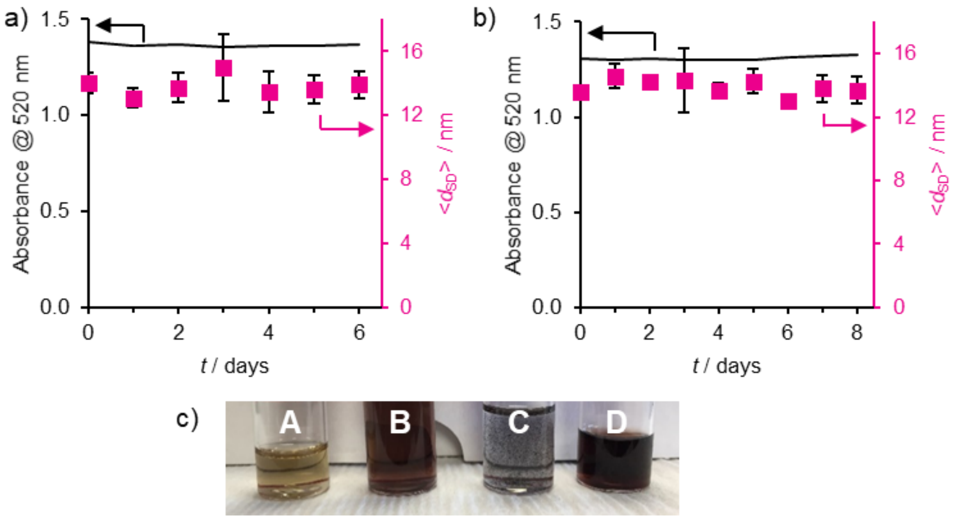

Figure S37. Control experiments for co-assembly from a heteromaterial colloidal mixture of PdNP-**1** and AuNP-**2**. (a, b) Solvodynamic diameter (<d_{SD}>, symbols) as measured by DLS and absorbance at 520 nm evolution over time for mixtures of PdNP-**1** and AuNP-**2** in the absence of the acid catalyst required for dynamic covalent hydrazone exchange. Conditions. (a): **Control 8 (C1, no acid):** [PdNP-**1**]$_0$ = 0.105 mM; [AuNP-**2**]$_0$ = 0.075 mM, [CF_3CO_2H]$_0$ = 0 mM, 9:1 *v*/*v* DMF/H_2O. (b): **Control 7 (C2, no acid):** [PdNP-**1**]$_0$ = 0.075 mM; [AuNP-**2**]$_0$ = 0.075 mM, [CF_3CO_2H]$_0$ = 0 mM, 9:1 *v*/*v* DMF/H_2O. (c) Photographs of (A) PdNP-**1** alone + CF_3CO_2H, (B) PdNP-**1** + AuNP-**2**, no CF_3CO_2H, (C) PdNP-**1** + AuNP-**2** + CF_3CO_2H, (D) AuNP-**2** alone + CF_3CO_2H. Precipitation of colloidally unstable material is only visible in vial C.

6. Kinetic models of selective assembly processes

During each multicomponent assembly process, several reaction pathways, involving numerous reaction substrates located in either bulk solution or nanoparticle-bound environments, are in competition. Nevertheless, even simplified kinetic models that focus on the reactions likely to dominate during the initial stages of each assembly experiment provide useful insight on the overall outcome. Each of these initial reactions generate new nanoparticle-bound and bulk-solution reactive species that can initiate further downstream reaction pathways. Eventually, inter-nanoparticle reactions produce colloidally unstable aggregates, after which precipitation and solution–solid reactions introduce further kinetic complexity. We proposed that the kinetic behavior of each system would be in large part controlled by the reactions of the initial nanoparticle-bound and bulk-solution species.

Making the further simplifying assumption that reactions of nanoparticle-bound species are not affected by neighboring ligand identity (and therefore rates of each particle-bound reaction are independent of the overall evolution of the system) allowed us to make reasonable estimates for the rate constants of each reaction based on our earlier kinetic studies (see below for further details).[5] In turn, this allowed us to probe computationally the effects of parameter variation by simulating the speciation profiles over time under different input conditions. Simulations were run in Copasi 4.33 (Build 246).[8] It should be noted that, given the simplifying approximations and uncertainties in estimated values for some reaction parameters, we interpret these simulations only as representing the qualitative evolution of the assembly processes; absolute values of time and concentration are note expected to be directly comparable to the experimental results.

Selective assembly of electrophilic DCNPs using a nucleophilic linker

The reactions possible during the initial stages of nucleophilic linker-driven assembly are represented by equations (1)–(4) (Scheme S2). Our estimates for the kinetic parameters for reactions (1–4) (Table S13) suggest that the fastest process should be nucleophilic attack of bulk solution bis(hydrazide) **4** on the electrophilic AuNP-**2** (reaction 4). Subsequent reaction of the nucleophilic nanoparticle product AuNP-L**4-NHNH$_2$** with another AuNP-**2** produces the desired homomaterial inter-nanoparticle cross-links. However, either bulk solution or nanoparticle-bound hydrazides can also react with PdNP-**1**, to scavenge the 4-fluorobenzylidine unit (reaction 3 and equivalent reaction between AuNP-L**4-NHNH$_2$** and PdNP-**1**), thus producing nucleophilic PdNP-**NHNH$_2$**. These highly nucleophilic nanoparticle-bound species can subsequently react with AuNP-**2** or AuNP-**CHO** to produce heteromaterial linkages, serving to lower the selectivity of the assembly process. To a first approximation, therefore, selective linker-driven assembly of AuNP-**2** will be achieved by maximizing the concentration of AuNP-L**4-NHNH$_2$**, while minimizing the concentration of PdNP-**NHNH$_2$**. A predictive parameter for assembly selectivity can thus be defined as S_{Au} = [AuNP-L**4-NHNH$_2$**]/[PdNP-**NHNH$_2$**]; maximizing the value of S_{Au} should correspond to higher selectivity in favor of gold-enriched assemblies. Note, we do not include the concentration of the species AuNP-**CHO** in the numerator of S_{Au} as these NP-bound electrophiles can react either with AuNP-L**4-NHNH$_2$** to produce the desired homomaterial assembly, or with PdNP-**NHNH$_2$** to produce heteromaterial links.

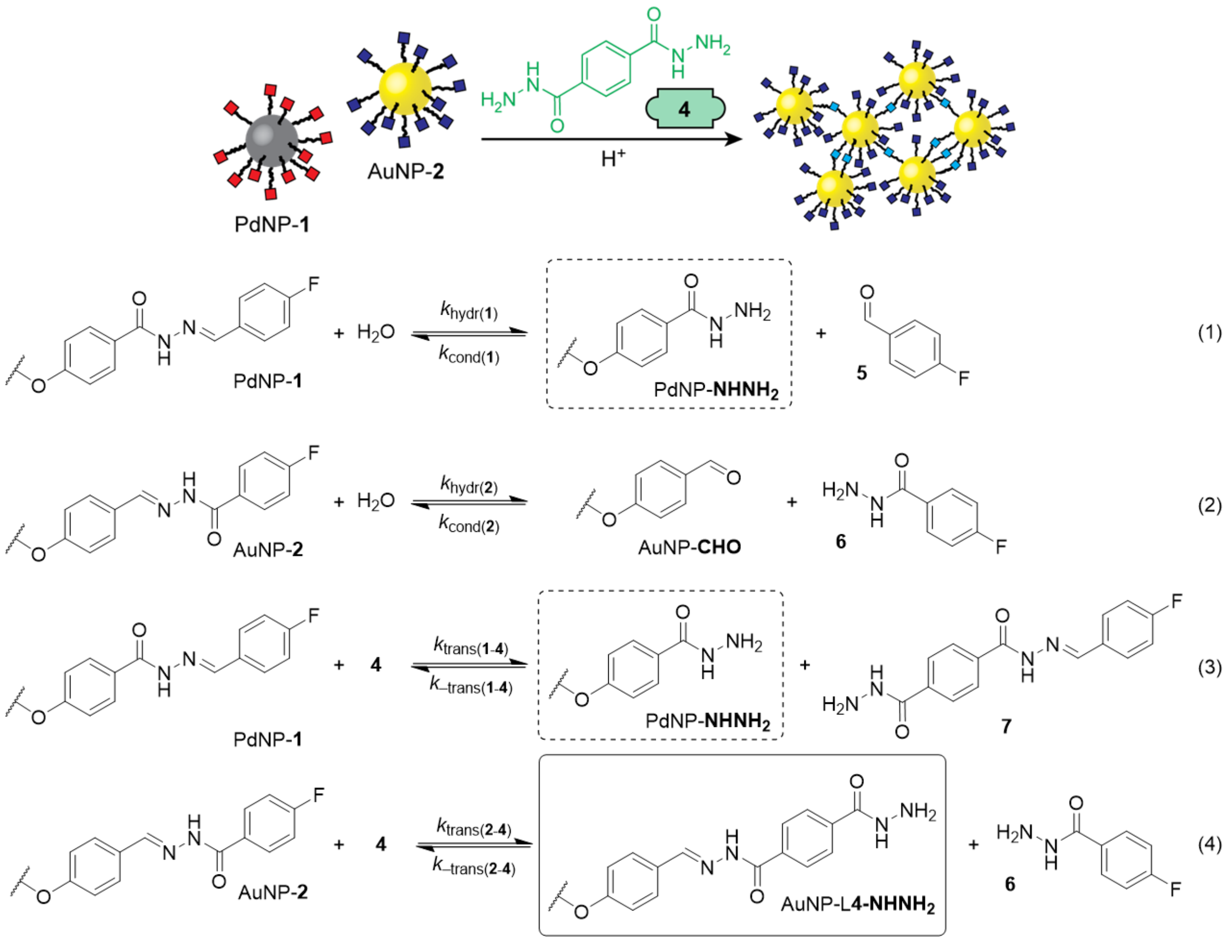

Scheme S2. Reaction pathways available during initial stages of nucleophilic linker-driven assembly from a binary mixture of nucleophilic PdNP-**1** and electrophilic AuNP-**2**. The dominant role of each nanoparticle-bound intermediate in favoring (solid boxes) or disfavoring (dashed boxes) selectivity is indicated. Rate constants for hydrolysis (k_{hydr}), condensation (k_{cond}) and transimination (k_{trans}) reactions are labelled; estimated values are given in Table S13.

Table S13. Estimated rate constants for reaction processes dominating during the initial stages of nucleophilic linker-driven assembly from a mixture of nucleophilic PdNP-**1** and electrophilic AuNP-**2** (Scheme S2). The measurement and estimation of rate constants is discussed in the following section.

Reaction	Parameter	Value / mM^{-1} h^{-1}
1	$k_{hydr(\mathbf{1})}$	4×10^{-6}
1	$k_{cond(\mathbf{1})}$	0.9
2	$k_{hydr(\mathbf{2})}$	1×10^{-5}
2	$k_{cond(\mathbf{2})}$	0.7
3	$k_{trans(\mathbf{1-4})}$	0.1
3	$k_{-trans(\mathbf{1-4})}$	0.2
4	$k_{trans(\mathbf{2-4})}$	1
4	$k_{-trans(\mathbf{2-4})}$	1

Building a kinetic model using reactions (1)–(4) and our estimated rate constants (Table S13) allowed us to simulate the speciation and evolution of S_{Au} over time (Figure S38). The simulations reveal that selectivity is maximal at the very start of the process, rapidly decreasing as the concentration of PdNP-**NHNH₂** increases. Thus, the maximum in [AuNP-L**4**-**NHNH₂**] during the first few hours of the simulation is key to achieving high kinetic selectivity, indicating that a balance must be struck between aggregate yield and selectivity. In the absence of water, reactions (1) and (2) are shut down, with the consequence that absolute levels of selectivity are increased and extending the timeframe for which homomaterial-favoring species dominate (Figure S38d–f).

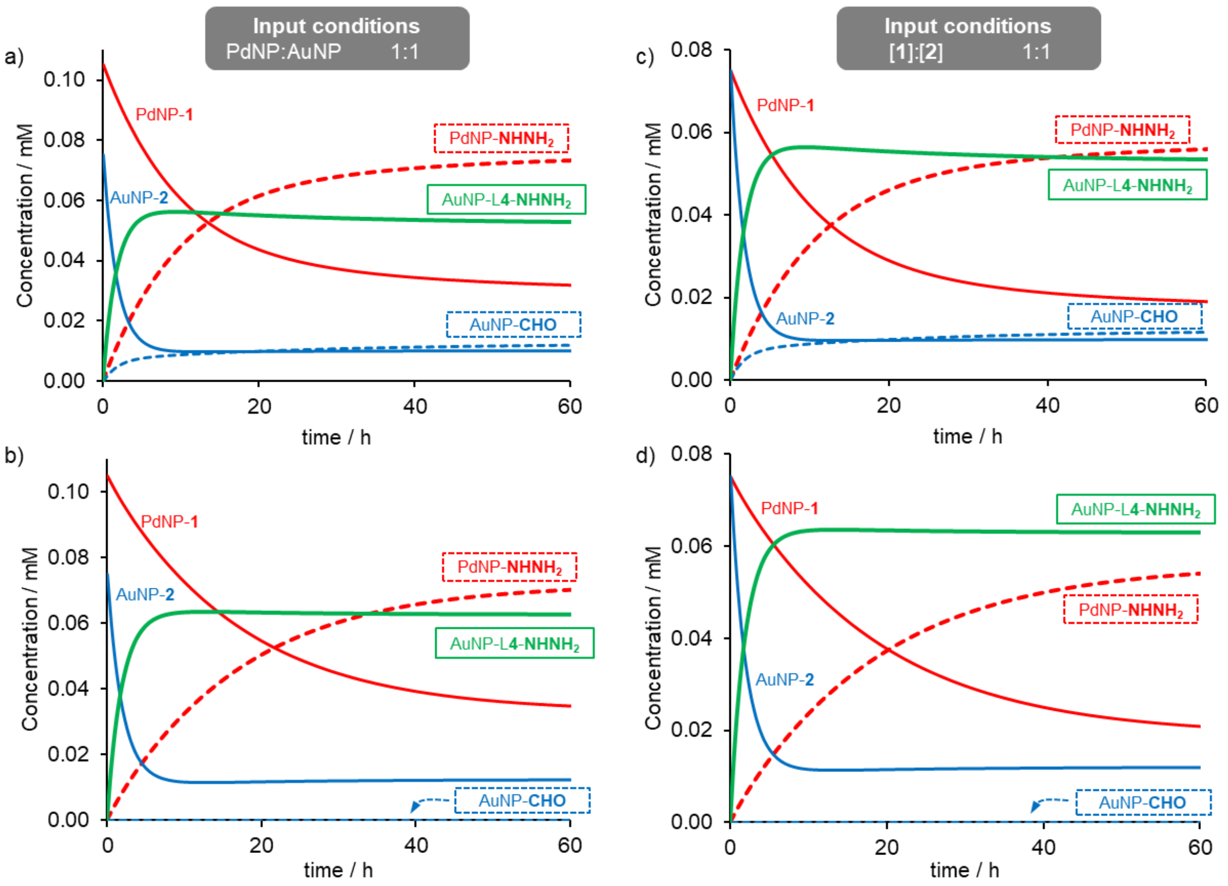

Figure S38. Simulated speciation profiles describing the evolution of concentration for nanoparticle-bound species for nucleophilic linker-driven assembly from a binary mixture of nucleophilic PdNP-**1** and electrophilic AuNP-**2** starting at 1:1 in terms of particles (a, b) or 1:1 in terms of NP-bound ligands (d, e). Species that contribute to favoring selective assembly are plotted with heavy solid lines; species that are deleterious to selective assembly are plotted with heavy dashed lines. All concentrations are given in terms of molecular species. Conditions (a, b): [PdNP-**1**]$_0$ = 0.105 mM, [AuNP-**2**]$_0$ = 0.075 mM, [**4**]$_0$ = 0.45 mM (6 mol. equiv.), (a) [H_2O]$_0$ = 5551 mM (10% *v*/*v*), (b) [H_2O]$_0$ = 0 mM. Conditions (c, d): [PdNP-**1**]$_0$ = 0.075 mM, [AuNP-**2**]$_0$ = 0.075 mM, [**4**]$_0$ = 0.45 mM (6 mol. equiv.), (c) [H_2O]$_0$ = 5551 mM (10% *v*/*v*), (d) [H_2O]$_0$ = 0 mM. Corresponding plots of the evolution of S_{Au} over time are shown in Figure 6a, b.

Simulating the influence of linker concentration revealed that both the rate of production and absolute maximum concentration of key species AuNP-L**4**-**NHNH$_2$** increase with increased [**4**]$_0$ (Figure S39a, solid lines). Increasing linker concentration also affects the rate of generation of PdNP-**NHNH$_2$** but less strongly (Figure S39a, dashed lines). The consequence for the overall selectivity (Figure S39b) is that higher linker concentrations correspond to higher absolute selectivity ($t \rightarrow 0$) and steady state selectivity ($t \rightarrow \infty$), but also cause more rapid erosion of selectivity at intermediate time points. Crucially, therefore, the optimum linker concentration depends on the time point at which the aggregate will be probed (Figure S39b, inset). This is consistent with our observation that the highest experimental selectivity was observed with 3 molar equivalents of linker under the input conditions of experiment LN5 (main text Figure 8 and Table S2).

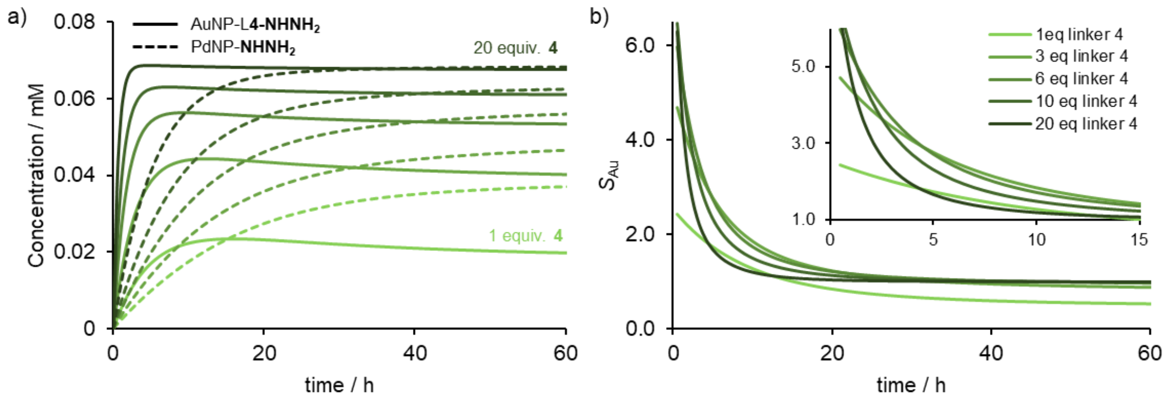

Figure S39. Effect of initial linker concentration on the selectivity for AuNPs under nucleophilic linker-driven assembly from a binary mixture of of nucleophilic PdNP-**1** and electrophilic AuNP-**2** (input conditions 1:1 in terms of NP-bound ligands, water present). (a) Evolution of concentrations for key species that favor (AuNP-L**4**-**NHNH$_2$**, solid lines) and disfavor (PdNP-**NHNH$_2$**, dashed lines) selectivity for AuNP assembly. (b) Evolution of gold selectivity parameter S_{Au} for assembly in the presence of different molar equivalents of linker **4**. Inset: zoom on time period over which maximum selectivity does not correlate with initial linker concentration. All concentrations are in terms of molecular species. Conditions: [PdNP-**1**]$_0$ = [AuNP-**2**]$_0$ = 0.075 mM, [H_2O] = 5551 mM (10% *v*/*v*), [**4**]$_0$ = 0.075 mM (1 mol. equiv.), 0.075 mM (6 mol. equiv.), 0.45 mM (6 mol. equiv.), 0.75 mM (10 mol. equiv.), 1.5 mM (20 mol. equiv.).

Selective assembly of nucleophilic DCNPs using an electrophilic linker

Selective assembly of nucleophilic DCNPs (Scheme S3) first requires hydrolysis of PdNP-**1** to reveal nanoparticle-bound nucleophile PdNP-**NHNH$_2$** (reaction 1). Although an essential intermediate en route to the necessary linker-capped PdNP–L**3**–**CHO** (reaction 5), the key nanoparticle-bound nucleophiles can also initiate pathways that are deleterious to assembly selectivity, via condensation with AuNP-**CHO** (reaction 6), or direct transamination with AuNP-**2** (reaction 10). As we have no experimental system that is able to provide a quantitative measure of reaction rate between two nanoparticle-bound species, kinetic parameters for these reactions are poorly known. The latter reaction – a transimination between two nanoparticle-bound species – can be predicted to be relatively slow; however, we observe that hydrazide–aldehyde condensation reactions are minimally affected by surface confinement of only one component, so we must assume that the analogous nanoparticle–nanoparticle analogues (e.g. reaction 6) may be kinetically significant. Conversely, the formation of inter-particle linkages can be expected to be highly cooperative, so hydrolysis of interparticle linkages is likely to be considerably kinetically inhibited – even when one linkage is cleaved, this is unlikely to result in a change to the nanoparticle aggregation state.

Based on the considerations above, the concentration of PdNP-**NHNH$_2$** is not an adequate predictor of assembly selectivity. Therefore, we must consider a second level of reactions involving the initially generated intermediates. Thus, including reaction 6, as discussed, and reaction 7 – scavenging of the linker by the released nucleophile **6**. The resultant monotopic molecular aldehyde **6**-L**3**-**CHO** can in-turn cap the nucleophilic DCNPs PdNP**-NHNH$_2$** to generate capped PdNP-L**3**-**6** (reaction 11). This 'dead-end' intermediate can also be produced by direct reaction of molecular nucleophile **6** with key intermediate PdNP-L**3**-**CHO** (reaction 9); so, both of these processes reduce the concentration of key reactive nanoparticle intermediates. A transimination mode of attack between nucleophilic PdNP-**NHNH$_2$** and either linker-activated DCNPs PdNP-L**3**-**6** or monotopic linker **6**-L**3**-**CHO** is also available, giving alternative pathways to Pd-selective NP aggregates PdNP-L**3**-PdNP or linker-activated PdNP-L**3**-**CHO**, respectively (Reactions 13 and 12). It can be predicted that each of these transamination processes is significantly slower than the alternative hydrazone condensation mode of attack at the highly reactive aldehyde site in each case.

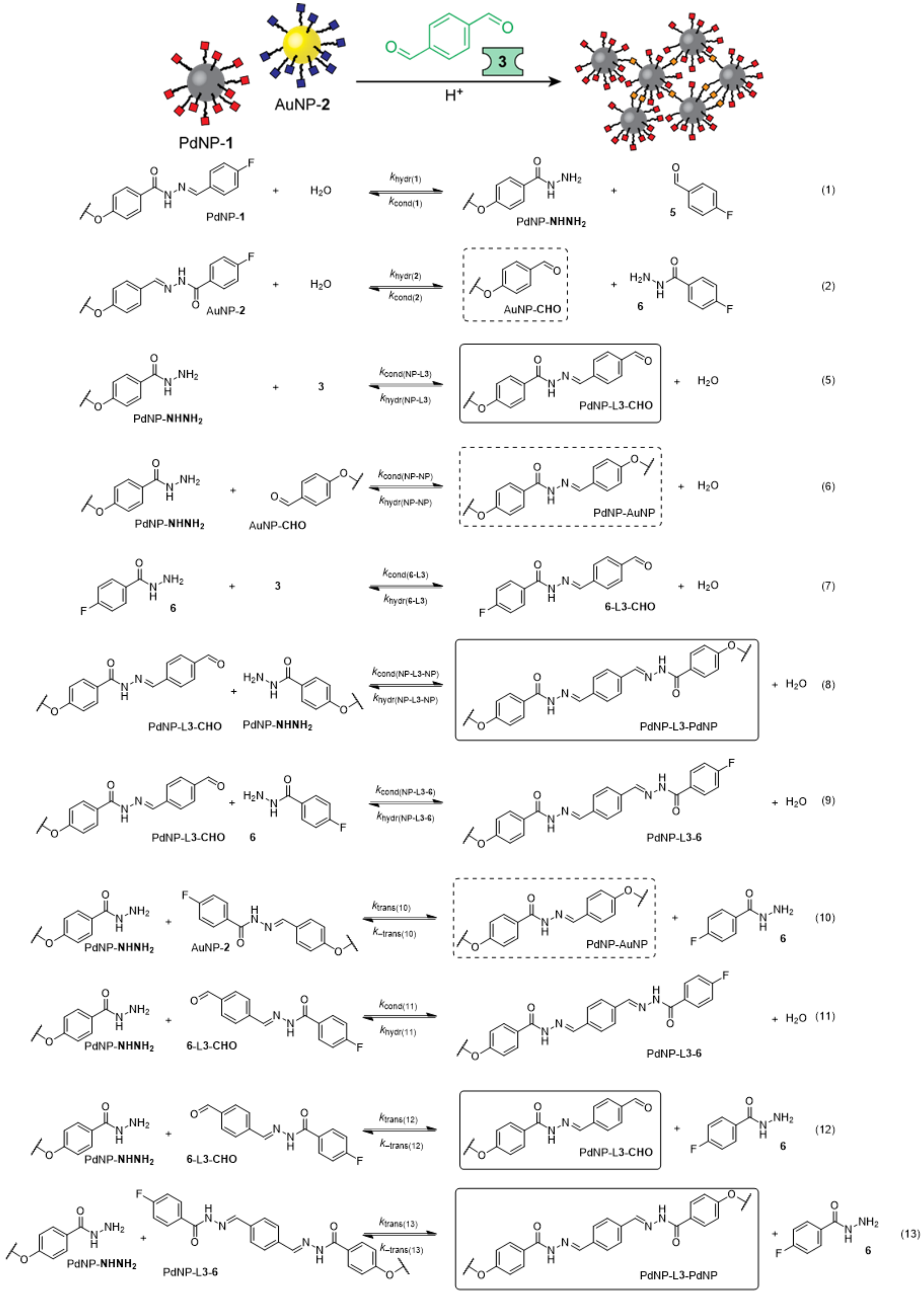

Scheme S3. Reaction pathways available during electrophilic linker-driven assembly from a mixture of nucleophilic PdNP-**1** and electrophilic AuNP-**2**. The dominant role of nanoparticle-bound intermediates in favoring (solid boxes) or disfavoring (dashed boxes) selectivity is indicated. Rate constants for hydrolysis (k_{hydr}), condensation (k_{cond}) and transimination (k_{trans}) reactions are labelled; estimated values are given in Table S14.

Table S14. Estimated rate constants for reaction processes dominating during electrophilic linker-driven assembly from a mixture of nucleophilic PdNP-**1** and electrophilic AuNP-**2** (Scheme S3). The measurement and estimation of rate constants is discussed in the following section.

Reaction	Parameter	Value / mM^{-1} h^{-1}
1	$k_{\mathrm{hydr}(\mathbf{1})}$	4 × 10^{-6}
1	$k_{\mathrm{cond}(\mathbf{1})}$	0.9
2	$k_{\mathrm{hydr}(\mathbf{2})}$	1 × 10^{-5}
2	$k_{\mathrm{cond}(\mathbf{2})}$	0.7
5	$k_{\mathrm{cond(NP\text{-}L}\mathbf{3})}$	0.9
5	$k_{\mathrm{hydr(NP\text{-}L}\mathbf{3})}$	4 × 10^{-6}
6	$k_{\mathrm{cond(NP\text{-}NP)}}$	0.4
6	$k_{\mathrm{hydr(NP\text{-}NP)}}$	1 × 10^{-6}
7	$k_{\mathrm{cond}(\mathbf{6}\text{-}\mathrm{L}\mathbf{3})}$	1
7	$k_{\mathrm{hydr}(\mathbf{6}\text{-}\mathrm{L}\mathbf{3})}$	9 × 10^{-6}
8	$k_{\mathrm{cond(NP\text{-}L}\mathbf{3}\mathrm{\text{-}NP)}}$	0.8
8	$k_{\mathrm{hydr(NP\text{-}L}\mathbf{3}\mathrm{\text{-}NP)}}$	1 × 10^{-6}
9	$k_{\mathrm{cond(NP\text{-}L}\mathbf{3}\text{-}\mathbf{6})}$	1
9	$k_{\mathrm{hydr(NP\text{-}L}\mathbf{3}\text{-}\mathbf{6})}$	4 × 10^{-6}
10	$k_{\mathrm{trans}(10)}$	0.5
10	$k_{-\mathrm{trans}(10)}$	0.9
11	$k_{\mathrm{cond}(11)}$	0.9
11	$k_{\mathrm{hydr}(11)}$	4 × 10^{-6}
12	$k_{\mathrm{trans}(12)}$	0.2
12	$k_{-\mathrm{trans}(12)}$	0.1
13	$k_{\mathrm{trans}(13)}$	0.2
13	$k_{-\mathrm{trans}(13)}$	0.06

In this model, the linker molecule cannot react with the nanoparticles in their starting state; the action of water is first required to produce a nanoparticle-bound nucleophile. Thus, the influence of both water and the concentration of linker on the assembly process should be expected to be quite different to the selective assembly of electrophilic DCNPs considered above. Selective assembly of nucleophilic DCNPs will be favored by maximizing the concentration of linker-activated PdNP-L**3-CHO**, which should react in a relatively fast nanoparticle–nanoparticle condensation reaction with PdNP-**NHNH$_2$** to produce homomaterial assemblies. Meanwhile, minimizing the concentration of AuNP-**CHO**, which can undergo a condensation with PdNP-**NHNH$_2$** to give heteromaterial assemblies, should also be key to selectivity. One intrinsic factor favoring the desired selectivity should be the faster reaction of electron-poor PdNP-L**3**-**CHO** in reaction 8 compared to electron-rich nanoparticle-bound electrophile AuNP-**CHO** in reaction 6.

A measure of selectivity for this assembly process should be provided by the number of homomaterial versus heteromaterial inter-particle linkages: S_{Pd} = [PdNP-L**3**-PdNP]/[PdNP-AuNP] (main text Figure 7). However, it should be noted that this parameter is derived from a model that includes poorly known interparticle reaction rates. As a measure of the extent of aggregation, we also considered the number of interparticle linkages as a proportion of the total number of nanoparticle-bound reactive units (%NP-NP link).

$$\%\text{NP-NP Link}=\frac{2[\text{PdNP-L}\mathbf{3}\text{-PdNP}]+2[\text{PdNP-AuNP}]}{[\text{AuNP-}\mathbf{2}]_0+[\text{PdNP-}\mathbf{1}]_0}\times 100$$

In contrast to the nucleophilic linker-driven assembly of electrophilic DCNPs, simulations reveal that selectivity for nucleophilic DCNPs in the presence of an electrophilic linker reaches a maximal steady state at long time periods (main text Figure 7). Furthermore, the small number of interparticle linkages predicted at the steady state (Figure S41) is in line with our experimental observations that a large proportion of material remains in solution.

Examining the evolution of activated nanoparticle species over time, it is apparent that as well as the hydrolyzed electrophilic AuNP-**CHO**, linker-activated and linker-capped nucleophilic DCNP species PdNP-L**3**-**CHO** and PdNP-L**3**-**6** dominate in solution at later time points (Figure S40a, c). Increasing the rate of PdNP-**1** hydrolysis in an attempt to produce more nucleophiles that may capture PdNP-L**3**-**CHO**, however, will concurrently increase the formation of heteromaterial linkages through reaction with AuNP-**CHO**, as well as generating higher concentrations of molecular capping agent **6**, thus producing more dead-end PdNP-L**3**-**6**. Therefore, the parameter most likely to positively influence aggregate selectivity is the concentration of linker **3**. The simulations predict that employing higher relative concentrations of **3** increases selectivity (main text Figure 7), but at the expense of further reducing aggregate yield (Figure S41), suggesting that moderate molar excesses of linker are optimal. It is notable that at low concentrations of linker **3**, $S_{Pd} < 1$ at all time points (Figure 7a), corresponding to more heteromaterial than homomaterial linkages. This is consistent with our experimental results where the experiment with the lowest linker concentration (LE3) showed no selectivity for assembly of PdNP.

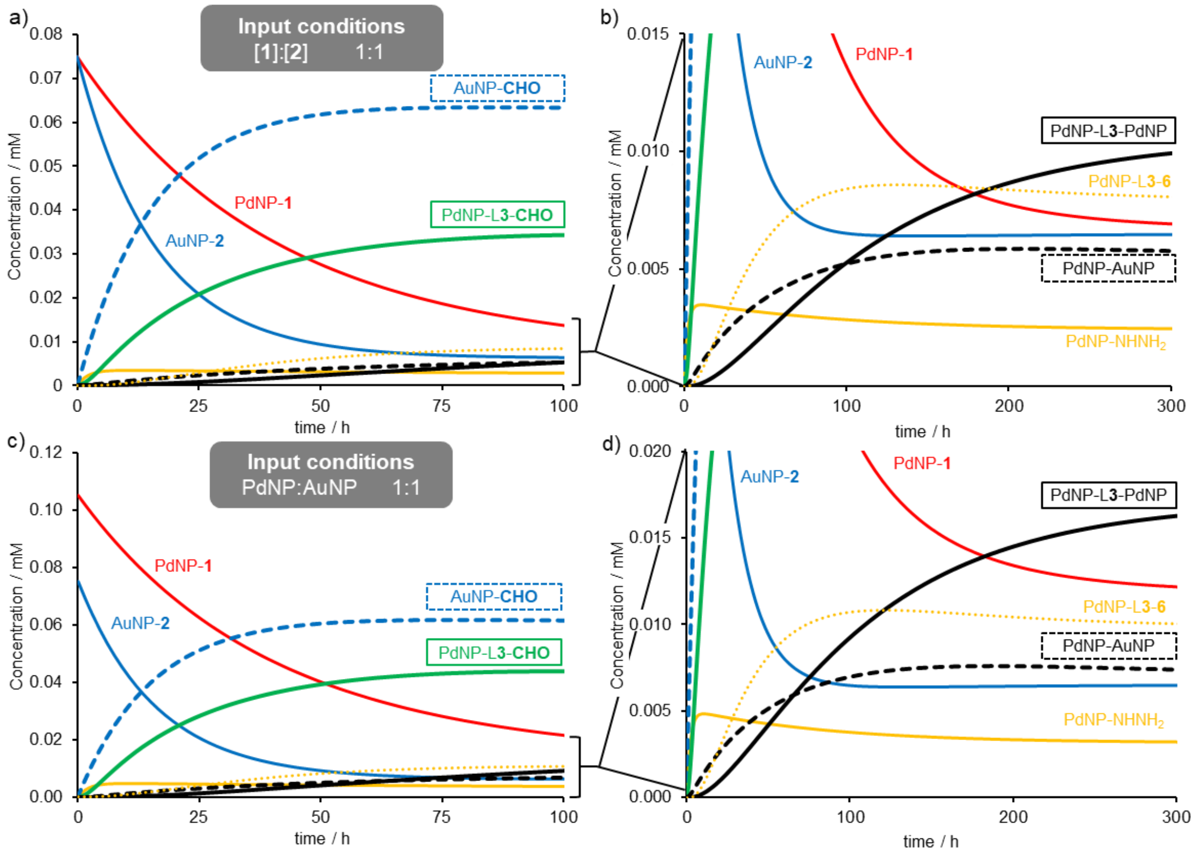

Figure S40. Simulated speciation profiles describing the evolution of concentrations of nanoparticle-bound species during electrophilic linker-driven assembly from a binary mixture of nucleophilic PdNP-**1** and electrophilic AuNP-**2** starting at 1:1 in terms of NP-bound ligands (a–b) or 1:1 in terms of nanoparticles (c–d). Species that contribute to favoring selective assembly are plotted with heavy solid lines; species that are deleterious to selective assembly are plotted with heavy dashed lines. All concentrations are given in terms of molecular species. Conditions (a–b): [PdNP-**1**]$_0$ = 0.075 mM, [AuNP-**2**]$_0$ = 0.075 mM, [**3**]$_0$ = 0.45 mM (6 mol. equiv.) $[H_2O]_0$ = 5551 mM (10% *v*/*v*); (c–d): [PdNP-**1**]$_0$ = 0.105 mM, [AuNP-**2**]$_0$ = 0.075 mM, [**3**]$_0$ = 0.45 mM (4.3 mol. equiv.), $[H_2O]_0$ = 5551 mM (10% *v*/*v*).

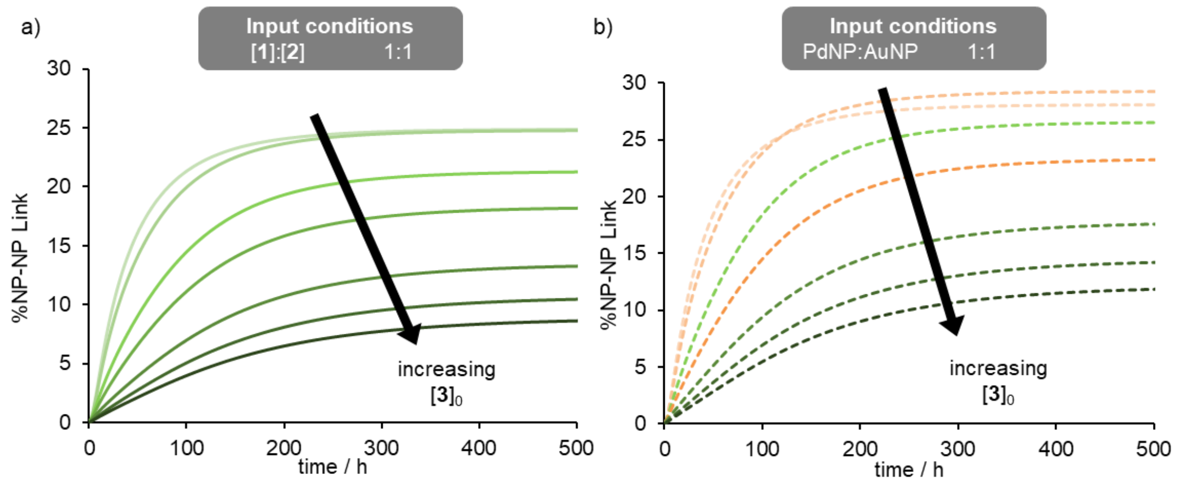

Figure S41. Effect of initial linker concentration on the proportion of linked nanoparticles over time for input conditions that are equimolar in terms of nanoparticle-bound hydrazones (a) or equimolar in terms of nanoparticles (b) for concentrations of electrophilic linker **3** ranging (light green to dark green): $[\mathbf{3}]_0$ = 0.075 mM, 0.15 mM, 0.45 mM, 0.75 mM, 1.50 mM, 2.25 mM, 3.00 mM). a) $[\mathbf{1}]_0 = [\mathbf{2}]_0$ = 0.075 mM; b) $[\mathbf{1}]_0$ = 0.105 mM, $[\mathbf{2}]_0$ = 0.075 mM. %NP-NP Link = ({2[PdNP-L**3**-PdNP] + 2[PdNP-AuNP]} / {[AuNP-**2**]$_0$ + [PdNP-**1**]$_0$}) × 100).

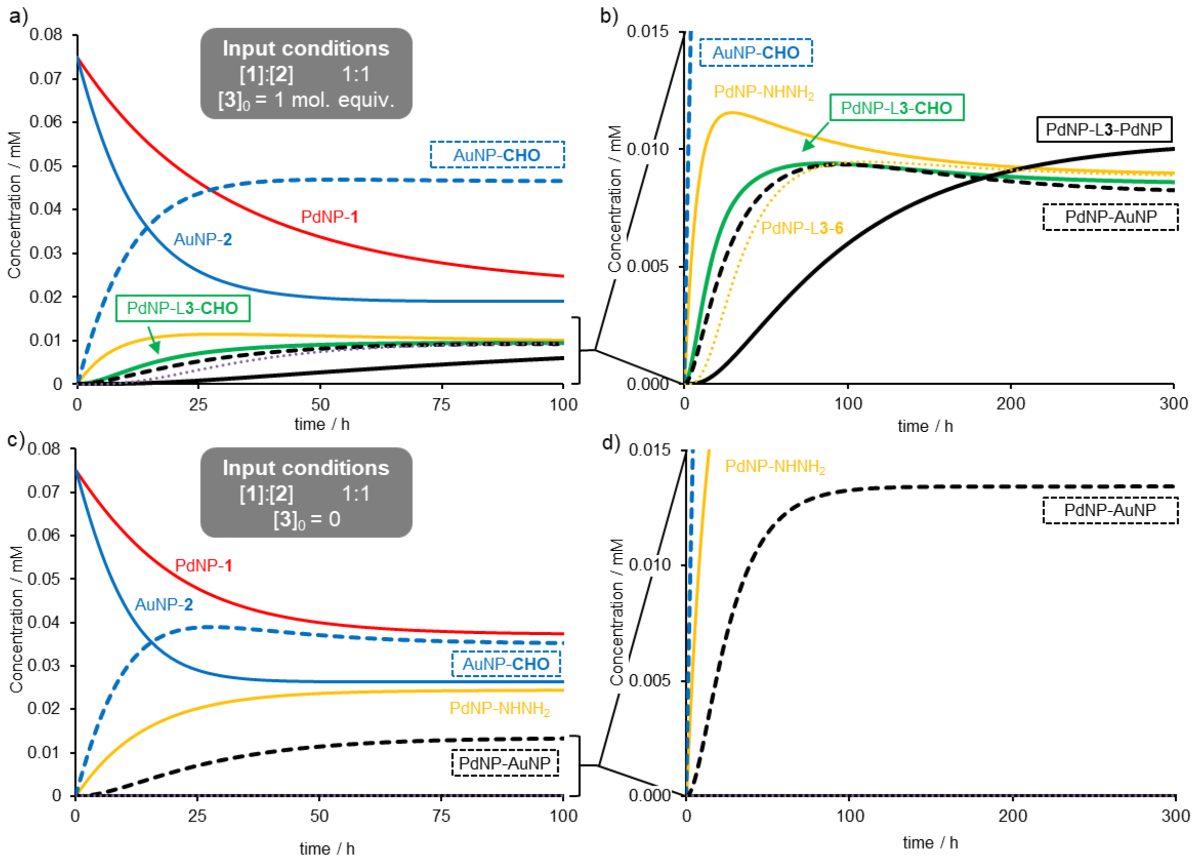

Figure S42. Simulated speciation profiles describing the evolution of concentrations of nanoparticle-bound species during assembly from a binary mixture of nucleophilic PdNP-**1** and electrophilic AuNP-**2** at low concentration of electrophilic linker (a–b) and in the absence of any linker (c–d), starting at 1:1 in terms of NP-bound ligands. Species that contribute to favoring selective assembly of PdNPs are plotted with heavy solid lines; species that are deleterious to selective assembly of PdNPs are plotted with heavy dashed lines. All concentrations are given in terms of molecular species. Conditions: [PdNP-**1**]$_0$ = 0.075 mM, [AuNP-**2**]$_0$ = 0.075 mM, $[H_2O]_0$ = 5551 mM (10% *v*/*v*); (a–b): $[\mathbf{3}]_0$ = 0.075 mM (1 mol. equiv.); (c–d): $[\mathbf{3}]_0$ = 0 mM.

Parameter estimation for kinetic models

Rate constants used in the kinetic models were estimated by direct experimental measurement where possible or by extrapolation from values measured using monotopic (i.e. non-aggregating) modifier units as described below. All measurments were made on AuNP cores. In recogntion of the uncertainty in the extrapolated values, we only consider values to an accurarcy of 1 significant figure. Experimental measurements were made by reaction tracking using ^{19}F NMR as previously reported for AuNP-**1** and AuNP-**2**.[5]

Condensations and hydrolyses involving molecular fragments

Rate constants were estimated (Tables S15 and S16) from previously reported experimental measurements (Scheme S4).[5] Based on our observations of these and analogous reactions on molecular substrates, two trends were applied to estimate rate constants for reactions that could not be measured directly:

- Hydrolysis rates depend strongly on the electronic nature of the benzylidine portion and are only moderately influenced by the electronic nature of the leaving group. Nanoparticle attachment via the bezylidine end inhibits hydrolysis by a factor of ca. ×0.5 and via the hydrazide fragment by a factor of ca. ×0.8.[5]
- Condensation rates strongly depend on the electronic nature of the aldehyde and are moderately influenced by the electronic nature of the hydrazide. Condensations are minimally affected by the regiochemistry of the reactive site with typical surface inhibition factors ca. ×0.9 of the solution phase value.[5]

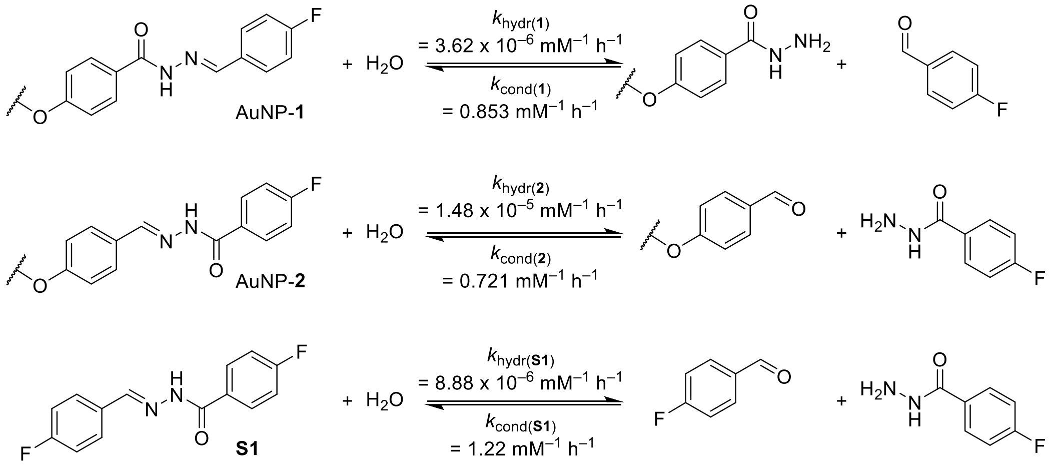

Scheme S4. Experimentally determined rate constants used for estimating values for hydrolyses and condensations involving at least one molecular fragment. Values were determined as previously described by tracking reactions using ^{19}F{^{1}H} NMR spectroscopy then fitting to a bimolecular reversible rate equation.[5] Conditions: CF_3CO_2H (20 mM), D_2O/DMF (10% *v*/*v*).

Table S15. Estimation of rate constants for hydrolyses involving at least one molecular fragment.

Parameter	Reaction Type	Parameter estimation	Value / mM^{-1} h^{-1}
$k_{hydr(\mathbf{1})}$	Hydrazide: NP-bound, electron rich; Benzylidine: electron poor	Experimental measurement	4 × 10^{-6}
$k_{hydr(\mathbf{2})}$	Hydrazide: electron rich Benzylidine: NP-bound; electron rich	Experimental measurement	1 × 10^{-5}
$k_{hydr(NP\text{-}L\mathbf{3})}$	Hydrazide: NP-bound, electron rich; Benzylidine: electron poor	Estimate = $k_{hydr(\mathbf{1})}$	4 × 10^{-6}
$k_{hydr(\mathbf{6}\text{-}L\mathbf{3})}$	Hydrazide: electron poor; Benzylidine: electron poor	Estimate = $k_{hydr(\mathbf{S1})}$	9 × 10^{-6}
$k_{hydr(NP\text{-}L\mathbf{3}\text{-}\mathbf{6})}$	Hydrazide: electron poor; Benzylidine: NP-bound; electron poor	Estimate = 0.5× $k_{hydr(\mathbf{S1})}$	4 × 10^{-6}
$k_{hydr(11)}$	Hydrazide: NP-bound, electron rich; Benzylidine: electron poor	Estimate = $k_{hydr(\mathbf{1})}$	4 × 10^{-6}

Table S16. Estimation of rate constants for condensations involving at least one molecular fragment.

Parameter	Reaction Type	Parameter estimation	Value / mM^{-1} h^{-1}
$k_{cond(\mathbf{1})}$	Hydrazide: NP-bound; electron rich; Aldehyde: molecular; electron poor	Experimental measurement	0.9
$k_{cond(\mathbf{2})}$	Hydrazide: molecular; electron poor Aldehyde: NP-bound; electron rich	Experimental measurement	0.7
$k_{cond(NP\text{-}L\mathbf{3})}$	Hydrazide: NP-bound; electron rich; Aldehyde: molecular; electron poor	Estimate = $k_{cond(\mathbf{1})}$	0.9
$k_{cond(\mathbf{6}\text{-}L\mathbf{3})}$	Hydrazide: molecular; electron poor; Aldehyde: molecular; electron poor	Estimate = $k_{cond(\mathbf{S1})}$	1
$k_{cond(NP\text{-}L\mathbf{3}\text{-}\mathbf{6})}$	Hydrazide: NP-bound electron poor; Aldehyde: molecular; electron poor	Estimate = 0.9× $k_{cond(\mathbf{S1})}$	1
$k_{cond(11)}$	Hydrazide: NP-bound; electron rich; Aldehyde: molecular; electron poor	Estimate = $k_{cond(\mathbf{1})}$	0.9

Transimination reactions involving molecular fragments

Reaction rates for transiminations between one nanoparticle-bound and one bulk solution component were estimated (Table S17) based on values measured for reactions with monotopic modifiers (Scheme S5). Observations of analogous reactions on molecular substrates evidenced that the benzylidine portion has the most significnat effect on reactivity. Therefore, where a direct experimental comparison to a reaction in the model was not available, the reaction that most closely reflected the benzylidine portion was chosen and the rate constant adjusted to account for the electronic nature of either the hydrazide nucleophile or leaving group according to the trends:

- Electron-poor nuleophiles react faster than electron-rich by a factor of ca. 1.4;
- Electron-rich leaving groups react faster than electron-poor by a factor of ca. 1.2.

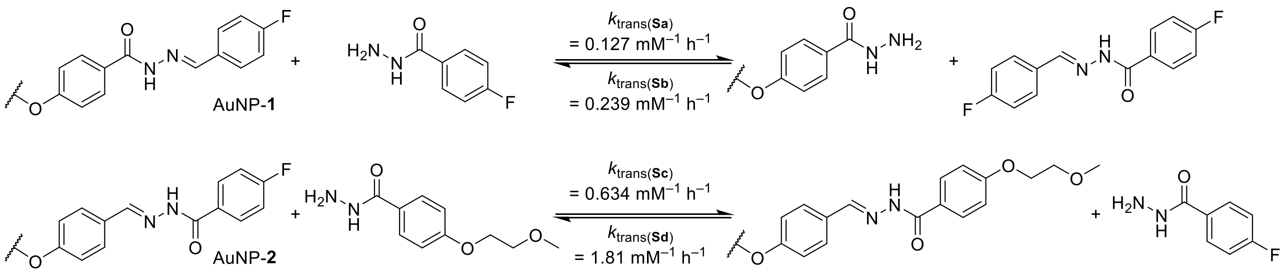

Scheme S5. Experimentally determined rate constants used for estimating values for transiminations involving at least one molecular fragment. Values were determined as previously described by tracking reactions using ^{19}F{^{1}H} NMR spectroscopy then fitting to a bimolecular reversible rate equation.[5] Conditions: CF_3CO_2H (20 mM), DMF.

Table S17. Estimation of rate constants for transiminations involving at least one molecular fragment.

Parameter	Reaction Type	Parameter estimation	Value / mM^{-1} h^{-1}
$k_{trans(\mathbf{1-4})}$	Hydrazide: molecular; electron poor<br>Benzylidine: NP-bound; electron poor<br>Leaving group: electron rich	Estimate = $k_{trans(\mathbf{Sa})}$	0.1
$k_{-trans(\mathbf{1-4})}$	Hydrazide: NP-bound; electron rich<br>Benzylidine: molecular; electron poor<br>Leaving group: electron poor	Estimate = $k_{trans(\mathbf{Sb})}$	0.2
$k_{trans(\mathbf{2-4})}$	Hydrazide: molecular; electron poor<br>Benzylidine: NP-bound; electron rich<br>Leaving group: electron poor	Estimate<br>≈ 1.4× $k_{trans(\mathbf{Sc})}$ ≈ 0.8× $k_{trans(\mathbf{Sd})}$	1
$k_{-trans(\mathbf{2-4})}$	Hydrazide: molecular; electron poor<br>Benzylidine: NP-bound; electron rich<br>Leaving group: electron poor	Estimate<br>≈ 1.4× $k_{trans(\mathbf{Sc})}$ ≈ 0.8× $k_{trans(\mathbf{Sd})}$	1
$k_{trans(12)}$	Hydrazide: NP-bound; electron rich<br>Hydrazone: molecular; electron poor<br>Leaving group: electron poor	Estimate = $k_{trans(\mathbf{Sb})}$	0.2
$k_{-trans(12)}$	Hydrazide: molecular; electron poor<br>Hydrazone: NP-bound; electron poor<br>Leaving group: electron rich	Estimate = $k_{trans(\mathbf{Sa})}$	0.1

Reactions involving nanoparticle–nanoparticle linkages

The rate constants for reactions involving hydrazones that link two nanoparticles are the least well known as we have not been able to measure these directly. The following principles were applied to arrive at reasonable estimates for these reaction rates.

- Similar electronic trends are followed as observed in reactions involving at least one molecular component or on molecular substrates.
- Condensation reactions are minimallly affected by the regiochemistry of the nucleophile. A conservative factor of ×0.9 was applied to account for the diffference between reactions involving two NP-bound compontents compared to only one NP-bound component.
- Rates of hydrolysis at interparticle linkages will be the slowest reactions in the system. The rates of hydrolysis for NP-bound hydrazones are in the range $4–10 \times 10^{-6}$ mM^{-1} h^{-1} (Table S15), so we set a conservative estimate for hydolysis of interparticle linkages as 1×10^{-6} mM^{-1} h^{-1}. It is likely that as aggregates grow, the rates of these reactions become increasingly inhibited. We found that further decreasing this value did not have a significant effect on the qualitative conclusions from the simulations.
- Comparing transiminations involving NP-bound hydrazides at molecular substrates show inhibitions of ca. ×0.8 compaerd to the all-molecular reactions. The same factor was applied to account for a NP-bound hydrazide attacking a NP-bound substrate.
- Transimination reactions at interparticle linkages are likely to be significantly inhibited, but should remain faster than the corresponding hydrolysis processes (cf. Tables S15 and S17). A factror of ×0.5 was applied to the equivalent reaction rate at a NP-bound substrate to account for this regiochemistry.

Table S18. Estimation of rate constants for transiminations involving at least one molecular fragment.

Parameter	Reaction Type	Parameter estimation	Value / mM^{-1} h^{-1}
$k_{cond(NP\text{-}NP)}$	Hydrazide: NP-bound; electron rich<br>Aldehyde: NP-bound; electron rich	Estimate = 0.9× 0.67× $k_{cond(\mathbf{2})}$	0.4
$k_{cond(NP\text{-}L\mathbf{3}\text{-}NP)}$	Hydrazide: NP-bound; electron rich<br>Aldehyde: NP-bound; electron poor	Estimate = 0.9 × $k_{cond(\mathbf{1})}$	0.8
$k_{hydr(NP\text{-}NP)}$	Hydrolysis of interparticle links	Estimate slowest process	1×10^{-6}
$k_{hydr(NP\text{-}L\mathbf{3}\text{-}NP)}$	Hydrolysis of interparticle links	Estimate slowest process	1×10^{-6}
$k_{trans(10)}$	Hydrazide: NP-bound; electron rich<br>Benzylidine: NP-bound; electron rich<br>Leaving group: electron poor	Estimate = 0.8× $k_{trans(\mathbf{Sc})}$	0.5
$k_{-trans(10)}$	Hydrazide: molecular; electron poor<br>Benzylidine: NP-NP; electron rich<br>Leaving group: electron rich	Estimate = 0.5× $k_{trans(\mathbf{Sd})}$	0.9
$k_{trans(13)}$	Hydrazide: NP-bound; electron rich<br>Benzylidine: NP-bound; electron poor<br>Leaving group: electron rich	Estimate = 0.8 × $k_{trans(\mathbf{Sb})}$	0.2
$k_{-trans(13)}$	Hydrazide: molecular; electron poor<br>Benzylidine: NP-NP; electron poor<br>Leaving group: electron rich	Estimate = 0.5× $k_{trans(\mathbf{Sa})}$	0.06

7. References and notes

(1) Jouyban, A.; Soltanpour, S.; Chan, H. K. A Simple Relationship between Dielectric Constant of Mixed Solvents with Solvent Composition and Temperature. *Int. J. Pharm.* **2004**, *269*, 353-360.

(2) Bloomfield, V. A.; Dewan, R. K. Viscosity of Liquid Mixtures. *J. Phys. Chem.* **1971**, *75*, 3113-3119.

(3) Heller, W. Remarks on Refractive Index Mixture Rules. *The Journal of Physical Chemistry* **1965**, *69*, 1123-1129.

(4) Fulmer, G. R.; Miller, A. J. M.; Sherden, N. H.; Gottlieb, H. E.; Nudelman, A.; Stoltz, B. M.; Bercaw, J. E.; Goldberg, K. I. NMR Chemical Shifts of Trace Impurities: Common Laboratory Solvents, Organics, and Gases in Deuterated Solvents Relevant to the Organometallic Chemist. *Organometallics* **2010**, *29*, 2176-2179.

(5) Marro, N.; della Sala, F.; Kay, E. R. Programmable Dynamic Covalent Nanoparticle Building Blocks with Complementary Reactivity. *Chem. Sci.* **2020**, *11*, 372-383.

(6) Zheng, N.; Fan, J.; Stucky, G. D. One-Step One-Phase Synthesis of Monodisperse Noble-Metallic Nanoparticles and Their Colloidal Crystals. *J. Am. Chem. Soc.* **2006**, *128*, 6550-6551.

(7) Manea, F.; Bindoli, C.; Polizzi, S.; Lay, L.; Scrimin, P. Expeditious Synthesis of Water-Soluble, Monolayer-Protected Gold Nanoparticles of Controlled Size and Monolayer Composition. *Langmuir* **2008**, *24*, 4120-4124.

(8) Hoops, S.; Sahle, S.; Gauges, R.; Lee, C.; Pahle, J.; Simus, N.; Singhal, M.; Xu, L.; Mendes, P.; Kummer, U. *Bioinformatics* **2006**, *22*, 3067–3074.
